# Charge-mediated Cu^δ+^ sites in dimension-controlled covalent organic frameworks enable base-free continuous photothermal CO_2_ cyclization

**DOI:** 10.1093/nsr/nwaf350

**Published:** 2025-08-22

**Authors:** Xingwang Lan, Yize Zhang, Lu Chen, Riyuan Zhang, Haobo Xu, Guoyi Bai, Zhi-Ming Zhang

**Affiliations:** College of Chemistry and Materials Science, Key Laboratory of Chemical Biology of Hebei Province, Hebei Research Center of the Basic Discipline of Synthetic Chemistry, Institute of Life Science and Green Development, Hebei University, Baoding 071002, China; College of Chemistry and Materials Science, Key Laboratory of Chemical Biology of Hebei Province, Hebei Research Center of the Basic Discipline of Synthetic Chemistry, Institute of Life Science and Green Development, Hebei University, Baoding 071002, China; College of Chemistry and Materials Science, Key Laboratory of Chemical Biology of Hebei Province, Hebei Research Center of the Basic Discipline of Synthetic Chemistry, Institute of Life Science and Green Development, Hebei University, Baoding 071002, China; College of Chemistry and Materials Science, Key Laboratory of Chemical Biology of Hebei Province, Hebei Research Center of the Basic Discipline of Synthetic Chemistry, Institute of Life Science and Green Development, Hebei University, Baoding 071002, China; College of Chemistry and Materials Science, Key Laboratory of Chemical Biology of Hebei Province, Hebei Research Center of the Basic Discipline of Synthetic Chemistry, Institute of Life Science and Green Development, Hebei University, Baoding 071002, China; School of Chemistry and Chemical Engineering, Tianjin University of Technology, Tianjin 300384, China; Institute for New Energy Materials and Low Carbon Technologies, School of Materials Science & Engineering, Tianjin University of Technology, Tianjin 300384, China

**Keywords:** covalent organic framework, Cu single site, CO_2_ conversion, cyclization, photocatalysis

## Abstract

The structural topology of covalent organic frameworks (COFs) critically governs their catalytic performance. However, the structure–property correlation regarding dimension-dependent charge polarization at catalytic sites remains poorly understood. Herein, we describe a dimensional engineering strategy to tailor the local microenvironment of Cu sites through constructing phenanthroline-based COFs with distinct architectures (2D vs 1D). Systematic characterization and theoretical analyses reveal that the molecular flexibility and mesoporous channels in 2D COFs promote non-radiative transitions and mass transport, while their layered architecture enables light-controlled thermal modulation of Cu^δ+^ (1 ≤ δ ≤ 2) electronic states, synergistically enhancing substrate adsorption and activation. Remarkably, a Cu-2D-COF achieves near-quantitative yield (99.9%) in visible-light-driven carboxylative cyclization of propargylic amines with CO_2_ under base-free conditions. We further engineered a continuous-flow photocatalytic system that demonstrates exceptional operational stability, delivering 2.143 g of pharmaceutical-grade 2-oxazolidinones with >95% purity. This work provides strategies for manipulating the local microenvironment through dimension-controlled frameworks in CO_2_ conversion.

## Teaser

Employing phenanthroline-COFs in a dimensional engineering strategy modulates Cud+ (1=d=2) sites, where 2D-COF promotes non-radiative transitions and mass transport while enabling light-controlled thermal modulation, facilitating base-free continuous photothermal CO_2_ cyclization.

## INTRODUCTION

Converting CO_2_ into value-added chemicals is a promising strategy for mitigating global climate change and addressing the energy crisis [1,2]. Among the diverse products derived from CO_2_ conversion [3–7], oxazolidinones produced by the carboxylative cyclization of propargyl amines with CO_2_ are particularly fascinating because of their important pharmaceutical value and high global market demand [8–11]. As a result, enormous efforts have been dedicated to developing catalysts to achieve this efficient conversion by conventional thermocatalytic methods [12]. To date, most reported catalysts are predominantly based on noble metals, such as Ag and Pd [13,14]. However, their high cost significantly hinders their large-scale industrial application in CO_2_ conversion. Earth-abundant Cu-based catalysts, especially low-valence Cu(I) [15–17], demonstrate high CO_2_ activation capability and catalytic activity for CO_2_ conversion. Thus, it seems likely that Cu catalysts represent strong candidates for CO_2_ cyclization [18,19]. In this field, Zhao, Astruc and their co-workers have respectively reported Cu_2_O@ZIF-8 [18] and Cu(I)-GSH/ZIF-8 [19] as heterogeneous catalysts in the cyclization reaction of propargylic amines with CO_2_. However, low-valence Cu(I) readily undergoes natural oxidation or reconstruction because of its unavoidable self-oxidative nature during the reaction process, resulting in reduced catalytic activity or stability. Current systems generally require stoichiometric bases (DBU, DABCO and Et_3_N, etc.) to form essential CO_2_-adduct intermediates [15,16,20–23], which are then relayed to substrates or intermediates to yield the desired products. These additives can also result in deactivating Cu sites through coordination poisoning, intrinsically suppressing catalytic activity and long-term stability, thereby impeding industrial viability. Moreover, strong basic additives also complicate purification processes with energy-intensive steps and generate secondary pollution. Therefore, developing Cu-based catalysts with integrated adsorption-catalysis functionality represents a critical yet challenging endeavor to simultaneously address the dual limitations of base dependency and stability in CO_2_ cyclization reactions.

Covalent organic frameworks (COFs), as crystalline porous polymers with predesigned topologies, have emerged as promising candidates for CO_2_ conversion due to their large surface areas, high chemical and thermal stabilities, tunable pore sizes and functionalities [24–26]. Their structural precision also enables periodic integration of active metal sites within the extended frameworks. This design not only prevents metal aggregation but synergistically enhances CO_2_ activation through cooperative interactions between metal sites and organic matrices [27]. Furthermore, well-defined metal coordination environments can be precisely constructed, enabling direct correlation between atomic-scale active site structures and catalytic properties. These advantages provide opportunities to regulate coordination geometry and electronic states of Cu catalysts in CO_2_ conversion. However, current research remains predominantly focused on two-dimensional (2D) COFs, which obscures the distinct contributions of dimensional attributes and impedes elucidation of intrinsic mechanisms. In principle, the spatial arrangement of COFs significantly influences their electronic properties and catalytic performance [28,29]. Compared to widely studied 2D COFs with high molecular flexibility [30–32], one-dimensional (1D) COFs exhibit distinct chain-packing behaviors through interchain π–π stacking, forming rigidified dual-edge architectures that restrict aromatic chain mobility while enhancing framework stability through thermodynamic optimization [33,34]. Despite these structural advantages, the dimensionality-dependent modulation of catalytic site electronic states and quantitative assessment of framework participation remain systematically unexplored. The precise engineering of charge distribution through dimensional control of networks is also rare and research is scarce on their photocatalytic applications due to synthetic challenges in obtaining isoreticular frameworks with varying topologies but identical active sites. This obstacle restricts the advancement of topology chemistry and impedes the investigation of structure–activity relationships. Therefore, exploring and uncovering dimensionally engineered Cu-based COF catalysts represent a pivotal breakthrough direction for this strategy.

**Scheme 1.**
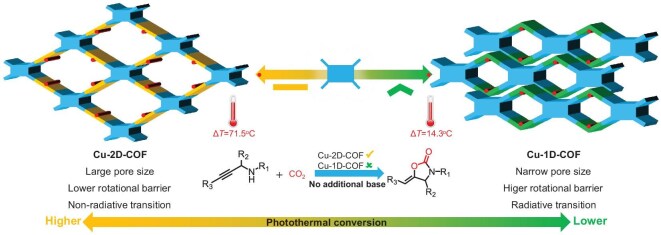
Schematic diagrams of Cu-2D-COF and Cu-1D-COF for photothermal CO_2_ cyclization.

As a proof-of-concept study, we herein designed crystalline 1D and 2D phenanthroline-based COFs by geometric regulation of the linkers, which featured distinct extended orientations and edge chains. The atomically dispersed Cu(II) species were anchored and oriented onto the pore walls of the COFs (Scheme 1). Full characterization confirms that compared to the constrained dual-chain edge structure of 1D-COF, the 2D-COF with molecular flexibility and extended π-conjugation exhibits dramatic non-radiative transitions, enhanced electron delocalization and more abundant low-frequency vibrational modes. This synergistic effect substantially boosts photothermal conversion efficiency, concurrently enabling spontaneous photoreduction of Cu(II) to stable low-valence Cu(I), generating a localized high-temperature basic catalytic microenvironment. Remarkably, the Cu-2D-COF exhibited exceptional catalytic activity for carboxylative cyclization of propargylic amines with CO_2_ under visible light, achieving a 99.9% yield without using strong bases. This surpasses most reported catalysts and marks the first documented instance of visible-light-driven cyclization. Additionally, a continuous-flow photocatalytic reactor was built for stable production of 2-oxazolidinones, yielding up to 2.143 g with high purity (>95%) under visible light and sunlight irradiation, which indicates practical application potential. This work presents an approach for modulating the local microenvironment of catalytic active sites with dimensionally controlled frameworks, providing a comprehensive strategy for designing high-performance CO_2_ cyclization systems.

## RESULTS AND DISCUSSION

### Synthesis and structural characterizations

**Figure 1.**
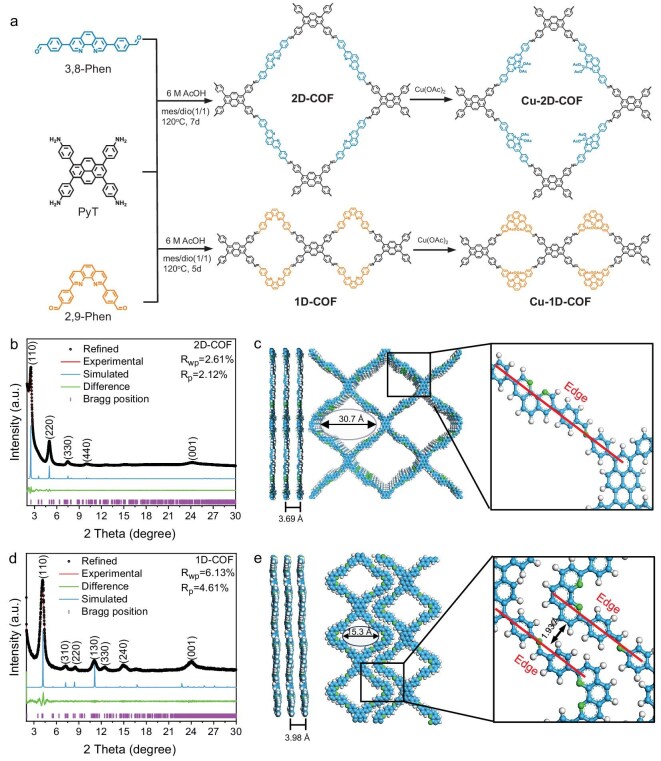
(a) Synthetic routes of Cu-2D-COF and Cu-1D-COF. (b and d) PXRD patterns of (b) 2D-COF and (d) 1D-COF. (c and e) Structure modes and detailed molecular fragments of (c) 2D-COF and (e) 1D-COF with layers arranged in an eclipsed AA-stacking mode. The blue, white and green spheres refer to carbon, hydrogen and nitrogen, respectively.

A 2D-COF and a 1D-COF were synthesized by a Schiff-base condensation reaction of 4,4′,4′′,4′′′-(pyrene-1,3,6,8-tetrayl)tetraaniline (PyT) with 4,4′-(1,10-phenanthroline-3,8-diyl)dibenzaldehyde (3,8-Phen) or 4,4′-(1,10-phenanthroline-2,9-diyl)dibenzaldehyde (2,9-Phen), respectively (Fig. 1a, for the details see Supporting Information in the Supplementary data). The chemical structures of the 2D-COF and 1D-COF were confirmed by Fourier transform infrared (FT-IR) and ^13^C cross-polarization/magic-angle spinning solid-state nuclear magnetic resonance (^13^C CP/MAS-ssNMR) spectroscopy. Fourier transform infrared spectra of both COFs showed that the stretching band at 1680 cm^−^^1^ corresponding to the C=O bond vibration of the aldehyde group in Phen and the stretching band at 3200–3500 cm^−1^ corresponding to the N–H bond vibration of PyT were dramatically attenuated (Fig. S1), implying the successful polycondensation between Phen and PyT. Meanwhile, a new vibration band was observed at ∼1620 cm^−1^, indicating the formation of imine C=N linkages. ^13^C CP/MAS NMR spectra demonstrated the peaks at ∼145 and ∼155 ppm from embedded pyridine ring carbon atoms and imine carbon atoms, respectively, further verifying the successful formation of imine bonds. The signals between 110 and 140 ppm corresponded to the carbon atoms in the benzene and pyrene rings (Fig. S2). In addition, thermogravimetric analysis (TGA) demonstrated that the 2D-COF and 1D-COF were thermostable up to 550°C with more than 95% weight maintained (Fig. S3), further confirming the polymerization of organic substrates. After loading single-site copper, FT-IR and ssNMR spectra of the Cu-2D-COF and Cu-1D-COF were similar to those of the pristine COFs (Figs S4 and S5). However, both the Cu-2D-COF and Cu-1D-COF exhibited mass losses in their TGA profiles (Fig. S6). The minor loss of <100°C, attributed to adsorbed water, and a major decomposition between 200°C and 300°C can be assigned to the thermal elimination of acetate groups from incorporated Cu salts. Nevertheless, the main framework still remained excellent thermostability of COFs.

The crystal structures of the 2D-COF and 1D-COF were investigated using powder X-ray diffraction (PXRD) measurements. As shown in Fig. 1b, the 2D-COF exhibited prominent diffraction peaks at 2.5°, 5.0°, 7.5°, 10.0° and ∼25°, which could be indexed to the (110), (220), (330), (440) and (001) facets, respectively. The structural simulation using an eclipsed AA-stacking model matched most of the experimental XRD peaks of the 2D-COF (Fig. 1c and Table S1). For the 1D-COF, diffraction peaks located at 4.1°, 7.2°, 8.5°, 11.0°, 12.5°, 14.9° and ∼25° corresponded to (110), (310), (220), (130), (330), (240) and (001) facets (Fig. 1d), respectively, which matched well with the eclipsed AA-stacking model (Fig. 1e and Table S2). Structural simulations elucidated that the 1D-COF exhibited intercalated multiple chain-like structures with an average chain distance of 1.93 Å. Notably, no obvious changes were observed in the PXRD patterns of the Cu-2D-COF and Cu-1D-COF compared with pristine samples, revealing that good crystallinity is still retained after the introduction of single-site copper (Figs S7–S8 and Tables S3–S4). Pawley refinement based on the simulated structures of both COFs agreed well with the experimental results (*R*_wp_ = 2.61% and *R*_p_ = 2.12% for the 2D-COF; *R*_wp_ = 2.30% and *R*_p_ = 1.68% for the Cu-2D-COF; *R*_wp_ = 6.13% and *R*_p_ = 4.61% for the 1D-COF; *R*_wp_ = 5.74% and *R*_p_ = 4.15% for the Cu-1D-COF). Nitrogen adsorption isotherms revealed the permanent porosities of these COFs, with both the 2D-COF and 1D-COF exhibiting distinct type I nitrogen adsorption isotherms (Fig. S9). The Brunauer–Emmett–Teller (BET) specific surface areas of the 2D-COF and 1D-COF were calculated to be 738.9 and 685.2 m^2^ g^−1^, respectively. The pore size distribution plots based on the non-local density functional theory (NL-DFT) demonstrate that the dominant pore sizes of the 2D-COF and 1D-COF were located at 3.32 and 1.68 nm (Fig. S10), consistent with the simulated models. The larger pore size of the 2D-COF facilitates mass transfer during catalytic reactions, which is favorable for boosting its catalytic performance. After introducing Cu centers, their specific surface area and pore volume decreased due to the increase in unit weight and the occupation of interior pores (Table S5).

**Figure 2.**
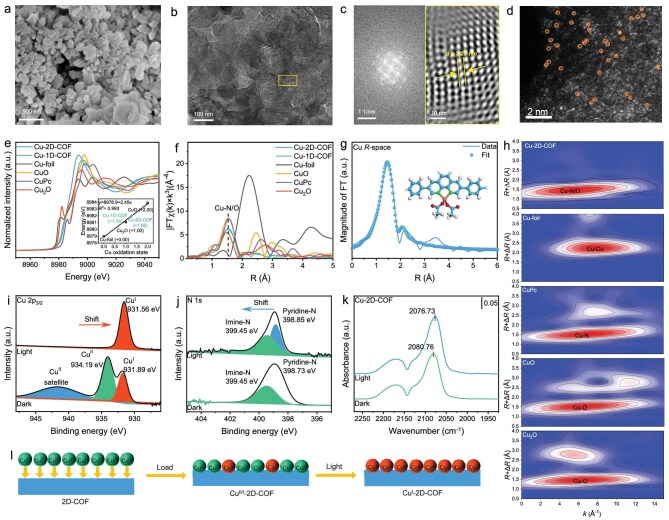
(a) SEM, (b) TEM, (c) corresponding Fast FT (FFT) pattern and reconstructed high-resolution TEM image and (d) AC HAADF-STEM images of Cu-2D-COF. (e) Cu K-edge XANES (inset is the valence state fitting calibration curve) and (f) FT-EXAFS spectra of Cu-2D-COF, Cu-1D-COF, CuPc, CuO, Cu_2_O and Cu foil. (g) EXAFS fitting curve of Cu-2D-COF at R space. (h) Cu K-edge wavelet transform EXAFS (WT-EXAFS) contour plots of Cu-2D-COF and reference samples. *In situ* XPS spectra of (i) Cu 2p_3/2_ and (j) N 1s in Cu-2D-COF tested in the dark and under light irradiation. (k) *In situ* DRIFTS spectra of CO adsorption on Cu-2D-COF in the dark and under light irradiation. (l) Schematic illustration of valance change of Cu species in Cu-2D-COF.

Scanning electron microscopy (SEM) and transmission electron microscopy (TEM) images revealed that the 2D-COF and 1D-COF crystallized as irregular bulk aggregates (Fig. S11a and b). The Fourier transform image of TEM for the circled place images further revealed ordered lattice fringes of COFs, highlighting their regular pore structure and high crystallinity. The measured lattice spacings of 3.28 and 1.59 nm correspond to the (110) planes of the 2D-COF and 1D-COF, respectively, which align well with both the XRD data and the proposed structural models. Notably, the morphology, particle size and lattice ordering of the Cu-2D-COF and Cu-1D-COF remained unchanged compared to their pristine counterparts (Fig. 2a–c and Fig. S11c), demonstrating that the structural integrity of the COFs was preserved upon Cu incorporation. The energy-dispersive spectroscopy (EDS) elemental mapping image revealed that C, N, O and Cu elements were uniformly distributed throughout the COF matrix (Figs S12 and S13). Inductively coupled plasma optical emission spectroscopy (ICP-OES) quantified Cu contents at 4.2 wt% for the Cu-2D-COF and 3.6 wt% for the Cu-1D-COF, corresponding to coordination of 51.8% and 44.4% of phenanthroline units in the respective frameworks. To verify the single-atom dispersion of Cu species, the aberration-corrected high-angle annular dark-field scanning transmission electron microscopy (AC HAADF-STEM) was conducted on the Cu-2D-COF (Fig. 2d). The isolated dots highlighted by orange circles were attributed to Cu single atoms, confirming the single-atom dispersion of Cu centers in the2D-COF.

X-ray absorption spectroscopy (XAS) was conducted to deeply understand the local coordination environment and electronic states of Cu centers in the Cu-2D-COF and Cu-1D-COF. The Cu K-edge X-ray absorption near-edge structure (XANES) spectra and their first-derivative curves (Fig. 2e and Fig. S14) revealed that the absorption edge energy of both the Cu-2D-COF and Cu-1D-COF resided between those of Cu_2_O and CuO. These results indicated an oxidation state between Cu^+^ and Cu^2+^ (denoted as Cu^δ+^, where 1 *<* *δ* *<* 2). The valence state fitting curve further confirmed their average valence state of +1.82 and +1.24 for the Cu-2D-COF and Cu-1D-COF, respectively (Fig. 2e, inset). Moreover, Fourier-transformed (FT) Cu-edge extended X-ray absorption fine structure (EXAFS) spectra (Fig. 2f and Fig. S15) exhibited a dominant peak at 1.52 Å for both Cu-2D-COF and Cu-1D-COF, attributed to the Cu–N/Cu–O scattering path. Critically, the peaks of Cu–Cu bond at 2.54 Å in the EXAFS spectra of copper foil could not be observed, ruling out metallic clusters or dimeric Cu species. The existence of a Cu–N/Cu–O scattering path was further confirmed by the wavelet transform contour plots of Cu K-edge weighted EXAFS (Fig. 2h). The maximum WT intensity at 7.90 Å^−1^ of Cu–Cu in Cu foil could not be detected in the Cu-2D-COF, further indicating the atomic dispersion of Cu centers rather than the presence of Cu–Cu species. The primary scattering positions of the Cu-2D-COF are aligned between CuPc and Cu_2_O, supporting a predominant Cu–N/Cu–O coordination environment [35,36]. Extended X-ray absorption fine structure fitting results revealed that the copper centers in both the Cu-2D-COF and Cu-1D-COF exhibit a coordination number of approximately 4.1, indicating a tetracoordinated Cu center with two nitrogen and two oxygen atoms (Cu–N₂O₂ configuration) with an average bond length of ∼1.95 Å [37] (Fig. 2g, Fig. S16 and Table S6).

X-ray photoelectron spectroscopy (XPS) measurements were carried out to elucidate the electronic structure of Cu species. As shown in Fig. S17a, the high-resolution Cu 2p spectrum of the Cu-2D-COF and Cu-1D-COF exhibited two dominant peaks of Cu 2p_1/2_ and Cu 2p_3/2_ with satellite peaks, demonstrating the coexistence of Cu^2+^ and Cu^+^ ions [38–40], which was likely ascribed to electron injection from COFs into the Cu sites. The Auger LMM (L and M represent the L and M shells involved in the Auger electron transition) spectra of the freshly prepared Cu-2D-COF and Cu-1D-COF showed Cu^2+^ to Cu^+^ ratios of 3.7/1 and 7/4 with an average oxidation state of +1.79 and +1.63, close to the XANES results (Fig. S17b). The N 1s XPS spectra of the Cu-2D-COF were deconvoluted into two peaks at 399.46 eV for imine-N [41], and 398.73 eV for pyridinic-N [42]. The pyridinic-N peak exhibited a noticeable shift to a higher binding energy than the 2D-COF, indicative of metal–nitrogen coordination (Fig. S17c). Similar change trends were also observed for the Cu-1D-COF (Fig. S17d). The electronic structures of the two COFs were further examined by XPS, as shown in Fig. S18. In the N 1s high-resolution XPS spectrum of the 2D-COF, the deconvoluted peaks at 399.45 and 398.53 eV correspond to imine and pyridinic nitrogen species, respectively. While the 1D-COF exhibits analogous nitrogen species, binding energy shifts are observed, particularly for the imine nitrogen. This shift, coupled with a concomitant displacement of the imine carbon in C 1s spectra, collectively indicates edge microstructure-induced electronic modulation within the framework.

*In situ* XPS measurements were further conducted to probe the dynamic oxidation state changes of Cu species under illumination. High-resolution Cu 2p_3/2_ XPS spectra (Fig. 2i) showed that Cu^2+^ was almost entirely reduced to Cu^+^ upon light irradiation, suggesting the electron capture roles of Cu atoms in the Cu-2D-COF [43,44]. Specifically, Cu single atoms could trap the photogenerated electrons while photogenerated holes were located in the 2D-COF, which was beneficial for the accumulation of electrons on Cu sites to achieve the stabilized low valence state. Meanwhile, a positive shift (0.12 eV) of pyridinic-N binding energy occurred after light irradiation (Fig. 2j), which indicated the electron transfer from bidentate N atoms to Cu centers due to the formation of the Cu–N bridge. Furthermore, density functional theory (DFT) calculations were employed to model the electron transfer in the Cu-2D-COF. Molecular frontier orbitals of the simplified fragment showed that the highest occupied molecular orbital (HOMO) was mostly localized on the pyrene, while the lowest unoccupied molecular orbital (LUMO) was mainly localized on the Phen, indicating that pyrene and Phen function as the electron donor and acceptor, respectively (Fig. S19). Mulliken charge distribution showed that the Cu sites have positive potential, with a Cu(I) center (+0.524e) and a more positively charged Cu(II) center (+0.533e), while the N atoms in Phen showed a negative potential in the Cu-2D-COF (Fig. S20).

Furthermore, *in situ* diffuse reflectance infrared Fourier transform spectroscopy of CO adsorption (CO-DRIFTS) was conducted to elucidate the electron density of Cu in the Cu-2D-COF and Cu-1D-COF. As shown in Fig. S21a, compared with the pristine COFs, the spectra of both the Cu-2D-COF and Cu-1D-COF exhibited new peaks at 2080.76 and 2084.21 cm^−1^, respectively, which could be attributed to linear CO adsorption on the Cu. Interestingly, the generated Cu–CO peaks showed an obvious red shift under light irradiation (Fig. 2k and Fig. S21b), suggesting electron injection into the 2π* orbitals of CO from the photoexcited energetic Cu^+^ centers [45]. It is worth mentioning that the Cu-2D-COF presented a larger redshift (∼4.03 cm^−1^) compared to that of the Cu-1D-COF (∼2.12 cm^−^^1^), indicating a higher electron density at the Cu sites in the Cu-2D-COF [46–49]. Accordingly, it can be concluded that the COF framework stabilizes a Cu⁺-dominant oxidation state by modulating electron density around Cu sites via internal electric field-driven intramolecular charge transfer (Fig. 2l). Crucially, the extended π-conjugation in the 2D-COF enhances this electronic modulation effect relative to the 1D-COF, optimizing the electronic structure of Cu active sites for catalysis.

### Photothermal cyclization of propargylic amines with CO_2_

**Figure 3.**
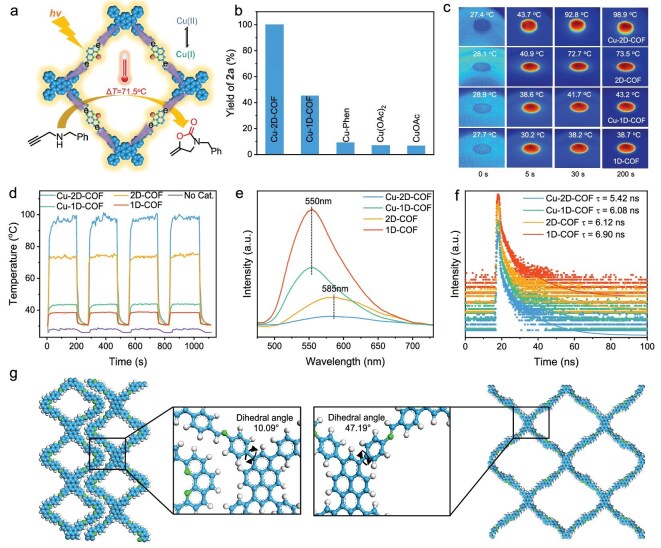
(a) Schematic photothermal carboxylative cyclization of **1a** with CO_2_. (b) **2a** yield with different catalysts under the same reaction condition (**1a** 0.5 mmol, catalyst 1.32 mmol% based on Cu, methanol 5 mL, 1 atm CO_2_, room temperature, 60 W white LED light for 60 min). (c) Photothermal images of different catalysts under different light irradiation times. (d) Cycling tests of temperature changes of different catalysts under irradiation. (e) PL and (f) time-resolution PL decay spectroscopy of different catalysts. (g) Simulated structures of 1D-COF and 2D-COF along with detailed molecular fragments of the selected regions.

The catalytic performances of as-synthesized COFs were investigated for light-driven carboxylative cyclization of propargylic amines with CO_2_ in the absence of any strong base. This reaction has been commonly accomplished by conventional thermocatalysis in the presence of strong bases. We initiated the reaction using *N*-benzylprop-2-yn-1-amine (**1a**) as the model substrate under ambient CO_2_ pressure and room temperature with visible light irradiation (Fig. 3a and Fig. S22). The Cu-2D-COF demonstrated exceptional catalytic performance, selectively producing 3-benzyl-5-methyleneoxazolidin-2-one (**2a**) with a remarkable yield of 99.9%, over 2-fold higher than that of the Cu-1D-COF (45.3%, Fig. 3b, Table S7). This pronounced performance gap underscores the superior catalytic efficiency of the 2D topology. However, **2a** could not be produced using the 1D/2D-COF without Cu sites or in the absence of a catalyst, whereas a poor yield was obtained when using an equal amount of Cu(OAc)_2_, CuOAc or Cu–Phen. Meanwhile, varying Cu loading can remarkably affect the activity of the Cu-2D-COF (Table S8). These results confirm that the Cu center is more likely the catalytic active site, and the π-conjugated framework of the 2D-COF is more favorable for promoting CO_2_ cyclization on the Cu sites. Additionally, a series of control experiments were performed to reveal the roles of reaction parameters. The amount of the Cu-2D-COF at 10 mg (1.32 mmol% based on Cu) achieved the highest yield. Solvent screening revealed that CH_3_OH was the optimal solvent for this system. No product was obtained when CO_2_ was replaced with an N_2_ atmosphere, suggesting the key role of CO_2_ in the carboxylative cyclization reaction. In addition, ^13^CO_2_ isotopic labelling experiments confirmed that the oxazolidinone product indeed originated from the reactant CO_2_ gas (Fig. S23). When feeding diluted CO_2_ (10% CO_2_/N_2_ in volume), the Cu-2D-COF still showed excellent catalytic activity with extended reaction time. These results indicate that finely tailored Cu sites within the 2D mesoporous COFs facilitated efficient CO_2_ adsorption and mass transfer, thereby serving as a favorable platform for low-concentration CO_2_ conversion.

In the presence of the Cu-2D-COF, the reaction was also feasible in the dark at room temperature, achieving a 24.6% yield for **2a**. However, this yield was significantly inferior to the 99.9% yield observed under photo-driven conditions. Nevertheless, subtle variations in reaction temperature (from 25°C to 65°C) could significantly enhance the catalytic efficiency in the dark (yields varied from 24.6% to 78.8%, Fig. S24). Therefore, it can be proposed that the catalyst provides the essential photo-activated driving force for CO_2_ activation and cyclization via localized photothermal effects [50]. These effects establish transient micro-reactors while maintaining bulk ambient conditions, synergistically enhancing reaction kinetics beyond pure thermal pathways through simultaneous energy-focusing and substrate pre-activation. To validate photothermal contributions, the photothermal effect of these COFs was recorded by monitoring the temperature variation on the catalyst surface under illumination (white LED light, 86 mW cm^−2^). After 200 s light exposure, the temperature difference (ΔT) of the 2D-COF reached 45.4°C, higher than the 11.0°C observed for the 1D-COF and 0.7°C for the blank (no catalyst) (Fig. 3c and Fig. S25). Upon Cu single-site incorporation, photothermal effects intensified significantly, with the Cu-2D-COF reaching 71.5°C ΔT. This synergy between the 2D framework and Cu sites generates substantial localized thermal energy under irradiation. Notably, during the photothermal test, both the Cu-2D-COF and Cu-1D-COF maintained the generated heat below 100°C under illumination. Since this range remains substantially below their decomposition thresholds (>220°C), structural damage from the photothermal effects is precluded. This stability was further confirmed through cycling tests, where both COFs exhibited consistent maximum surface temperatures over multiple irradiation cycles (Fig. 3d).

To unveil the reasons for the dimensionality-induced discrepancy of catalytic activity, a series of spectroscopic technologies were performed to investigate their photothermal effects. The solid-state ultraviolet-visible diffuse reflectance spectra (UV-vis DRS) (Fig. S26) revealed that the absorption band edge of the 2D-COF exhibited a significant redshift compared to the 1D-COF, which can be attributed to its larger 2D π-conjugated system [51,52]. Upon Cu site incorporation, the absorption further redshifted, broadening the absorption range and enhancing light-harvesting capability. Band gap calculations based on the Tauc plot confirm that the 2D-COF possesses a narrower band gap than 1D-COF, with a further reduction upon Cu doping (Fig. S27). Consistent with this, density of states (DOS) analysis reveals a closer proximity of the valence band edge to the Fermi level in the 2D-COF compared to the 1D-COF (Fig. S28). These results demonstrate that the transition from 1D to 2D topology can enhance electron delocalization, promote more efficient electronic transition upon photoexcitation, and thus elevate photothermal conversion efficiency [53,54].

Photoluminescence (PL) spectra (Fig. 3e) provide additional support for these findings. Specifically, the fluorescence emission peak of the 2D-COF exhibited a redshift compared to that of 1D-COF, accompanied by a significant reduction in fluorescence intensity. Furthermore, the fluorescence emission intensity decreased substantially for the Cu-1D-COF and was nearly completely quenched for the Cu-2D-COF compared to their respective 1D/2D-COF counterparts. These observations strongly indicate that non-radiative exciton relaxation dominates in the Cu-2D-COF, which is advantageous for heat generation [55]. The pronounced quenching of fluorescence in the Cu-2D-COF highlights its enhanced ability to dissipate energy through non-radiative pathways, thereby facilitating efficient thermal energy conversion. Time-resolved PL measurements showed that the Cu-2D-COF (τ_avg_ = 5.42 ns) accelerated decay kinetics and reduced fluorescence lifetimes, suggesting that the 2D π-conjugated system and Cu incorporation promote non-radiative relaxation pathways (Fig. 3f) [56]. The effective suppression of radiative recombination significantly reduces photon energy loss and enhances energy dissipation through molecular vibrational relaxation, resulting in superior photothermal conversion efficiency [55,57]. Additionally, the torsion angle of the benzene ring connected to the pyrene unit in the 2D-COF was calculated to be 47.19°, significantly larger than that of the 1D-COF (10.09°) (Fig. 3g), reflecting the structural differences between the two conjugate skeletons. The dual-chain-like edges of the 1D-COF facilitate the stabilization of aromatic backbones, while in contrast, the 2D-COF incorporates an extended 2D π-conjugated system, enabling enhanced molecular flexibility and rotational freedom. This structural advantage allows for improved electronic delocalization and stronger electron–phonon coupling, which promote non-radiative transitions and facilitate efficient heat dissipation [58,59]. Furthermore, the photocurrent response measurements of the 1D-COF displayed a higher photocurrent density than that of the 2D-COF (Figs S29 and S30), indicating superior photoinduced carrier generation. Following Cu incorporation, both materials exhibited enhanced photocurrent, yet the Cu-1D-COF still presented greater current density compared to the Cu-2D-COF. The electrical impedance spectra (EIS) test demonstrated that the Cu-1D-COF had a lower charge transfer resistance than the Cu-2D-COF, confirming accelerated interfacial kinetics in the 1D-COF. This aligns with the proposed mechanism where edge microstructures in the 1D-COF minimize non-radiative relaxation pathways, thereby directing energy toward carrier separation [60]. Despite superior charge dynamics, the Cu-1D-COF exhibited lower catalytic efficiency, confirming photothermal effects as the primary activity driver. Consequently, these results offer plausible explanations on the remarkably enhanced photocatalytic performance of the Cu-2D-COF over the Cu-1D-COF; from a photophysical perspective, the molecular flexibility of the 2D-COF promotes non-radiative transitions, generating exceptional photothermal energy. This localized thermal activation can lower the energy barrier for intermediate formation, thereby driving efficient CO_2_ carboxylative cyclization.

The stability of the Cu-2D-COF was subsequently assessed under catalytic conditions to evaluate its durability during the reaction process. During recycling tests, the Cu-2D-COF could be readily recovered and reused in subsequent cycles; the activity was well maintained for at least 10 cycles followed by an excellent yield of **2a** (Fig. S31). No obvious change can be observed in the FT-IR, PXRD and XPS spectra of the Cu-2D-COF after 10 cycles (Figs S32–S34), further proving its robust nature under the reaction conditions. After a hot filtration experiment, it was found that the reaction immediately ceased after removing the Cu-2D-COF (Fig. S35). Moreover, ICP-OES tests revealed that only 4.0 ppm Cu residue was detected in the filtrate. The above results confirmed that the Cu-2D-COF exhibited excellent intrinsic performance and robust features for the photothermal catalytic reaction. The green system without basic additives is also beneficial for improving the catalytic stability of the catalysts.

**Scheme 2.**
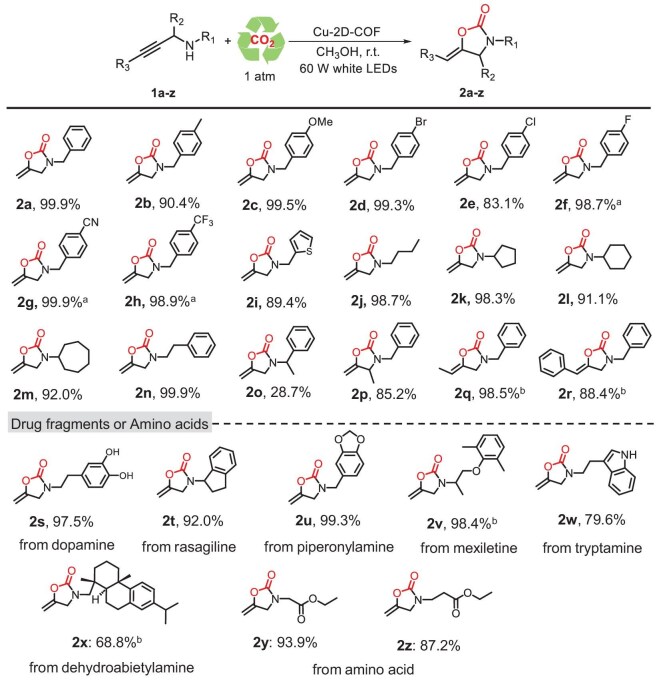
Substrate scope of propargylamine. Reaction conditions: substrate (0.5 mmol), Cu-2D-COF (10 mg, 1.32 mmol% based on Cu), CH_3_OH 5 mL, 1 atm CO_2_, 60 W white LED lamp for 60 min; isolated yield. ^a^2 h. ^b^4 h.

To explore the catalytic potential of the Cu-2D-COF, the substrate scope of propargylic amines was investigated under optimal conditions (Scheme 2). Terminal propargylic amines bearing electron-donating and -withdrawing groups in the *N-*phenyl ring proceeded smoothly to generate target products (**2a–2e**) with excellent yields. It was worth noting that the catalytic reaction became sluggish with substrates containing strong electron-withdrawing groups, such as F, CN and CF_3_; however, high yields (2f–2h) could be achieved by prolonging the reaction time. Propargylic amines with a thienyl or alkyl chain were well tolerated and converted to corresponding products (**2i–2m**) with high yields. Switching the benzyl group to benzylethyl substituent also afforded a 99% yield of **2n**. For the substrates containing a methyl group in the benzyl group, the reactions could proceed but with a low yield (**2o**) owing to the steric hindrance. By contrast, the presence of a methyl group adjacent to the terminal alkyne did not hinder the reaction, and the yield of product **2p** could reach 85.2%. Interestingly, after an extended reaction time, the internal alkynes, both methyl and phenylacetylene, could effectively react with CO_2_ to produce the corresponding products (**2q** and **2r**) in high yields. Meanwhile, the applicability of substrates was expanded by introducing a series of drug fragments or amino acid groups. The derivatives of drug molecules such as dopamine, rasagiline, piperonylamine, mexiletine and tryptamine could give target products in moderate to excellent yields (**2s–2w**). Bioactive molecules dehydroabietylamine (**2x**) could proceed but with a decreased yield of 68.8% by prolonging the reaction time, in which the steric hindrance considerably influenced the reaction effect. The ethyl ester of amino acids glycine (**2y**) and alanine (**2z**) could also deliver corresponding products in 93.9% and 87.2% yields, respectively.

### Mechanism study

To investigate the reaction mechanism, we conducted a series of control experiments by quenching possible active species under standard conditions (Fig. S36). KI, Mn(OAc)_3_ and 2,2,6,6-tetramethylpiperidoxyl (TEMPO) were used as scavengers to trap holes (h^+^), electrons (e^−^) and radicals, respectively. The yields of **2a** were greatly suppressed when adding KI and Mn(OAc)_3_ into the catalytic system, indicating the crucial roles of photogenerated holes and electrons in the photocatalytic process. However, the addition of TEMPO only resulted in a slight decrease of the **2a** yield to 83.4%, indicating that radicals were not the decisive intermediates in the reaction.

**Figure 4.**
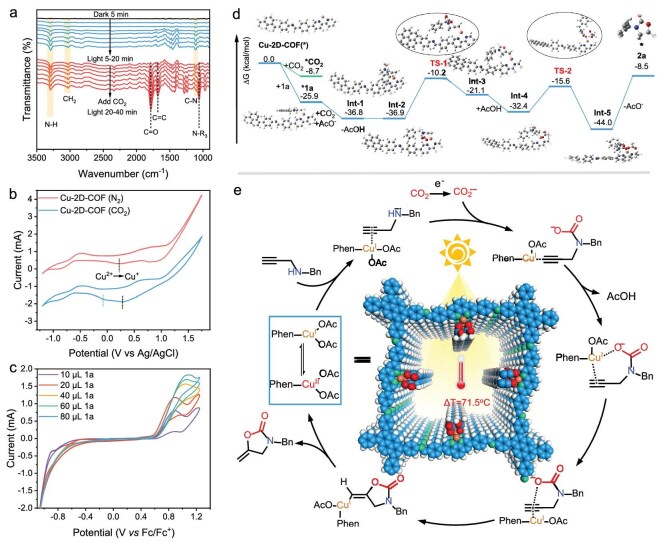
(a) *In situ* FT-IR of Cu-2D-COF with **1a** in a CO_2_ atmosphere in the dark and under light irradiation. (b) CV tests of Cu-2D-COF in a CH_3_OH solution containing 0.10 M TBAPF_6_ (TBA = tetra-*n*-butylammonium) under N_2_ or CO_2_ atmosphere. (c) CV tests for Cu-2D-COF with different concentrations of **1a** in CH_3_OH. (d) DFT-calculated Gibbs free energy profiles for the cyclization of **1a** with CO_2_ over Cu-2D-COF. (e) Proposed reaction mechanism.

Generally, the adsorption of CO_2_ on the surface active sites of the catalyst is responsible for its activation and subsequent conversion. To further explore the reaction pathway, *in situ* FT-IR was performed on the Cu-2D-COF and Cu-1D-COF with **1a** and CO_2_ atmosphere (Fig. 4a and Fig. S37). For the Cu-2D-COF, no obvious peaks were observed with **1a** in the dark. However, upon illumination, the characteristic peaks corresponding to N–H (3290 cm^−1^), C–N (1108 cm^−1^) and CH_2_ (3025 cm^−1^) stretching vibration bands of **1a** appeared, and the intensities of these peaks gradually increased with prolonged irradiation time, manifesting the continuous adsorption of **1a** over the Cu-2D-COF with reduced Cu centers. After 20 min light irradiation, CO_2_ gas was continually injected into the system. New absorption peaks related to product **2a**, including C=O (1780 cm^−1^), C=C (1680 cm^−1^), COO (1234 and 1278 cm^−1^) [61] and N–R_3_ (1060 cm^−1^) were observed and gradually increased as the cyclization reaction progressed, indicating that the carboxylative cyclization of **1a** and CO_2_ occurred on the catalyst surface. It should be noted that the characteristic stretching vibrations of N–COO^−^ (1419 and 1603 cm^−1^) [62] intermediates could be observed in the FT-IR spectra, revealing the formation of the carbamate. Moreover, physiosorbed linear CO_2_ at 2339 cm^−1^ could also be observed. Notably, although both materials exhibit comparable CO₂ adsorption intensities, the characteristic product peak and its corresponding intermediate peaks in the Cu-2D-COF showed greater intensity than those in the Cu-1D-COF. This catalytic activity divergence, emerging exclusively during product formation, demonstrates dimensionality-governed activation capability. Furthermore, upon exposure of the Cu-2D-COF under a CO_2_ atmosphere without **1a**, the characteristic peaks assigned to monodentate carbonate (m-CO_3_^2−^) (1730, 1675, 1540, 1415 and 1295 cm^−1^) and bidentate carbonate (b-CO_3_^2−^) (1655 and 1345 cm^−1^) were also observed, indicating that the Cu-2D-COF could enable local CO_2_ enrichment and then activate adsorbed CO_2_ molecules. However, no characteristic carbonate peaks were found in the FT-IR spectrum of the 2D-COF, suggesting the key role of Cu sites in the adsorption and activation of CO_2_ (Fig. S38). To further verify the CO_2_ adsorption, we performed CO_2_ adsorption tests on these materials. As shown in Fig. S39, the 2D-COF could efficiently take up CO_2_, and the introduction of Cu sites could improve the CO_2_ adsorption capacity (51.4 cm^3^ g^−1^). The above experimental results confirm that Cu species can act as the active sites for CO_2_ adsorption. Moreover, the CO_2_ adsorption capacity of the Cu-2D-COF surpassed that of the Cu-1D-COF due to its superior structural features. Unlike the Cu-1D-COF, their microporous structures limiting active site accessibility, the 2D mesoporous framework of the Cu-2D-COF provides a hierarchical pore structure that enhances the exposure of copper active sites. This improvement, combined with increased specific surface area and favorable pore size distribution, boosts CO_2_ adsorption performance, thereby promoting mass transfer during the reaction process.

Accordingly, the repeating unit of the Cu-2D-COF was used as a model in DFT calculations to verify the adsorption of CO_2_ and **1a** molecules on the Cu sites. According to *in situ* XPS results, the 2D-COF could absorb a photon and eventually transfer the photoexcited electron to Cu(II) associated with an internal electric field to generate Cu(I) through a thermodynamically favorable process (Fig. S40). Subsequently, the CO_2_ and **1a** molecules were absorbed on the Cu(I)-2D-COF to enable the following transformation. The adsorption of **1a** on the Cu site with an adsorption energy of −25.9 kcal/mol was more thermodynamically stable than the CO_2_ adsorption (−8.7 kcal/mol, Fig. S41). Moreover, the imine and pyrene moieties of the 2D-COF might also adsorb CO_2_ molecules (Fig. S42). Consequently, it was evidentially concluded that the Cu-2D-COF could potentially achieve targeted adsorption of **1a** and CO_2_ under light irradiation, where **1a** was more sensitive to Cu sites.

Cyclic voltammetric (CV) experiments were carried out to gain additional insight into photocatalysis. The Cu-2D-COF displayed an evident irreversible wave with an onset potential at 0.25 and 0.79 V vs Ag/AgCl in DMF under a N_2_ atmosphere, which could be assigned to the reduction peak of Cu species from Cu(II)/Cu(I) and Cu(I)/Cu(0), respectively (Fig. 4b) [63]. However, under the CO_2_ atmosphere, the Cu(I)/Cu(0) peak disappeared and a new current peak was observed at −0.07 V vs Ag/AgCl, suggesting the reaction of CO_2_ on Cu(I) species in the electrochemical process [64,65]. Moreover, the introduction of **1a** could cause an obvious oxidation peak, and the peak currents enhanced gradually with the increased concentration of **1a**, suggesting that **1a** could be oxidized by the holes generated in the Cu-2D-COF (Fig. 4c). Furthermore, Mott–Schottky measurements were conducted at different frequencies to estimate the energy level structures of these materials (Fig. S43). The detailed band arrangement of COFs is depicted in Fig. S44. Compared to the valence band position of the Cu-2D-COF [*E*_vb_ = +1.20 V vs the normal hydrogen electrode (NHE)], the more negative oxidation potential of **1a** (*E*_ox_ = +1.10 V vs NHE) thermodynamically supported the oxidation of **1a** through a hole attacking route [66].

Based on the above experimental results and previous reports [11,67,68], we proposed a possible reaction mechanism for the photothermal cyclization of propargylic amines with CO_2_ over the Cu-2D-COF (Fig. 4d and e). Under light irradiation, Cu(II) ions were initially reduced by photogenerated electrons on the 2D-COF to form Cu(I), enabling a great number of electrons to pass through the Cu centers and then favorably coordinate the alkynyl of the substrate. Meanwhile, the adsorbed CO_2_ accepted photogenerated electrons and the substrate N atom was oxidized to a radical cation by the hole. Due to the lack of strong bases, the dissociated free acetate from the catalyst possibly enabled the deprotonation of the substrate N atom. As a result, the N-carboxylation happened on alkyne-coordination *1a to produce the carbamate anion Int-1 and acetic acid. Density functional theory calculations indicated that Int-1 could isomerize to carbonyl-coordinated Int-2, which underwent intramolecular cyclization to give Int-3 through a five-membered ring transition state (TS-1) by overcoming a free energy barrier of 26.7 kcal mol^−1^. Subsequently, the complex Int-4 was produced by the weak interaction between Int-3 and free acetic acid. Int-5 was formed by capturing the proton from acetic acid via the TS-2 transition state with an energy barrier of 16.8 kcal mol^−1^. This process finally generated the target product with the release of acetate and catalyst.

### Continuous-flow system for the cyclization reaction

**Figure 5.**
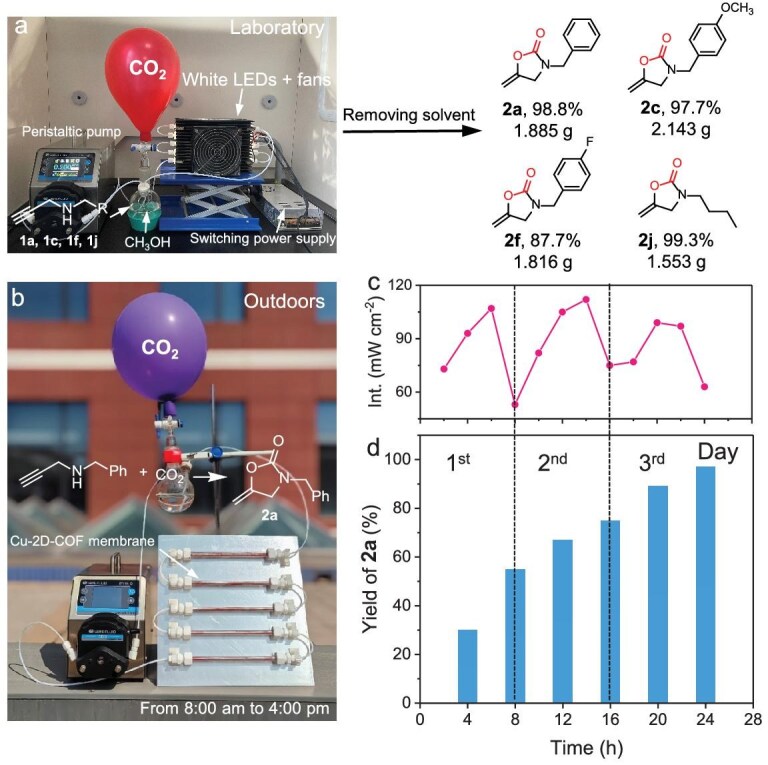
(a) Visible light-driven continuous-flow system with Cu-2D-COF membrane for the cyclization of propargylic amines with CO_2_. (b) Solar-driven continuous-flow system. (c) Solar intensity variation and (d) time-yield product plots for solar-driven cyclization of propargylic amines with CO_2_ over Cu-2D-COF photocatalyst membrane.

In order to realize the practical application of the cyclization of propargylic amines with CO_2_ to consecutively produce 2-oxazolidinones, we designed and built a photo-driven continuous-flow system composed of an integrated multichannel tubular reactor module and photocatalyst membrane (Fig. 5a and Fig. S45). The porous membrane was prepared by depositing the Cu-2D-COF on the aluminum foil using polyvinylidene fluoride as the binder. This method not only ensured efficient light absorption but also promoted substrate diffusion and maximized the utilization of catalytic active sites. Impressively, with visible light irradiation for 8 h, **1a**, produced on the gram scale (10 mmol, 1.45 g), achieved a 98.8% conversion rate in the continuous-flow system. In addition, it should be mentioned that a micro fixed-bed device filled with Cu-2D-COF powder was also constructed, where the reaction could be completed in 60 h (Fig. S46). This result highlights the higher efficiency of the tubular system with the photocatalyst membrane. Importantly, 1.885 g of product **2a** was obtained by simply removing the solvent, eliminating the need for additional post-treatment processes like column chromatography. The product purity reached approximately >95% (NMR purity), significantly simplifying the experiment process compared to previously reported batch reaction systems (Fig. S47). Impressively, the Cu-2D-COF photocatalyst membrane could still maintain over 95% conversion after six cycles of 48 h (Fig. S48); also, the membrane remained intact throughout the process (Fig. S49). Further, the substrate scope of propargylic amines can also be easily expanded to those containing electron-donating groups, withdrawing groups or alkyl chains. Remarkably, the corresponding products could be consistently produced in high yields (up to 2.143 g) in the continuous-flow system, demonstrating the potential practical application of a Cu-2D-COF-based flow-type photocatalytic reactor system.

To fulfill green and clean production, the cyclization reaction was further investigated under natural sunlight irradiation in the continuous-flow system (Fig. 5b). The outdoor continuous-flow system, equipped with a Cu-2D-COF membrane, was utilized for this model reaction of **1a** and CO_2_ for three consecutive sunny days from 13 May to 15 May 2024 (8:00 am to 4:00 pm) in Baoding City, Hebei Province, China. The outdoor sunlight intensity varied between 50 and 115 mW cm^−2^ (Fig. 5c). Remarkably, the corresponding product **2a** could still be obtained in 97% yield with high purity in sunny conditions (Fig. 5d). This indicated that the Cu-2D-COF could also efficiently and stably generate products in the continuous-flow reactor with natural sunlight irradiation. The continuous-flow system outperformed existing catalytic systems in both scale and catalytic efficiency, making it a promising candidate for practical applications in CO_2_ conversion to produce valuable chemicals using solar energy.

## CONCLUSION

In summary, this work presents a strategy of dimensionality-induced local microenvironment of single Cu sites through the construction of 2D and 1D phenanthroline-based COFs. Dimensional engineering in COFs is critical for enabling the Cu sites at pore channels. Compared to the interpenetrating chain-like framework of 1D-COFs, 2D-COFs possess enhanced molecular flexibility and mesoporous structures, which enable efficient non-radiative transitions, expose Cu active sites and allow precise modulation of Cu^δ+^ (1 ≤ δ ≤ 2) under local thermal environment by the photothermal effect, thereby improving substrate adsorption and activation. As a result, the Cu-2D-COF exhibited excellent catalytic performance for carboxylative cyclization of propargylic amines with CO_2_ under visible light, achieving a 99.9% yield without using strong bases. The catalytic efficiency by controlling the backbone from 1D to 2D was significantly increased. Moreover, a continuous-flow photocatalytic reactor was built to achieve continuous production of high-purity 2-oxazolidinones, yielding up to 2.143 g products over 8 h, demonstrating the potential practical application of Cu-2D-COFs. This work paves a new avenue for modulating the electronic structure of Cu sites in photothermal effect support for efficient CO_2_ conversion under solar energy.

## Supplementary Material

nwaf350_Supplemental_File
