# Supplementary material for: Charge-mediated Cuδ+ sites in dimension-controlled covalent organic frameworks enable base-free continuous photothermal CO2 cyclization
Source: Natl Sci Rev. 2025 Aug 22;12(11):nwaf350. doi: 10.1093/nsr/nwaf350 (PMC12576950; doi:10.1093/nsr/nwaf350)
Supplement: nwaf350_Supplemental_File [file nwaf350_supplemental_file.pdf]

Supporting Information

Charge-mediated $Cu^{\delta+}$ sites in dimension-controlled covalent organic frameworks enable base-free continuous photothermal CO_2 cyclization

Xingwang Lan,[*#] Yize Zhang,*[#] *Lu Chen, Riyuan Zhang, Haobo Xu, Guoyi Bai,*[*] *Zhi-Ming Zhang*[*]

X. Lan, Y. Zhang, L. Chen, R. Zhang, H. Xu

College of Chemistry and Materials Science, Key Laboratory of Chemical Biology of Hebei Province, Hebei Research Center of the Basic Discipline of Synthetic Chemistry, Institute of Life Science and Green Development, Hebei University

Baoding, Hebei, 071002, P.R. China

E-mail: lanxingwang@hbu.edu.cn (X. Lan)

[#]The authors contributed equally to this work.

G. Bai

School of Chemistry and Chemical Engineering, Tianjin University of Technology

Tianjin, 300384, P.R. China

E-mail: baiguoyi@hotmail.com (G. Bai)

Z-M. Zhang

Institute for New Energy Materials and Low Carbon Technologies, School of Materials Science & Engineering, Tianjin University of Technology

Tianjin, 300384, P.R. China

E-mail: zmzhang@email.tjut.edu.cn (Z-M. Zhang)

Table of Contents

Section 1. Materials characterization 3
Section 2. Supplement figures.. 16
Section 3. Supplement tables.. 38
Section 4. NMR data and spectra of oxazolidinones... 59
Section 5. Supporting References 90

Section 1. Materials characterization

1. Materials

All chemicals were commercially available and used without further purification. 3,8-Dibromo-1,10-phenanthroline (98%), 2,9-dibromo-1,10-phenanthroline (98%), 5,5'-dibromo-2,2'-bipyridyl (98%), 4-formylphenylboronic acid (98%), and 4-formylphenylboronic acid pinacol cyclic ester (98%) were purchased from Bide Pharmatech Ltd. Tetrakis(triphenylphosphine)palladium (99%), bis(triphenylphosphine)palladium(II) dichloride (98%), and $Cu(OAc)_2 \cdot H_2O$ (99%) were purchased from J&K Scientific Ltd. 3-Bromopropyne (96%) and benzylamine (98%) were purchased from Energy Chemical. 1,3,6,8-Tetrabromopyrene (97%), 4-aminophenylboronic acid pinacol ester, 2,2'-bipyridine (99%) and 1,10-phenanthroline (97%) were purchased Macklin Biochemical Technology Co., Ltd. Other solvents and reagents were purchased from local dealers and used without further purification.

2. Characterization

Fourier transform infrared (FT-IR) spectra were recorded on a Thermo Scientific Nicolet iS10 spectroscope. *In-situ* DRIFTS spectra were collected on a Nicolet iS10 Fourier transform infrared spectrometer equipped with a mercury cadmium telluride detector. The reaction chambers used kHVC-DRP-5 (HARRICK) equipped with two KBr windows and one quartz window. Powder X-ray diffraction (PXRD) patterns were measured by a Bruker D8 ADVANCE instrument with monochromatized Cu Kα radiation operating at 40 kV and 40 mA. Solid-state ^{13}C cross-polarization/magic-angle spinning solid-state nuclear magnetic resonance (CP/MAS ssNMR) spectra were collected on a Bruker AVANCE III HD 400MHz instrument. N_2 and CO_2 adsorption were measured at 77 K by Autosorb-iQ-MP adsorption analyzer after degassing at 100 ^{o}C for 12 h. Surface areas were calculated based on Brunauer-Emmett-Teller (BET) method. Pore size distribution curves were obtained via non-local density functional theory (NL-DFT) method. Scanning electron microscope (SEM) images were obtained on ZEISS Sigma 300. Transmission electron microscope (TEM) images were obtained on JEOL JEM 2100. Thermogravimetric analysis (TGA) was conducted from 25 ^{o}C to 800 ^{o}C under N_2 protection with a heating rate of 10 ^{o}C/min using a NETZSCH STA449C thermal analyzer. X-ray photoelectron spectroscopy (XPS) measurements were performed on the Thermo Scientific K-Alpha electron energy spectrometer with Al Kα (1486.6 eV) radiation as the X-ray excitation source. All binding energies were referenced to the C 1s peak

(284.8 eV) based on adventitious carbon. Cu K-edge analysis was performed with Si (111) crystal monochromators at the BL11B beamlines at the Shanghai Synchrotron Radiation Facility (SSRF) (Shanghai, China). Before the analysis at the beamline, samples were pressed into thin sheets 1 cm in diameter and sealed using Kapton tape film. The XAFS spectra were recorded at room temperature using a 4-channel Silicon Drift Detector (SDD) Bruker 5040. Cu K-edge extended X-ray absorption fine structure (EXAFS) spectra were recorded in transmission mode. Negligible changes in the line shape and peak position of Cu K-edge XANES spectra were observed between two scans taken for a specific sample. The XAFS spectra of these standard samples (Cu foil, CuO, Cu_2O and CuPc) were recorded in transmission mode. To obtain the quantitative structural parameters, collective data was processed by Athena and Artemis software from the IFEFFIT package. Steady-state photoluminescence and temperature-dependent photoluminescence spectra were recorded on a HITACHI F-7000 spectrophotometer. Time-resolved PL decay spectra were collected on an Edinburgh FS5 spectrophotometer. Inductively coupled plasma-optical emission spectrometry (ICP-OES) was carried out on an Agilent ICP-OES 730 instrument. Photothermal images were collected on Fluke TiX580 instrument.

3. Experimental procedures

3.1. Synthesis of 4,4'-(1,10-phenanthroline-3,8-diyl)dibenzaldehyde (3,8-Phen)

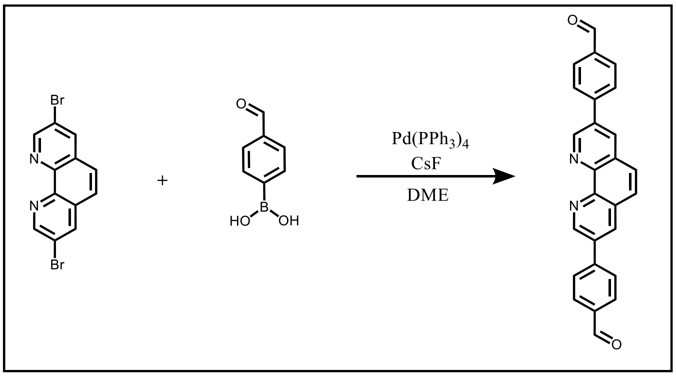

3,8-Dibromophenanthroline (0.5 g, 1.48 mmol) and 4-formylphenylboronic acid (0.6 g, 4.0 mmol) were suspended in 30 mL of DME and purged with N_2 for 30 min. Then, $Pd(PPh_3)_4$ (0.18 g) and CsF (1.1 g) were then added. The resulting mixture was stirred for 4 days at 110 ^oC under N_2 atmosphere. Upon cooling to room temperature, the resulting solid was filtered and subjected to Soxhlet extraction with chloroform for 3 days. After concentration, the collected product was washed with $CHCl_3$ and THF to afford 3,8-Phen as a white solid (415 mg, 72%). ^1H NMR (400 MHz, $CDCl_3$) δ 10.13 (s, 2H), 9.49 (s, 2H), 8.50 (s, 2H), 8.09 (d, *J* = 8.0 Hz, 4H), 8.03-7.87 (m, 6H). ^{13}C NMR (100 MHz, $CDCl_3$) δ 191.73, 149.39, 145.64, 143.35, 136.07, 134.72, 134.12, 130.66, 128.71, 128.24, 127.48.

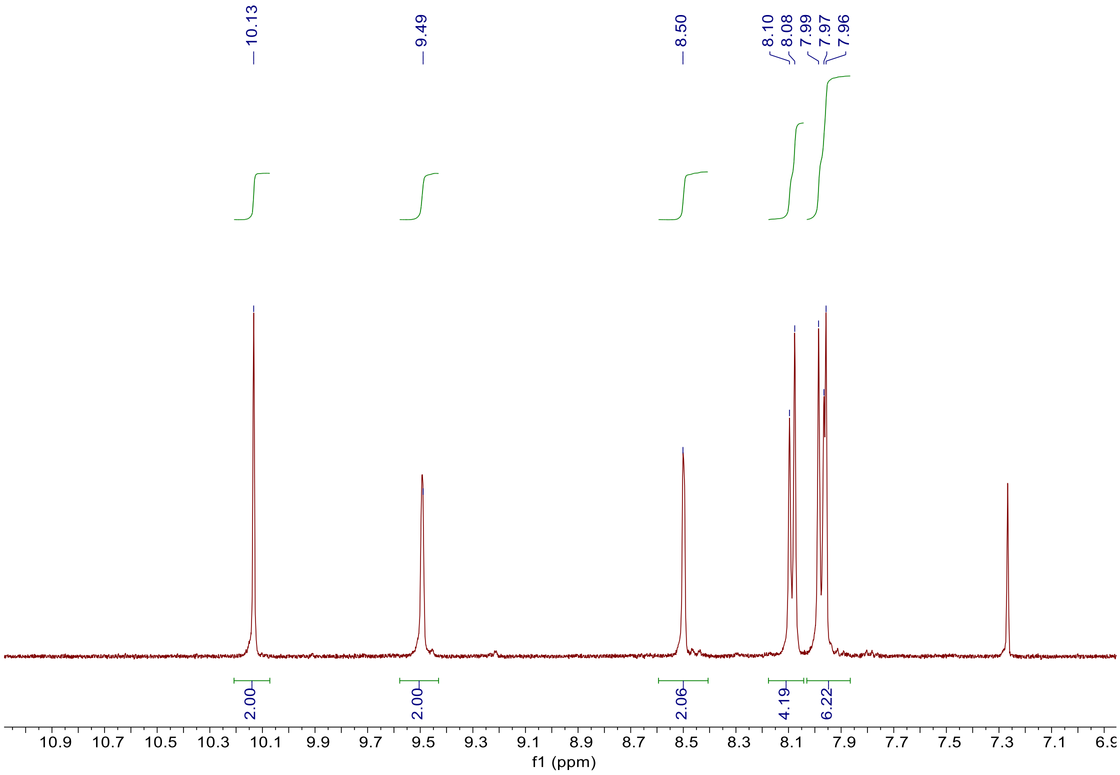

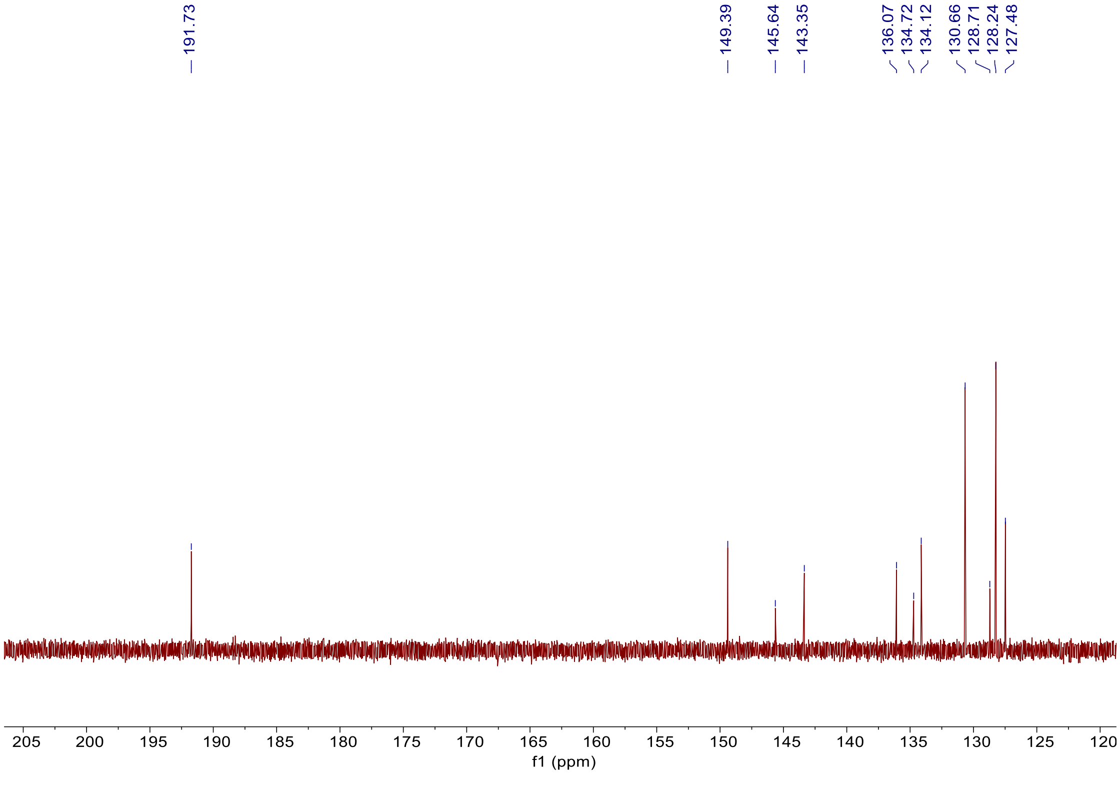

3.2. Synthesis of 4,4'-(1,10-phenanthroline-2,9-diyl)dibenzaldehyde (2,9-Phen)

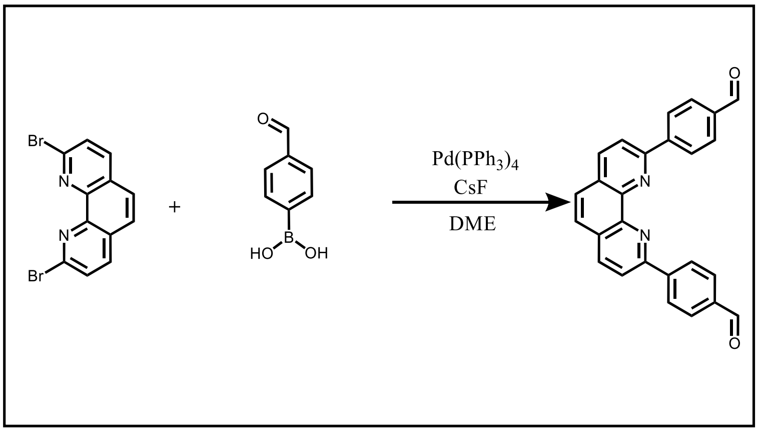

2,9-Dibromophenanthroline (0.5 g, 1.48 mmol) and 4-formylphenylboronic acid (0.6 g, 4.0 mmol) were suspended in 30 mL of DME and purged with N_2 for 30 min. Then, $Pd(PPh_3)_4$ (0.18 g) and CsF (1.1 g) were then added. The resulting mixture was stirred for 4 days at 110 ^{o}C under N_2 atmosphere. After cooling to room temperature, n-hexane was added to induce precipitation of the product. The precipitate was collected by filtration and subsequently washed with H_2O and CH_3OH, and finally recrystallized with dioxane to obtain 2, 9-phen as a gray solid (480mg, 84%). (480 mg, 84%).^{1}H NMR (400 MHz, $CDCl_3$) δ 10.12 (s, 2H), 8.68 (d, J = 7.8 Hz, 2H), 8.36 (d, J = 7.8 Hz, 4H), 8.05 (d, J = 7.8 Hz, 4H), 8.00 (d, J = 7.9 Hz, 2H), 7.90 (d, J = 7.7 Hz, 2H). ^{13}C NMR (100 MHz, $CDCl_3$) δ 192.19, 155.51, 146.25, 144.92, 137.40, 136.82, 130.38, 128.58, 128.26, 126.79, 120.70.

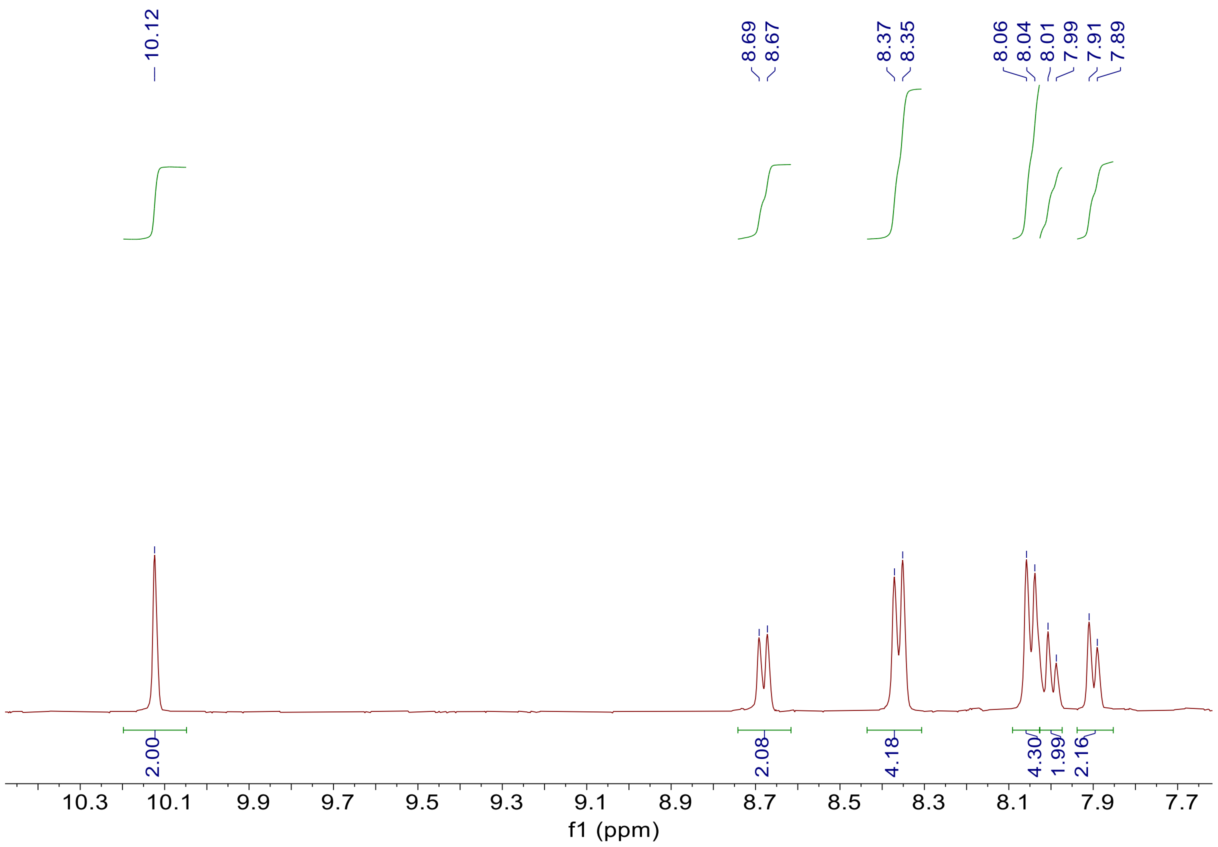

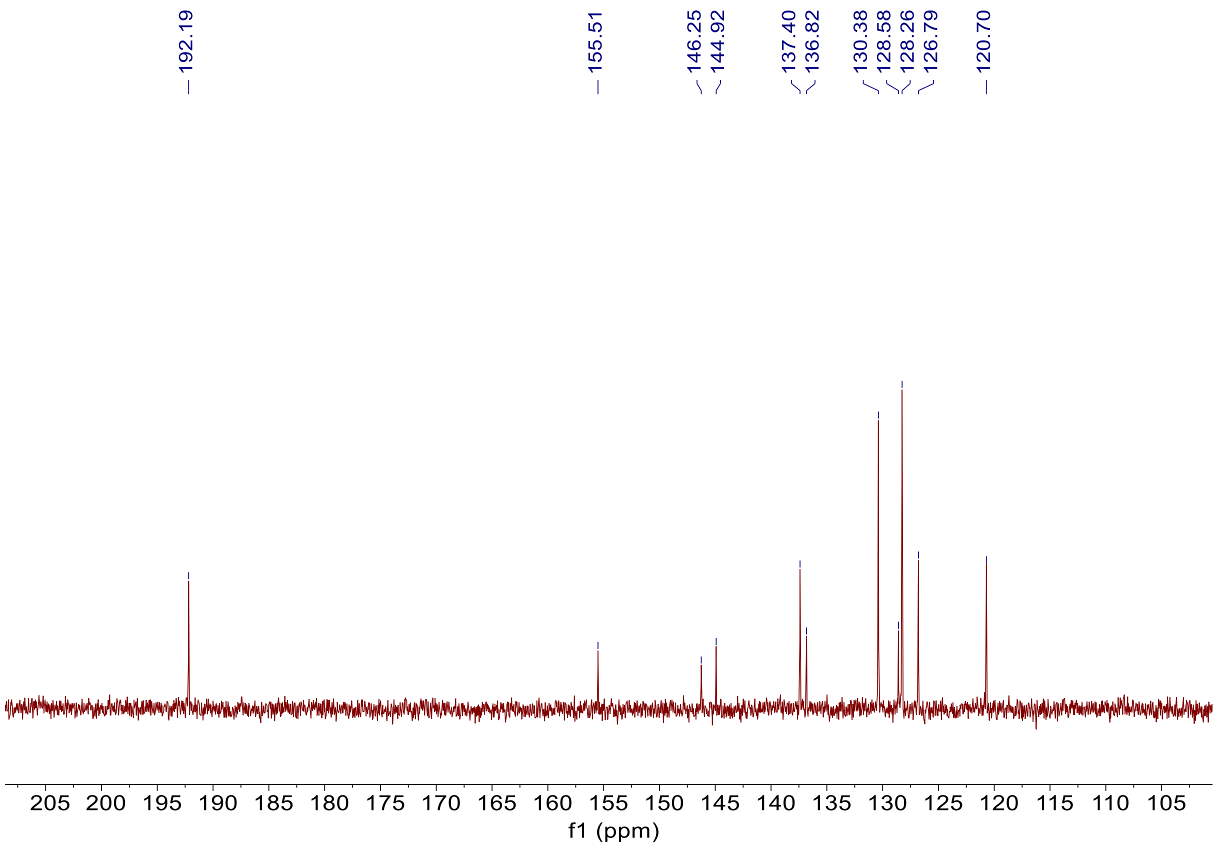

3.3. Synthesis of 4,4',4'',4'''-(pyrene-1,3,6,8-tetrayl)tetraaniline (PyT)

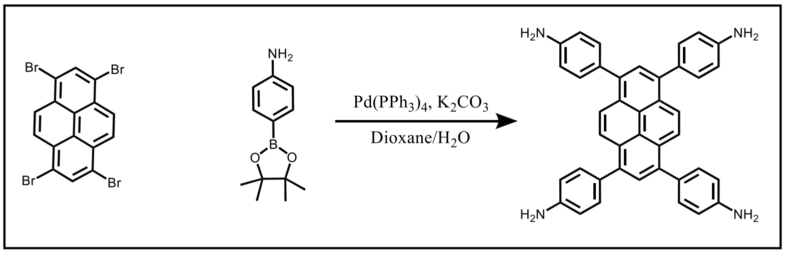

The PyT was synthesized according to previous literature with a slight modification.[1] 1,3,6,8-Tetrabromopyrene (1.5 g, 2.9 mmol), 4-aminophenylboronic acid pinacol ester (3.0 g, 13.7 mmol), K_2CO_3 (2.2 g, 15.7 mmol), and $Pd(PPh_3)_4$ (330 mg, 0.29 mmol) in were added to degassed dioxane/water (4/1, 50 mL). The mixture was heated at 115 ℃ for 3 days. Then it was cooled down to room temperature, and water (50 mL) was added leading to a precipitate which was collected by filtration and rinsed with water (50 mL) and CH_3OH (100 mL). The title compound was recrystallized from dioxane/hexane, and then dried under a high vacuum to yield the product, as a bright yellow powder (1.45 g, 89%). ^{1}H NMR (400 MHz, DMSO-d_6) δ 8.14 (s, 4H), 7.80 (s, 2H), 7.35 (d, J = 8.4 Hz, 8H), 6.78 (d, J = 8.3 Hz, 8H), 5.32 (s, 8H). ^{13}C NMR (100 MHz, DMSO) δ 148.73, 137.63, 131.56, 129.54, 128.07, 127.21, 126.63, 124.93, 114.44.

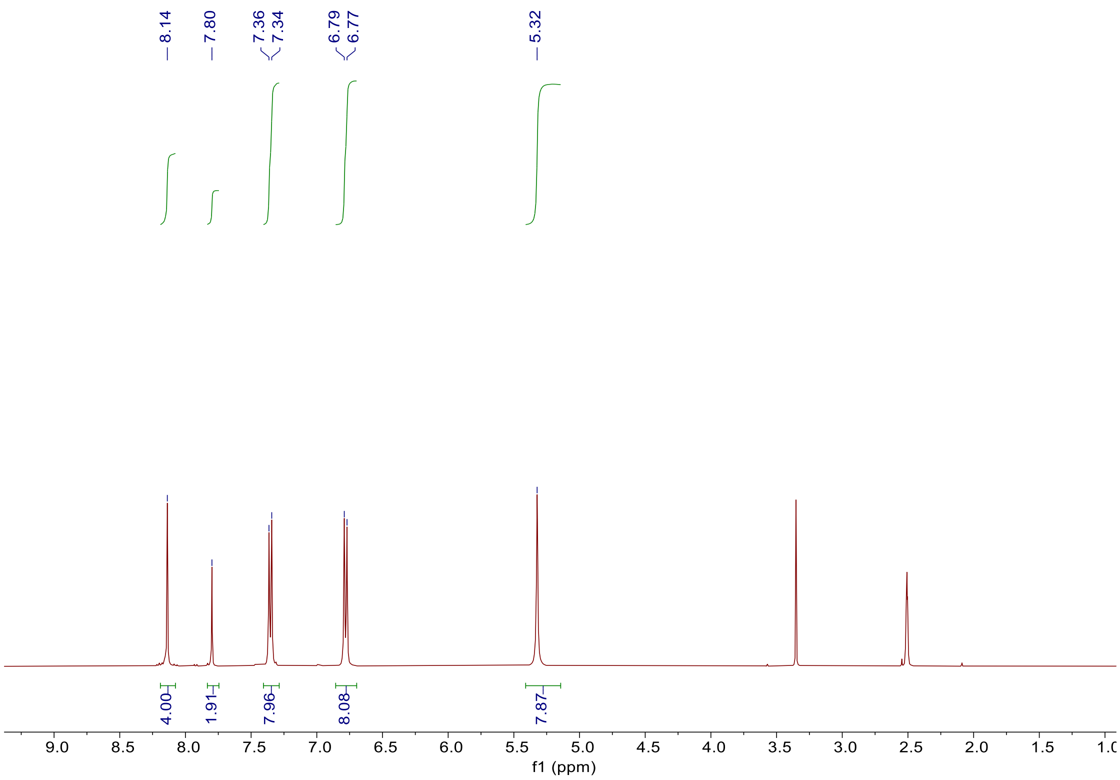

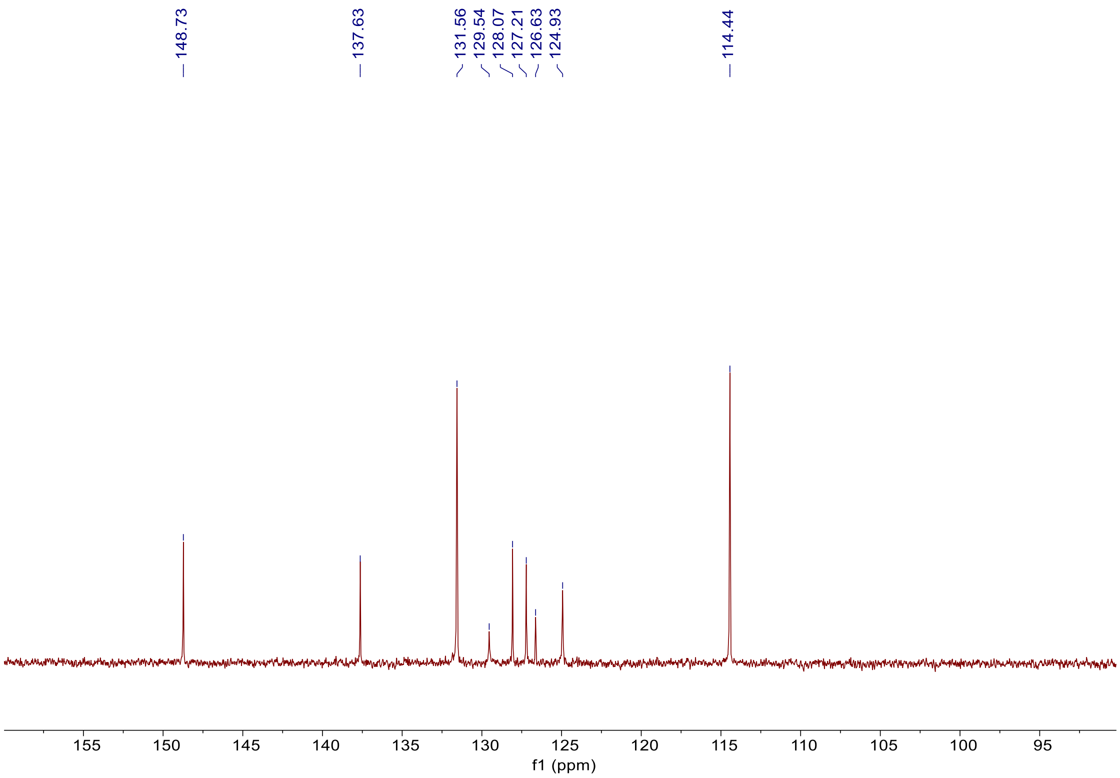

3.4. Synthesis of 2D-COF.

4,4',4'',4'''-(pyrene-1,3,6,8-tetrayl)tetraaniline (56.7 mg, 0.1 mmol) and 4,4'-(1,10-phenanthroline-3,8-diyl)dibenzaldehyde (77.7 mg, 0.2 mmol) were put into a thick-walled pressure tube equipped with a vacuum switch (15 mL). Then a mixed solution of Mesitylene and 1,4-Dioxane (6 mL, v/v = 1:1) was added and ultrasonically dispersed for 30 min. Subsequently, 0.6 mL of 6 M acetic acid (AcOH) was added to the above mixture and

sonicated again. After being sealed and degassed by three freeze-pump-thaw cycles, the mixture was heated and stirred at 120 °C for 7 days. After cooling to room temperature, the precipitate was collected by filtration, successively washed with excessive CH_3OH, THF, and acetone, and dried under vacuum at 60 °C for 12 h, finally affording the red solid with a yield of 90%.

3.5. Synthesis of 1D-COF.

4,4',4'',4'''-(pyrene-1,3,6,8-tetrayl)tetraaniline (56.7 mg, 0.1 mmol) and 4,4'-(1,10-phenanthroline-2,9-diyl)dibenzaldehyde (77.7 mg, 0.2 mmol) were put into a thick-walled pressure tube equipped with a vacuum switch (15 mL). Then a mixed solution of Mesitylene and 1,4-Dioxane (6 mL, v/v = 1:1) was added and ultrasonically dispersed for 30 min. Subsequently, 0.6 mL of 6 M acetic acid (AcOH) was added to the above mixture and sonicated again. After being sealed and degassed by three freeze-pump-thaw cycles, the mixture was heated and stirred at 120 °C for 5 days. After cooling to room temperature, the precipitate was collected by filtration, successively washed with excessive CH_3OH, THF, and acetone, and dried under vacuum at 60 °C for 12 h, finally affording the yellow solid with a yield of 89%.

3.6. Synthesis of Cu-2D-COF and Cu-1D-COF.

2D-COF (50.0 mg) and $Cu(OAc)_2{\cdot}H_2O$ (10.0 mg) were added to 15 mL CH_3OH, the resultant mixture was stirred for 12 h at 80 °C under N_2 atmosphere. After cooling to room temperature, the solid was filtered, washed thoroughly with CH_3OH, and dried under vacuum at 60 °C overnight to generate Cu-2D-COF. Cu-1D-COF was synthesized using the same procedures as that of Cu-2D-COF except 2D-COF was replaced by 1D-COF.

3.7. Synthesis of Cu-Phenanthroline complex.

Cu-Phen was synthesized according to previous literature with a slight modification.[2] $Cu(OAc)_2{\cdot}H_2O$ (798.6 mg, 4.0 mmol) was added into DMF(20 mL) and stirred to complete dissolution at room temperature. Then, 1,10-phenanthroline (360.4 mg, 2.0 mmol) dissolved in DMF(10 mL) was slowly injected into the above mixture and stirred overnight at room temperature. The precipitate was collected by filtration, washed with excessive ethyl ether, and dried under vacuum at 60 °C for 12 h, finally affording the blue solid.

3.8. Synthesis of propargylic amines

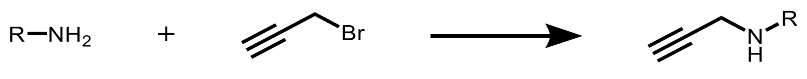

1a-1o, 1t-1x

Propargylic amines **1a-1o** and **1t-1x** were synthesized according to previous literature with a slight modification.[3] In a typical experiment, propargyl bromide (39.3 mmol) was added dropwise with 30 min to amines (162 mmol) in a 50 mL round bottom flask at 0 ℃. Afterward, the mixture was stirred at room temperature for 12 h. After completion, the resulting solution was diluted with 40 mL ethyl ether, and then the organic phase was washed with $NaHSO_4$ saturated solution (3 × 40 mL). After that collected organic phase was dried with anhydrous Na_2SO_4, the obtained organic phase was concentrated and further purified by column chromatography (PE/EA = 7/1 to 3/1), finally yielding yellow.

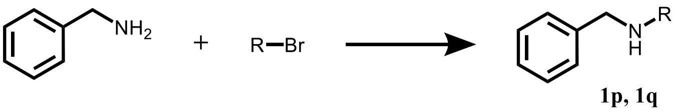

1p, 1q

Propargylic amines **1p** and **1q** were synthesized using the same procedures as that of Propargylic amines 1a except propargyl bromide was replaced by 3-bromobut-1-yne (**1p**) or 1-Bromo-2-butyne (**1q**).

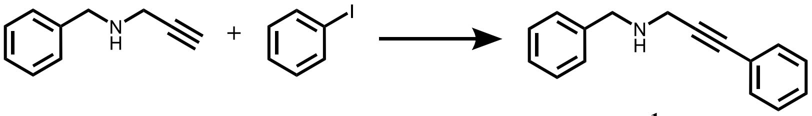

1r

Propargylic amine **1r** (10 mmol) was dissolved in THF (0.75 M), and added to a mixture of iodobenzene (10 mmol), CuI (0.4 mmol), and $PdCl_2(PPh_3)_2$ (0.2 mmol) in Et_3N (1.5 M). After 30 min of stirring at room temperature, the mixture was filtered through a plug of celite (washed with EtOAc) and concentrated under reduced pressure. The crude was added to a silica gel column and eluted with PE/EA to obtain the final product **1r**.

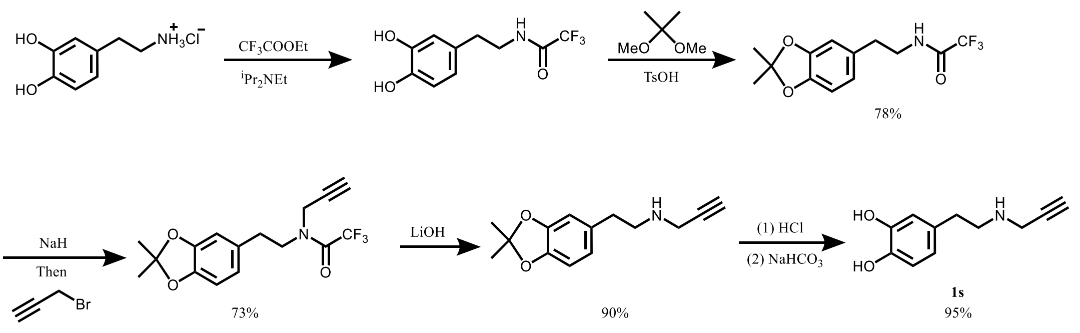

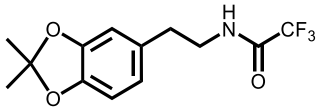

Dopamine hydrochloride (2.20 g, 11.6 mmol) and CH_3OH (12 mL) were added to a 100 mL round-bottomed-flask. After degassed by N_2 for 30 min, ethyl trifluoroacetate (2.0 mL,

16.8 mmol) and N,N-diisopropylethylamine (2.0 mL, 11.6 mmol) were added, respectively. The resulting mixture was stirred at room temperature for 30 h. Subsquently, the reaction was quenched with 1 N HCl, and the mixture was extracted with EtOAc. The combined organic layer was dried over Na_2SO_4, and evaporated to give a white solid, N-trifluoroacetyldopamine, which was used in the next reaction without any further purification. Subsequently, the as-obtained N-trifluoroacetyldopamine, 2,2-dimethoxypropane (5.69 mL, 46.4 mmol), $TsOH{\cdot}H_2O$ (0.23 g, 1.2 mmol), and benzene (120 mL) were added to a 300 mL round-bottomed-flask. The flask was connected to a dropping funnel filled with molecular sieves (4 Å), and a Dimroth condenser for efficient water removal. The mixture was stirred at 105 ^{o}C. After 40 h, the mixture was allowed to stand at room temperature, and the solvent was removed under reduced pressure. The mixture thus obtained was dissolved in CH_2Cl_2 and was passed through a silica gel plug. After removal of the solvent, a yellowish solid precipitated, which was filtered off and was washed with hexane to give a 78% yield (2 steps) of N-trifluoroacetyl-O,O'-isopropylidenedopamine. The spectrum was in a good agreement with the literature.[4]

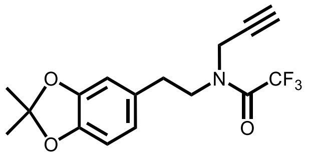

Sodium hydride (60 wt%, 4.2 mmol) and DMF (50 mL) were added to a three-neck flask and stirred at 0 ^{o}C for 10 min. Then, N-trifluoroacetyl-O,O'- isopropylidenedopamine (867 mg, 3.00 mmol) was added and stirred at 0 ^{o}C for 4 h. Propargyl bromide (264 µL, 3.3 mmol) was then added, and the mixture was heated to 50 ^{o}C and stirred for 16 h. After the reaction, the mixture was cooled to room temperature, and 100 mL of H_2O was added to quench the reaction. The mixture was extracted with dichloromethane, and the organic phase was dried over sodium sulfate (Na_2SO_4). After removing the solvent under reduced pressure, the product was further purified by column chromatography (PE/EA = 3/1), yielding yellow oil (70% yield). The spectrum was in a good agreement with the literature.[4]

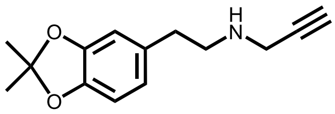

N-propargyl-N-trifluoroacetylO,O'-isopropylidenedopamine (350 mg, 1.07 mmol) and THF (7 mL) were added to a three-neck flask. Aqueous LiOH (1 M, 2.1 mL) was added to the solution and the mixture was stirred at room temperature for 15 h. Then, the reaction was

quenched by the addition of 1 N HCl at 0 ℃. After adding CH_2Cl_2, the mixture was extracted by water, and washed by CH_2Cl_2. After the aqueous phase was basified with saturated aqueous $NaHCO_3$, the solution was extracted by EtOAc. After dried over Na_2SO_4, the organic phase was concentrated under reduced pressure to give an 90% yield. The spectrum was in a good agreement with the literature.[4]

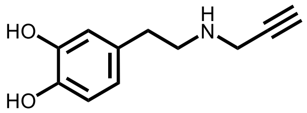

N-propargyl-O,O'isopropylidenedopamine (100 mg, 0.432 mmol) and dioxane (5 mL) were added to a three-neck flask and the mixture was stirred at room temperature until it became a solution. HCl (4 N, dioxane solution, 0.5 mL) was added and a white solid crashed out. Then, CH_3OH was added until the solid was fully dissolved, following which aqueous HCl (12 N, 1 mL) was added. After stirring at room temperature for 24 h. Then, the solvent was removed under reduced pressure.The oily crude compound thus obtained was dissolved in a tiny amount of CH_3OH and dropped into Et_2O to give a 95% yield of N-propargyldopamine hydrochloride as a white solid. The obtained product was dissolved in water, after the aqueous phase was basified with saturated aqueous $NaHCO_3$. The mixture was extracted with dichloromethane, and the organic phase was dried over Na_2SO_4. After that, the solvent was removed under reduced pressure to obtain the final product 1s. The spectrum was in a good agreement with the literature.[4]

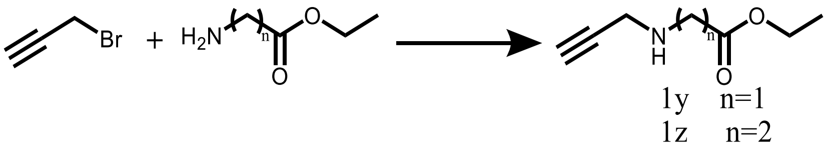

1y n=1
1z n=2

Propargylic amines **1y** and **1z** were synthesized according to previous literature with a slight modification.[5] Amine hydrochloride compounds (4.9 mmol) and potassium carbonate (1.3 g, 9.7 mmol) were added into acetonitrile (30 mL) and stirred at room temperature for 2 h to obtain a mixture. Propargyl bromide (0.57 g, 4.9 mmol) dissolved in acetonitrile (30 mL) was slowly injected into the above mixture at 0 ℃, and stirred overnight at room temperature. Finally, the reaction mixture was filtered to collect the filtrate. The filtrate was evaporated under reduced pressure to give the crude product, which was purified by column chromatography using PE/EA = 3/1 as eluent to obtain N-propargylated amino acids derivatives (**1y** and **1z**).

4. Photothermal catalysis of cyclization of propargylic amines with CO_2.

The photothermal catalysis of carboxylative cyclization of propargylic amines with CO_2 was conducted in a 100 mL self-designed quartz reactor with a water bath for controlling the temperature, as depicted in Figure S23. Before irradiation, the as-prepared catalyst (1.32 mmol% based on Cu) and propargylic amines (0.5mmol) were dispersed in CH_3OH (5 mL) and stirred by magnetic stirrer with a speed of 600 r/min in the reactor. Subsequently, the quartz reactor was sealed with a quartz glass top, purged, and filled with a CO_2 (99.999%) source. A 60W LED lamp (PLS-LED 100C, Beijing Perfectlight Technology Co., Ltd.) was used as a light source, where the light intensity was measured to be 86 mW cm^{-2} by light intensity meter (PL-MW2000, Beijing Perfectlight Technology Co., Ltd.). Maintain the reaction solution at a constant temperature of 25 ℃ using a thermostatic water bath (Figure S19). The reaction process was monitored using gas chromatography (GC, Agilent 7820A). After completion, the reaction mixture was filtered to remove the solid catalyst. The filtrate was evaporated under reduced pressure to give the pure product. All the pure products were identified by ^1H NMR and ^{13}C NMR.

5. Cyclization of propargylic amines with CO_2 in continuous flow system.

Propargylic amines (10 mol) were dissolved in 100 mL CH_3OH. The Cu-2D-COF membranes, containing 27 mg of Cu-2D-COF powder, were filled in 5 quartz tubes. The reaction solution was stored in a two-neck flask and purged by CO_2 for 30 min. A CO_2 balloon was then installed and connected to the tubular reactor via a peristaltic pump. CO_2 was then purged into the reaction system by excluding and charging with CO_2 several times. Then the solution was circulated within the tubular system via a peristaltic pump (0.5 mL/min) under white LED irradiation. The solution was collected and analyzed by GC at a given reaction time. After completion, the liquid was evaporated under reduced pressure to give the target product. All the target products were identified by ^1H NMR and ^{13}C NMR.

6. Photoelectrochemical measurements

All photoelectrochemical measurements were conducted by a CHI 760E electrochemical workstation via a standard three-electrode cell in 0.5 M Na_2SO_4 electrolyte solution. A saturated Ag/AgCl electrode was used as the reference electrode, and a Pt mesh was used as the counter electrode. The catalyst-coated F-doped SnO_2 (FTO) was used as working electrodes, which were prepared as per the following procedure: 2 mg sample was dispersed in dimethylformamide (480 μL) containing 5% Nafion solution (20 μL). The as-obtained mixture was ultrasonically dispersed for 30 min. Subsequently, 100 μL of the mixture was taken out and dropped on 1 × 1 cm^2 FTO and dried at room temperature. For EIS

measurements, the samples were tested with a frequency range from $1*10^6$ Hz to $1*10^2$ Hz. Mott-Schottky experimental plots were determined at frequencies of 800, 1000, and 1500 Hz.

7. Cyclic Voltammetry measurement

The Cyclic Voltammetry measurement was performed on a CHI 760E electrochemical work station (Chenhua Instrument, Shanghai, China) in a standard three-electrode system with the catalyst-coated F-doped SnO_2 (FTO) as the working electrode, Pt plate as the counter electrode, and an Ag/AgCl as a reference electrode. A 0.1 M TBAF in CH_3OH solution after deoxidation was used as electrolyte. Ferrocene was used as an internal standard and the ferrocenium-ferrocene (Fc^+ /Fc) potential (+0.40 V vs SCE in CH_3OH) was used to correct potentials vs SCE.

9. *In-situ* FTIR measurements

In-situ FTIR spectra were collected on a Nicolet iS 10 Fourier transform infrared spectrometer equipped with a mercury cadmium telluride detector. The reaction chambers used kHVC-DRP-5 (HARRICK) equipped with two KBr windows and one quartz window. The *in-situ* FTIR tests were conducted in two parts: (1) Chemical adsorption of propargylic amine. Before the measurement, the sample was treated at 120 ℃ under a flow of N_2 (50 mL/min) to remove adsorbed contaminants on the catalysts. After the reaction chamber was cooled to 298 K, the spectrum of the catalyst was collected as the background. Subsequently, **1a** was added to the bottom of the catalyst, and spectra were collected at different times under both dark and illuminated conditions (0-5 min in the dark, followed by 5-20 min under irradiation with a 60 W white LED lamp) to investigate the chemical adsorption behavior of the catalytic substrate. (2) Photothermal catalysis of the cyclization of **1a** with CO_2. After that, 5% CO_2 gas (20 mL/min) was introduced into the reaction chamber. The reactor was then irradiated with a 60 W white LED lamp for 0-20 min to investigate the cyclization of **1a** with CO_2.

Additionally, for CO_2 chemical adsorption tests, the reaction chamber was cleaned and a new sample was loaded. The sample was treated at 120 ℃ under a flow of N_2 (50 mL/min) to remove adsorbed contaminants on the catalysts. After the reaction chamber was cooled to 298 K, the spectrum of the catalyst was collected as the background. Then, 5% CO_2 gas (20 mL/min) was introduced into the reaction chamber, and spectra were collected at different times (0-30 min) to investigate the chemical adsorption behavior of the catalytic substrate.

10. Computational details

All computations were performed under the framework of Density Functional Theory (DFT) with Grimme's D3 correction using the Gaussian 09 software package of programs.[7] The simplified models were constructed using the smallest repetitive unit containing a pyrene and

phenanthroline. Copper sites were treated by removing the acetate groups. All the optimization calculations were performed with the B3LYP level of theory with SDD basis set for Cu atoms and 6-311G* basis set for the remaining atoms. Based on the optimized structures, the implicit solvent effects were considered via single-point calculations with the PCM model[8,9] and the methanol solvent (in accordance with the experiments), using the M062X functional.[10] The basis set of metal atoms was maintained while other elements were treated with 6-311+G(2df,p) basis set.[11]

Section 2. Supplemented figures

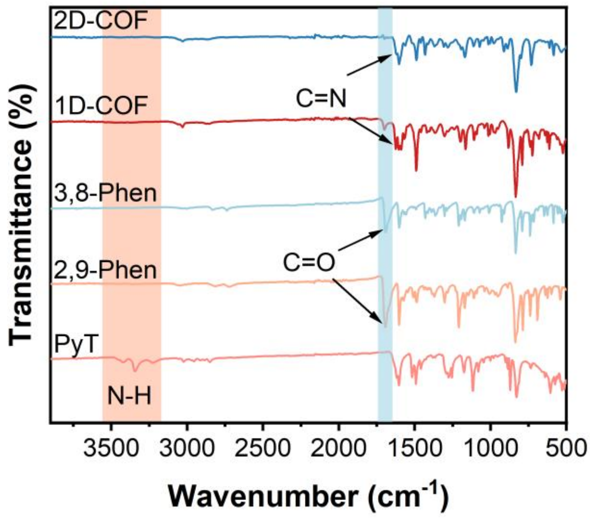

Figure S1. FT-IR spectra of 2D-COF, 1D-COF and corresponding precursors.

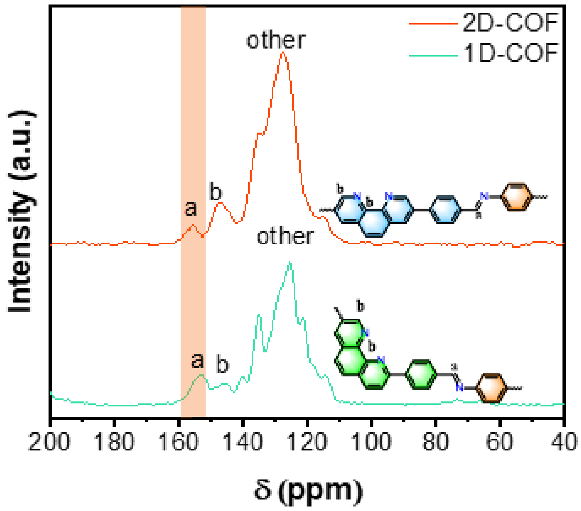

Figure S2. ^{13}C CP/MAS-ssNMR spectra of 2D-COF and 1D-COF.

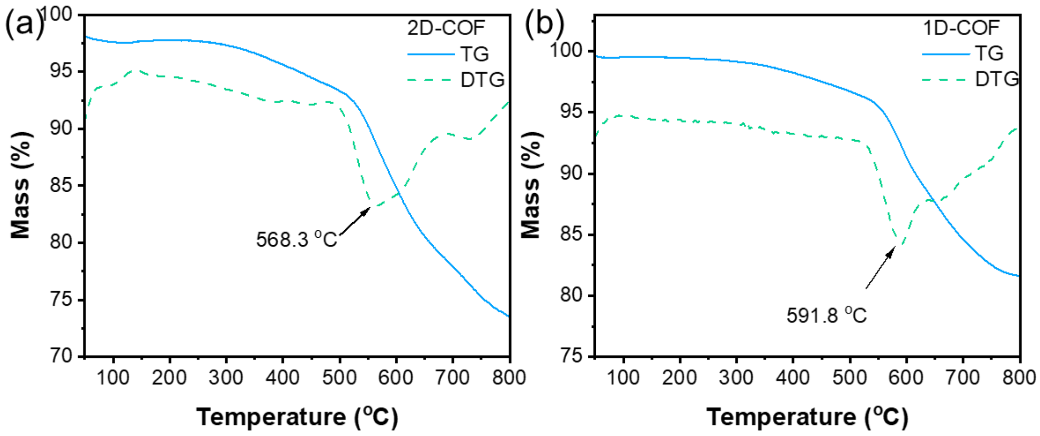

Figure S3. Thermogravimetric analysis of (a) 2D-COF and (b) 1D-COF.

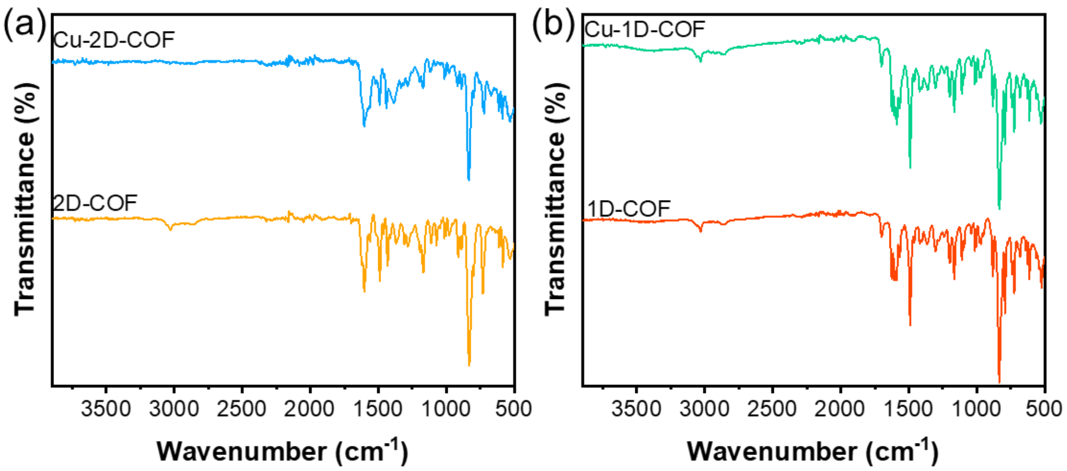

Figure S4. (a) FT-IR spectra of 2D-COF and Cu-2D-COF, (b) FT-IR spectra of 1D-COF and Cu-1D-COF.

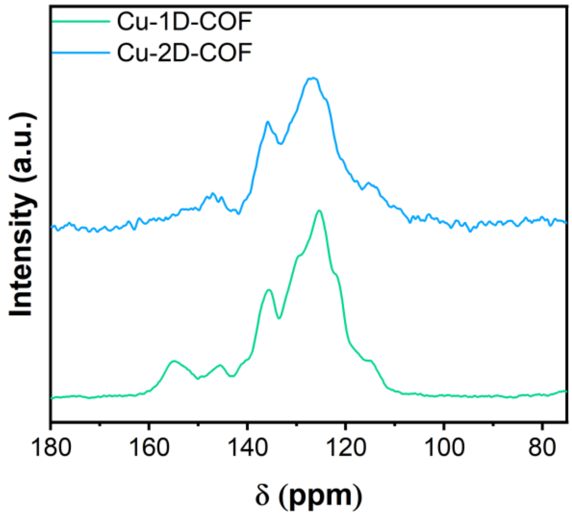

Figure S5. ^{13}C CP/MAS-ssNMR spectra of Cu-2D-COF and Cu-1D-COF.

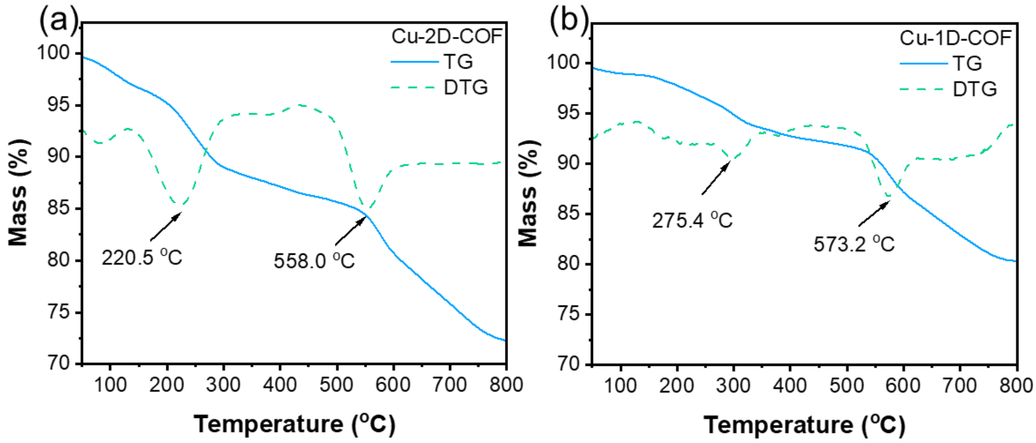

Figure S6. Thermogravimetric analysis of (a) Cu-2D-COF and (b) Cu-1D-COF.

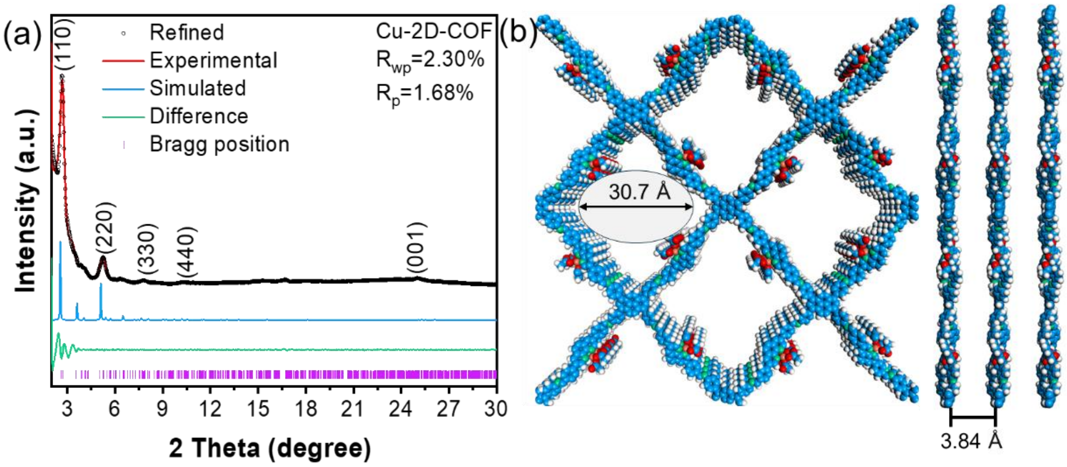

Figure S7. (a) PXRD pattern of Cu-2D-COF. Structure modes of (b) Cu-2D-COF with layers arranged in an eclipsed AA stacking mode. The blue, white, green, red, and orange spheres refer to carbon, hydrogen, nitrogen, oxygen and copper atoms, respectively.

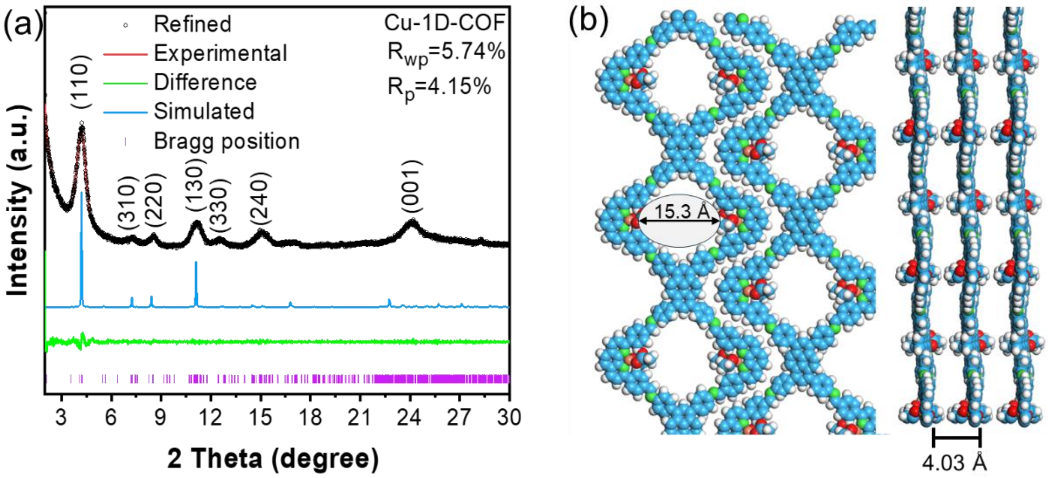

Figure S8. (a) PXRD pattern of Cu-1D-COF. (b) Structure modes of Cu-1D-COF with layers arranged in an eclipsed AA stacking mode. The blue, white, green, red, and orange spheres refer to carbon, hydrogen, nitrogen, oxygen and copper atoms, respectively.

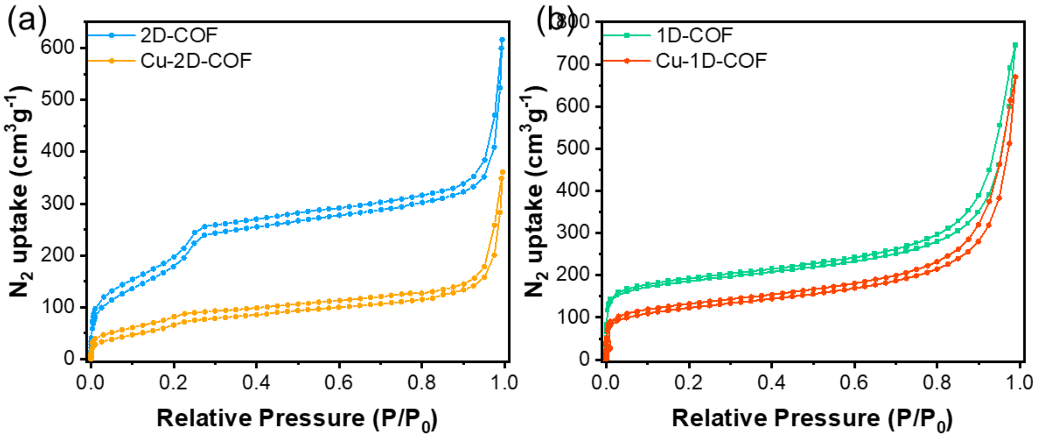

Figure S9. N_2 adsorption-desorption curves at 77 K of (a) 2D-COF and Cu-2D-COF, (b) 1D-COF and Cu-1D-COF.

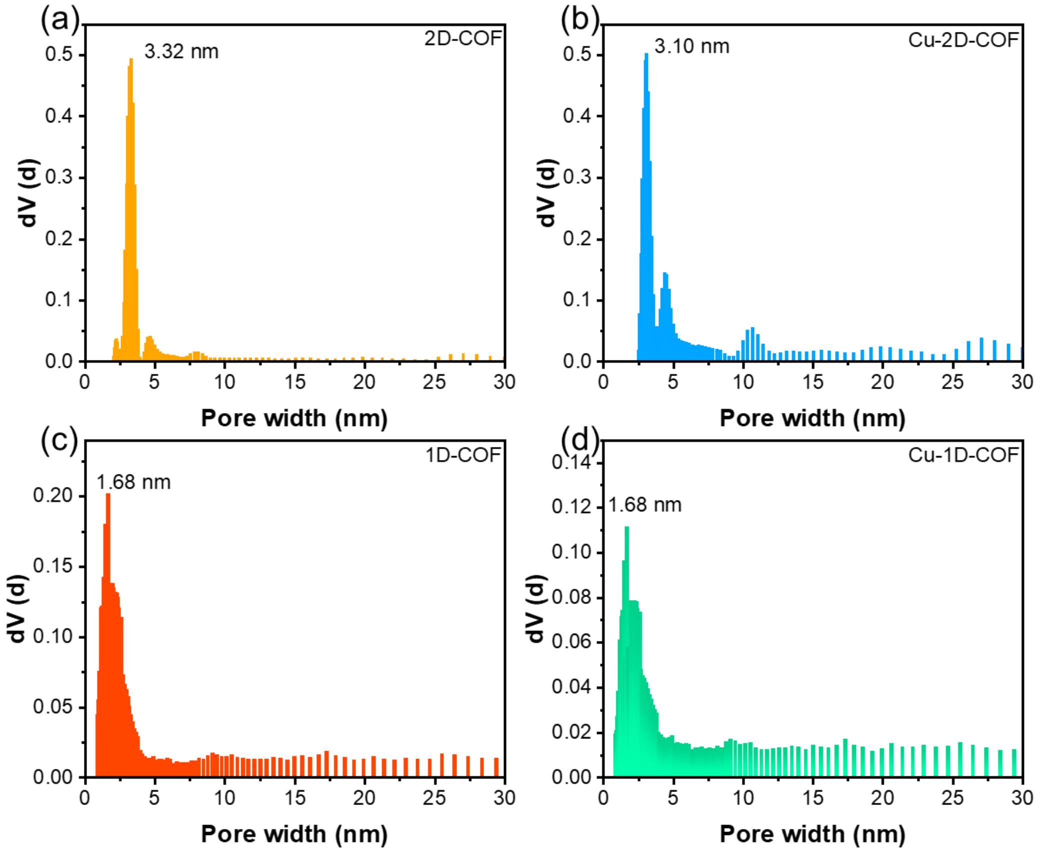

Figure S10. Pore size distributions of (a) 2D-COF, (b) Cu-2D-COF, (c) 1D-COF and (d) Cu-1D-COF.

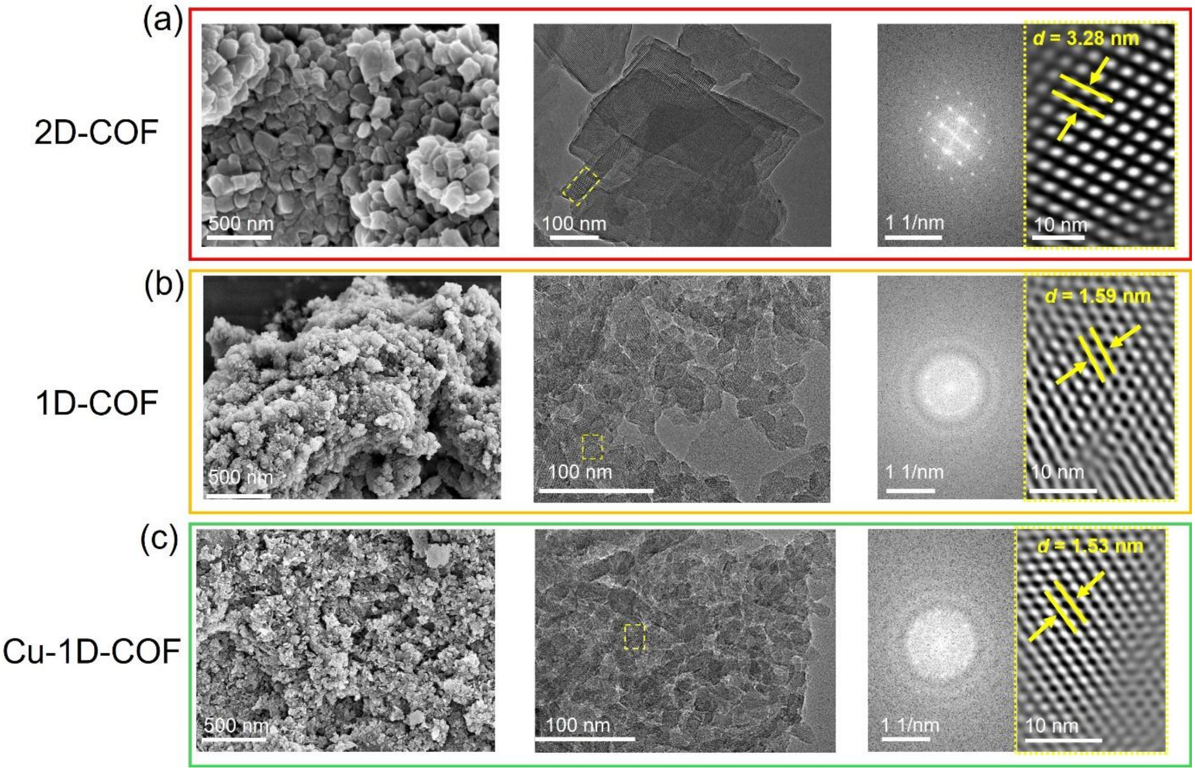

Figure S11. SEM, TEM images, and corresponding FFT pattern and reconstructed HRTEM images of (a) 2D-COF, (b) 1D-COF, and (c) Cu-1D-COF.

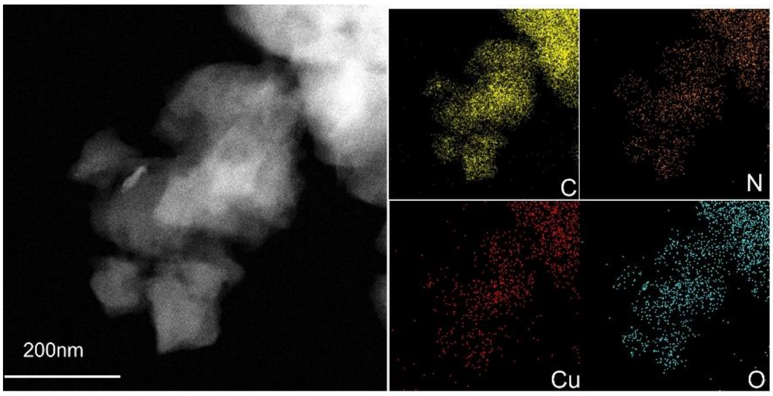

Figure S12. EDX elemental mapping of Cu-2D-COF.

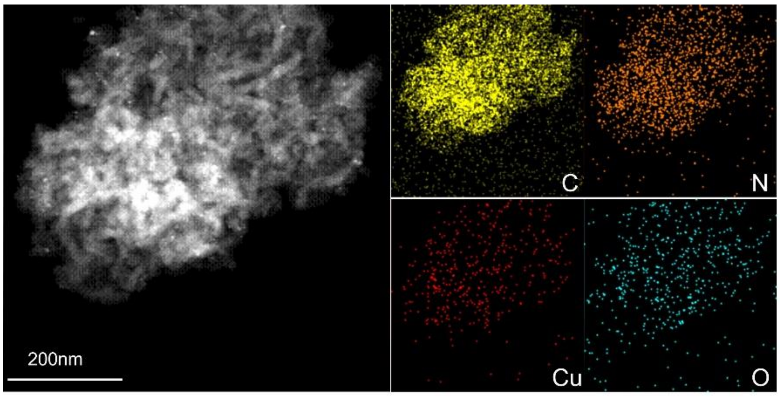

Figure S13. EDX elemental mapping of Cu-1D-COF.

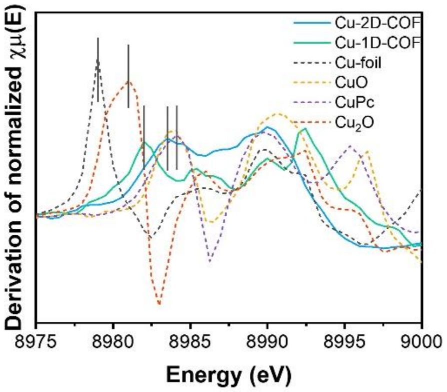

Figure S14. First-derivative curves of Cu K-edge XANES spectra of Cu-2D-COF, Cu-1D-COF, CuPc, CuO, Cu_2O and Cu foil.

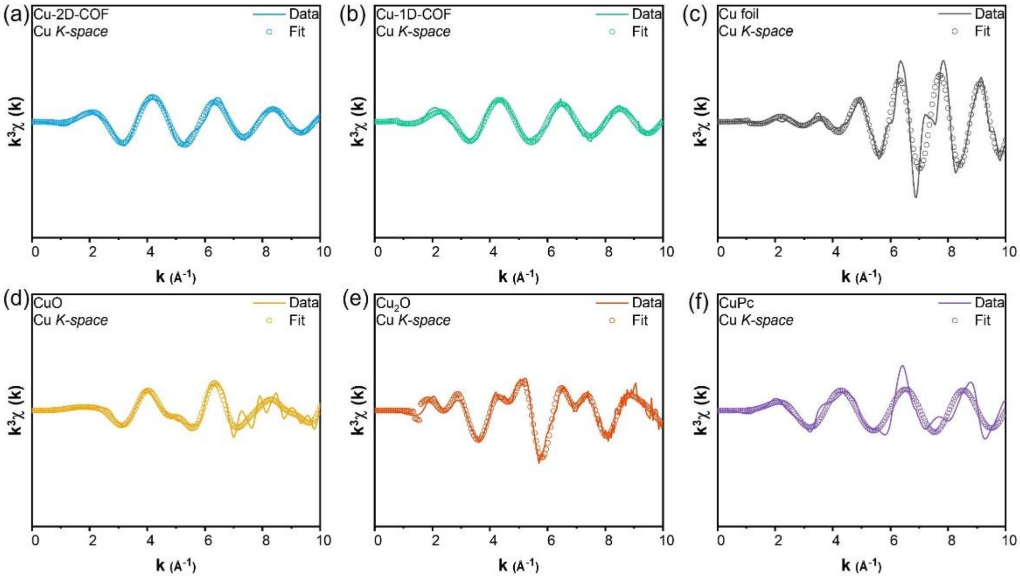

Figure S15. Fitting curves of the EXAFS of Cu atoms of (a) Cu-2D-COF, (b) Cu-1D-COF, (c) Cu foil, (d) CuO, (e) Cu_2O, and (f) CuPc in the k-space.

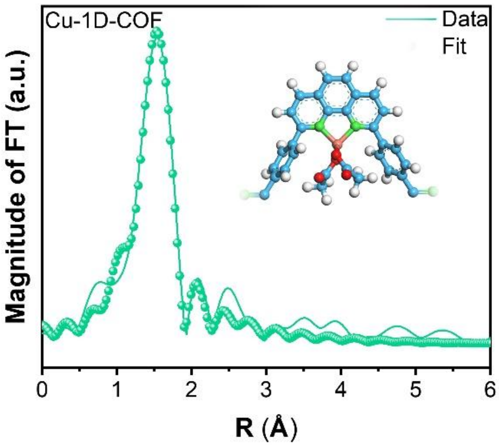

Figure S16. EXAFS fitting curve of Cu-1D-COF at R space.

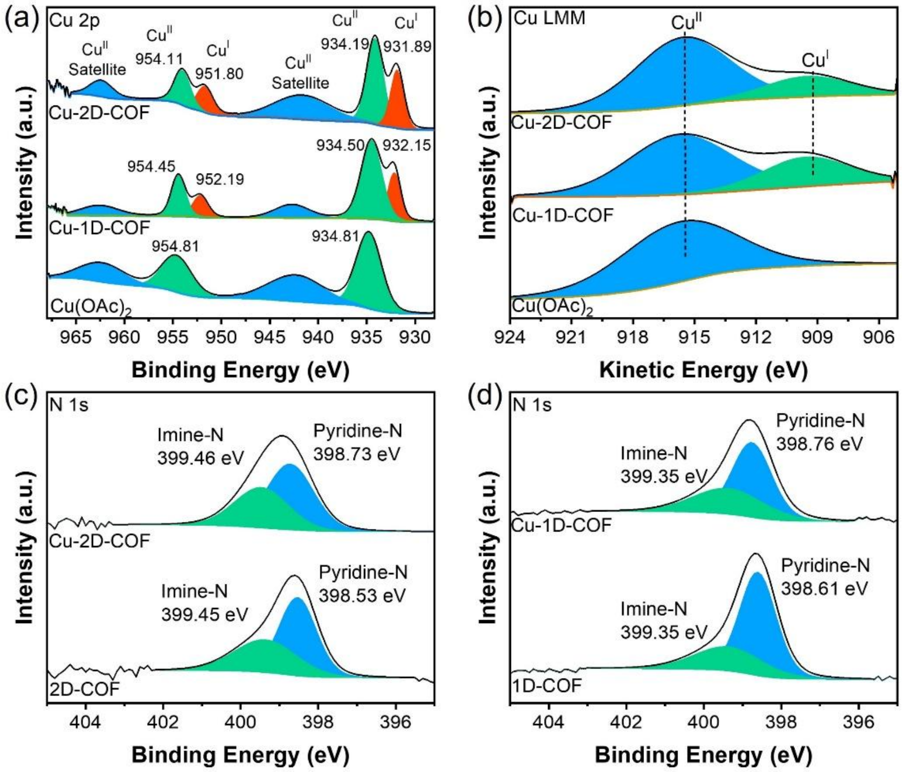

Figure S17. (a) Cu 2p XPS and (b) Cu LMM Auger spectra of Cu-2D-COF, Cu-1D-COF and $Cu(OAc)_2$. The Cu^{2+} and Cu^{+} contents were estimated from the integrated area of the corresponding curves. (c) N 1s XPS spectra of Cu-2D-COF and 2D-COF. (d) N 1s XPS spectra of Cu-1D-COF and 1D-COF.

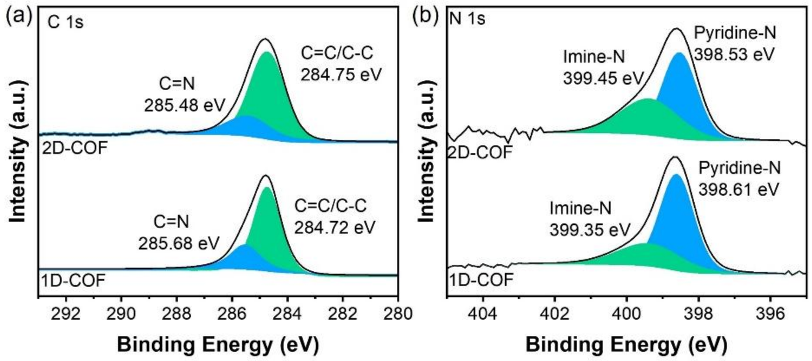

Figure S18. High-resolution (a) C 1s and (b) N 1s XPS spectra of 1D-COF and 2D-COF.

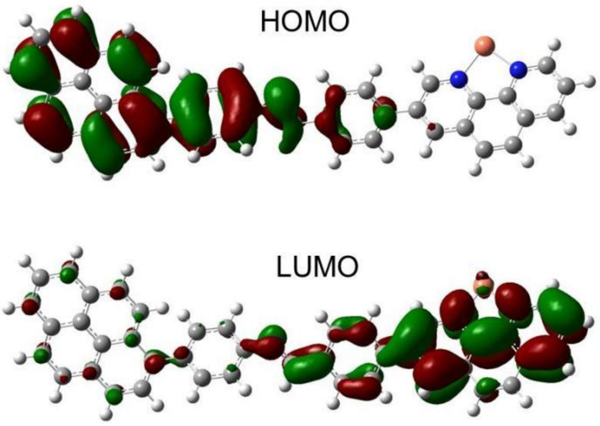

Figure S19. Molecular frontier orbitals of Cu-2D-COF (Cu^{2+}).

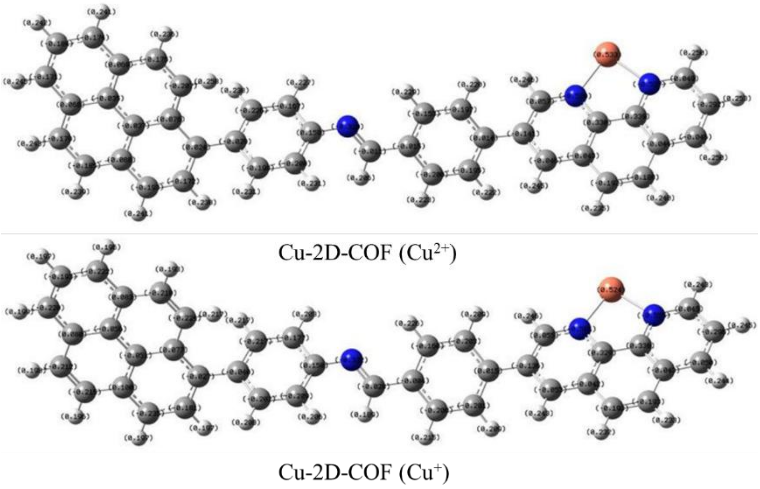

Figure S20. Mulliken charge distribution of Cu-2D-COF.

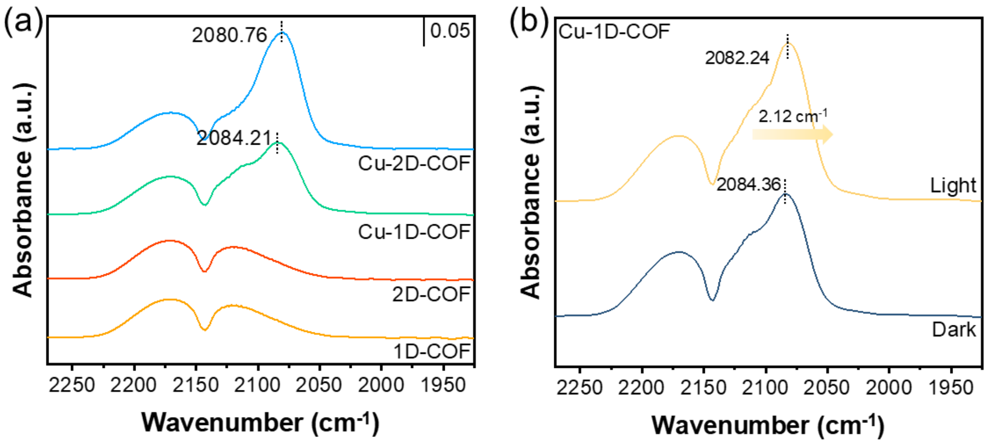

Figure S21. (a) *In-situ* DRIFTS spectra of CO adsorption on Cu-2D-COF, Cu-1D-COF, 2D-COF, and 1D-COF in the dark. (b) *In-situ* DRIFTS spectra of CO adsorption on Cu-1D-COF in the dark and under light irradiation.

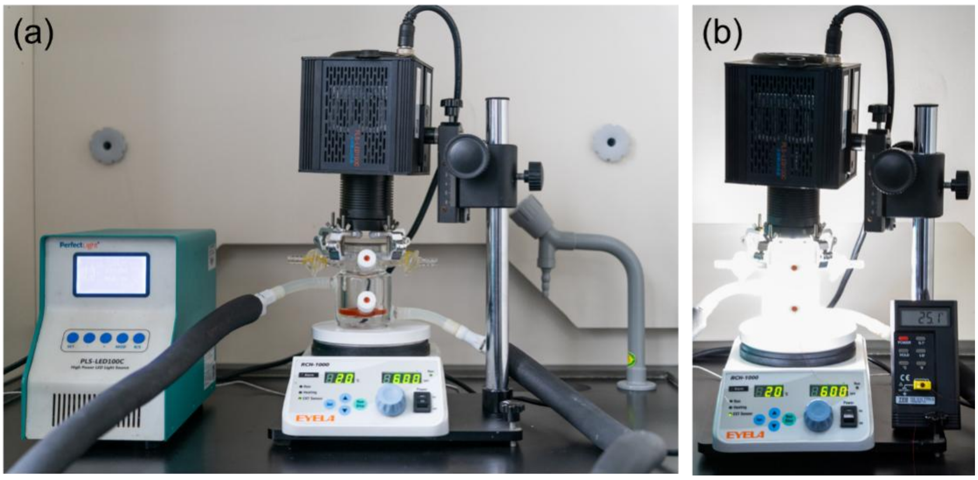

Figure S22. Images of the photocatalytic experimental facility (a) before light irradiation and (b) after light irradiation.

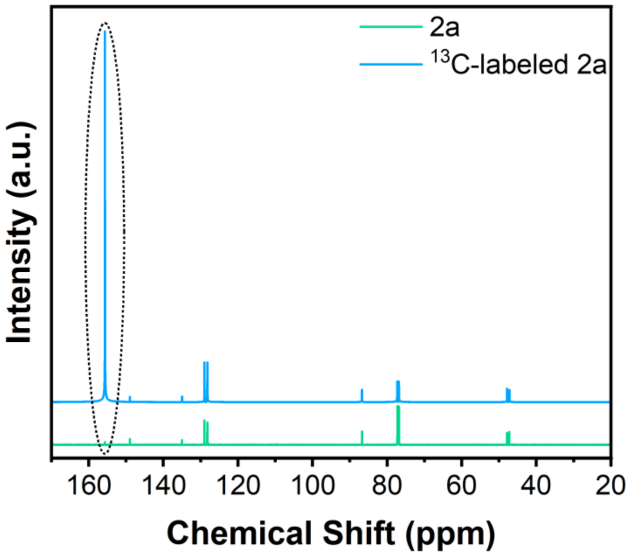

Figure S23. ^{13}C NMR spectra of ^{13}C-labeled 2a and unlabeled 2a produced from the carboxylative cyclization of **1a** using $^{13}CO_2$ as substrate.

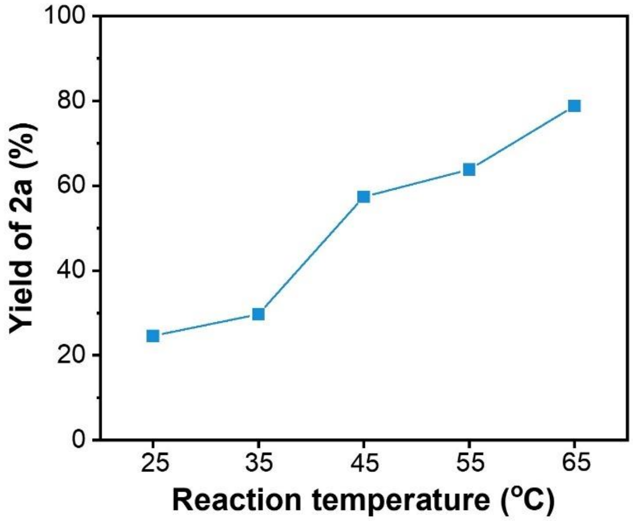

Figure S24. The catalytic activity of Cu-2D-COF with varying reaction temperature in the dark.

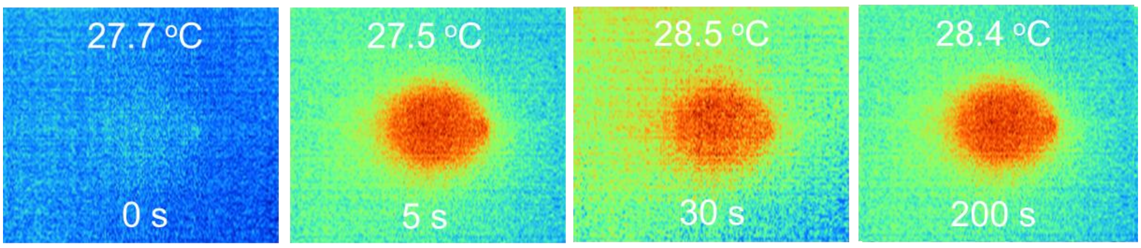

Figure S25. Photothermal images of no catalyst under different times of light irradiation.

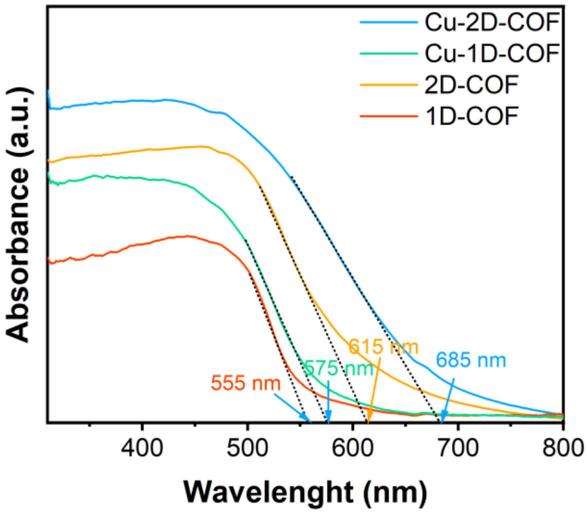

Figure S26. UV-vis absorption spectra of various samples.

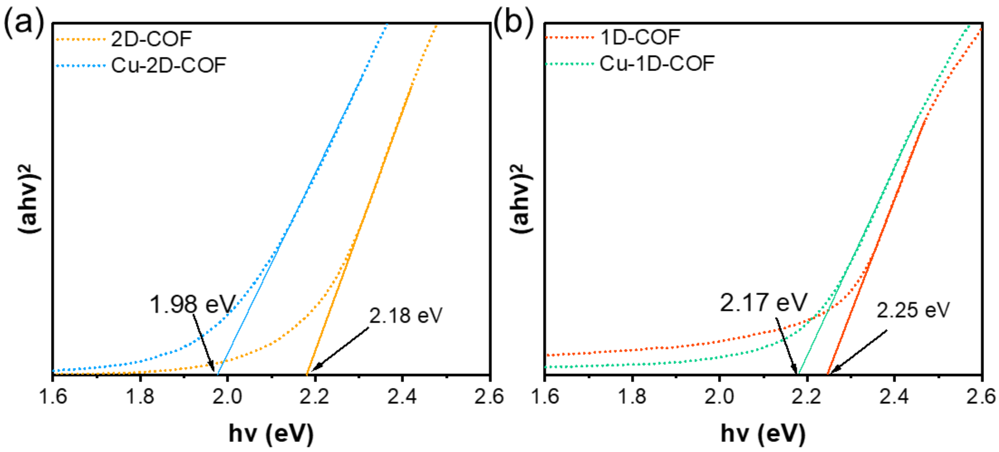

Figure S27. Tauc plots of (a) 2D-COF and Cu-2D-COF, (b) 1D-COF and Cu-1D-COF.

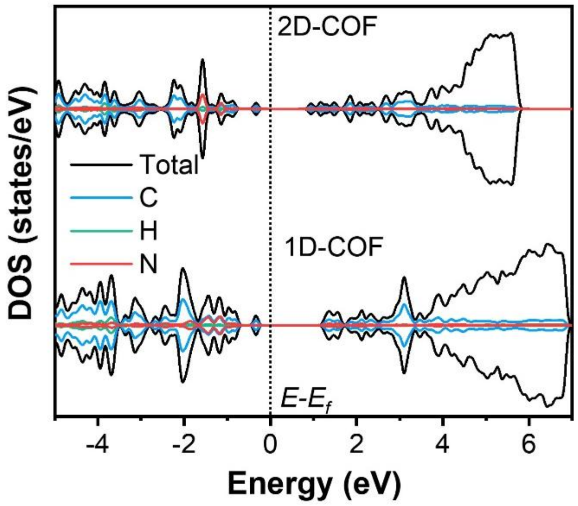

Figure S28. The DOS of 1D-COF and 2D-COF.

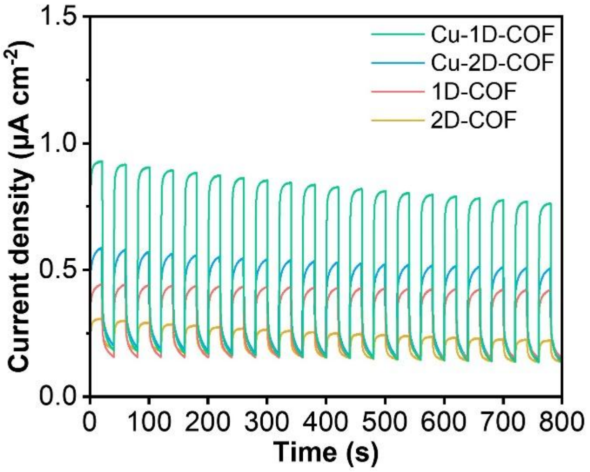

Figure S29. The photocurrent response of Cu-2D-COF, Cu-1D-COF, 2D-COF, and 1D-COF.

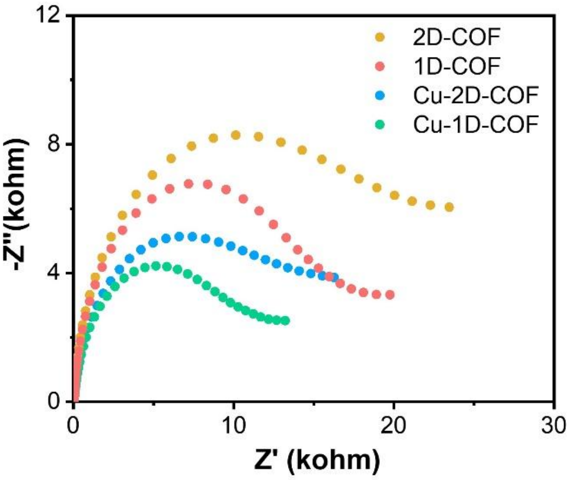

Figure S30. EIS curves of Cu-2D-COF, Cu-1D-COF, 2D-COF, and 1D-COF.

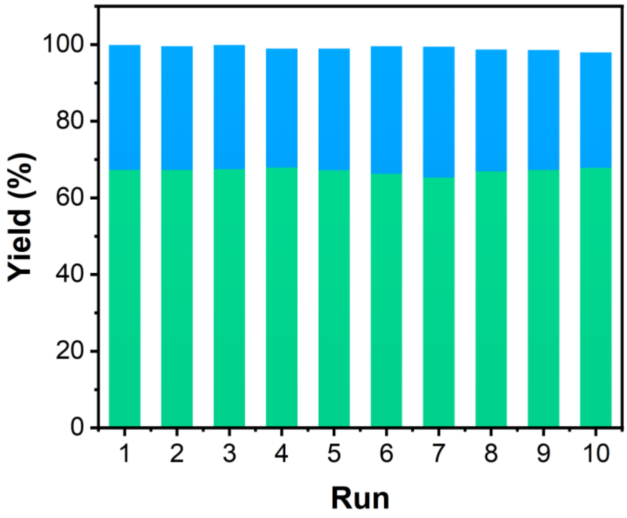

Figure S31. Recyclability tests for Cu-2D-COF in photothermal carboxylative cyclization of propargylic amines with $CO_{2.}$ (Blue represents recyclability tests under optimal conditions, while green represents recyclability tests under low conversion conditions.)

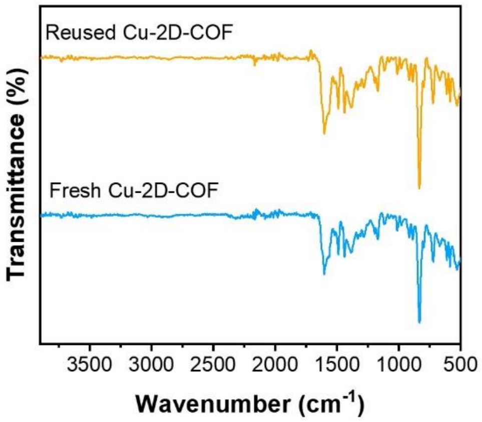

Figure S32. FT-IR spectra of fresh and reused Cu-2D-COF.

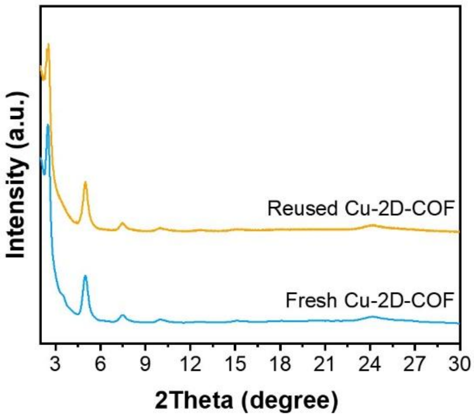

Figure S33. PXRD patterns of fresh and reused Cu-2D-COF.

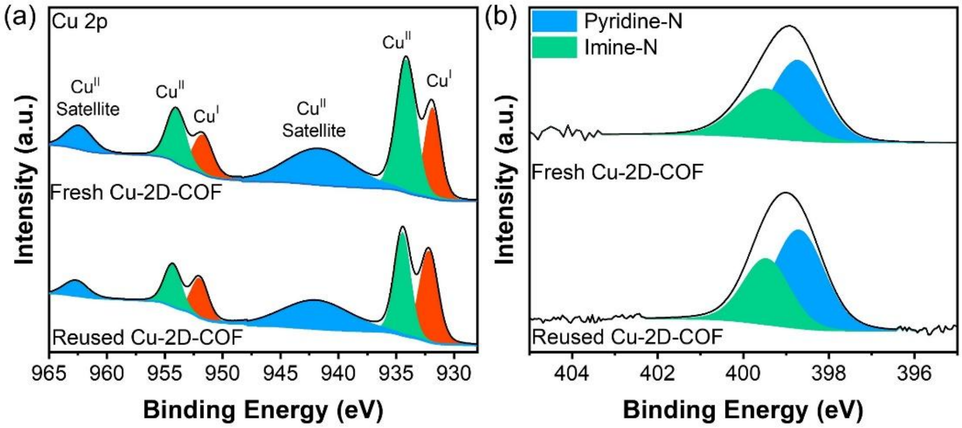

Figure S34. High-resolution (a) Cu 2p and (b) N 1s XPS spectrum of fresh and reused Cu-2D-COF.

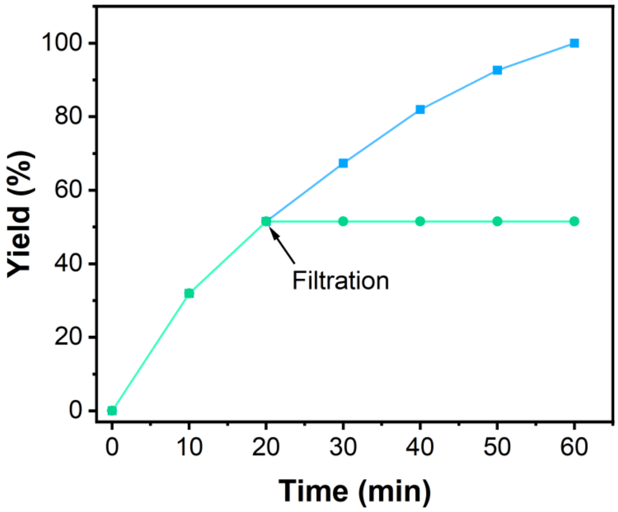

Figure S35. Hot filtration experiment using Cu-2D-COF.

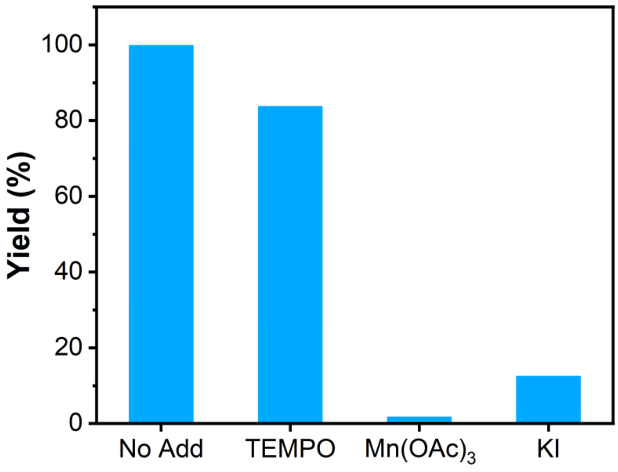

Figure S36. Yield of target product using Cu-2D-COF in the absence (pristine) or presence of different scavengers.

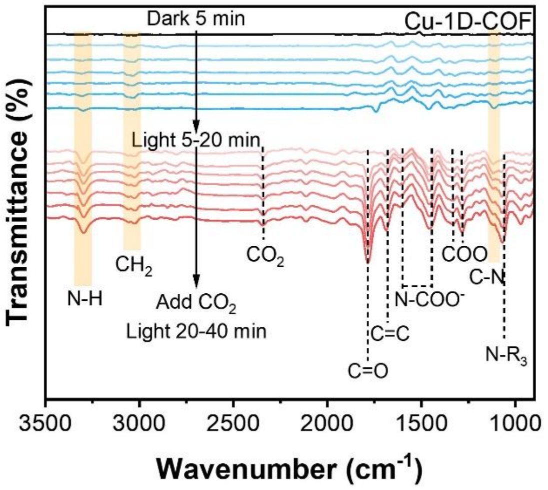

Figure S37. *In-situ* FTIR of Cu-1D-COF with **1a** in a CO_2 atmosphere in the dark and under light irradiation.

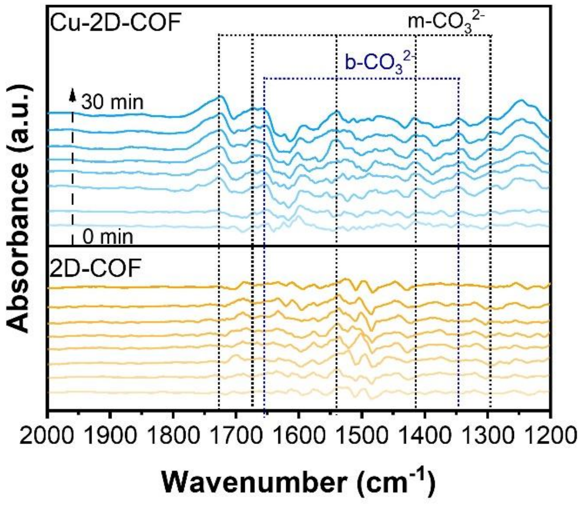

Figure S38. *In-situ* FTIR spectra of CO_2 adsorption over Cu-2D-COF and 2D-COF.

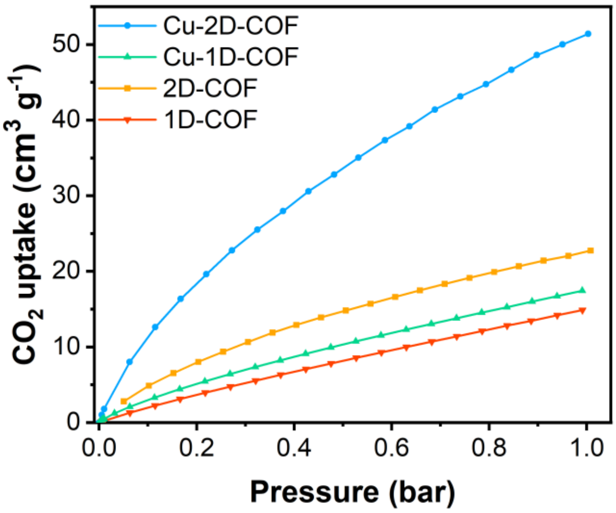

Figure S39. CO_2 sorption isotherms of various samples at 273 K.

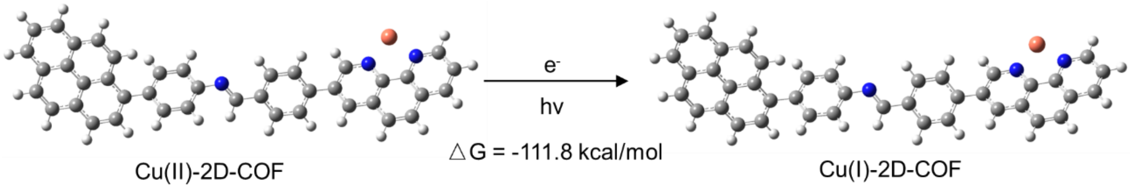

Figure S40. DFT calculations of the conversion of Cu(II) to Cu(I) under light irradiation within the 2D-COF.

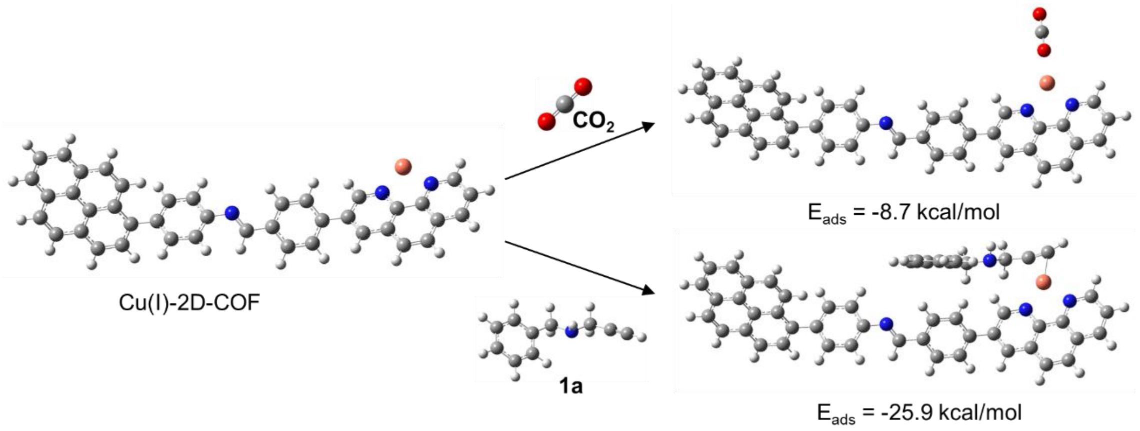

Figure S41. DFT calculations of CO_2 and 1a adsorption onto the Cu(I)-2D-COF

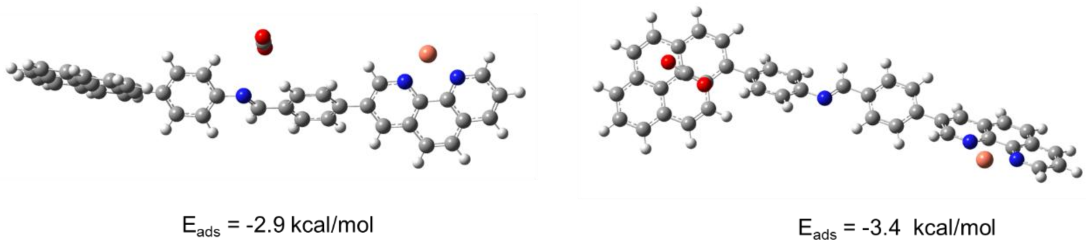

Figure S42. DFT calculations of CO_2 adsorption onto the imine and pyrene moieties of Cu(I)-2D-COF.

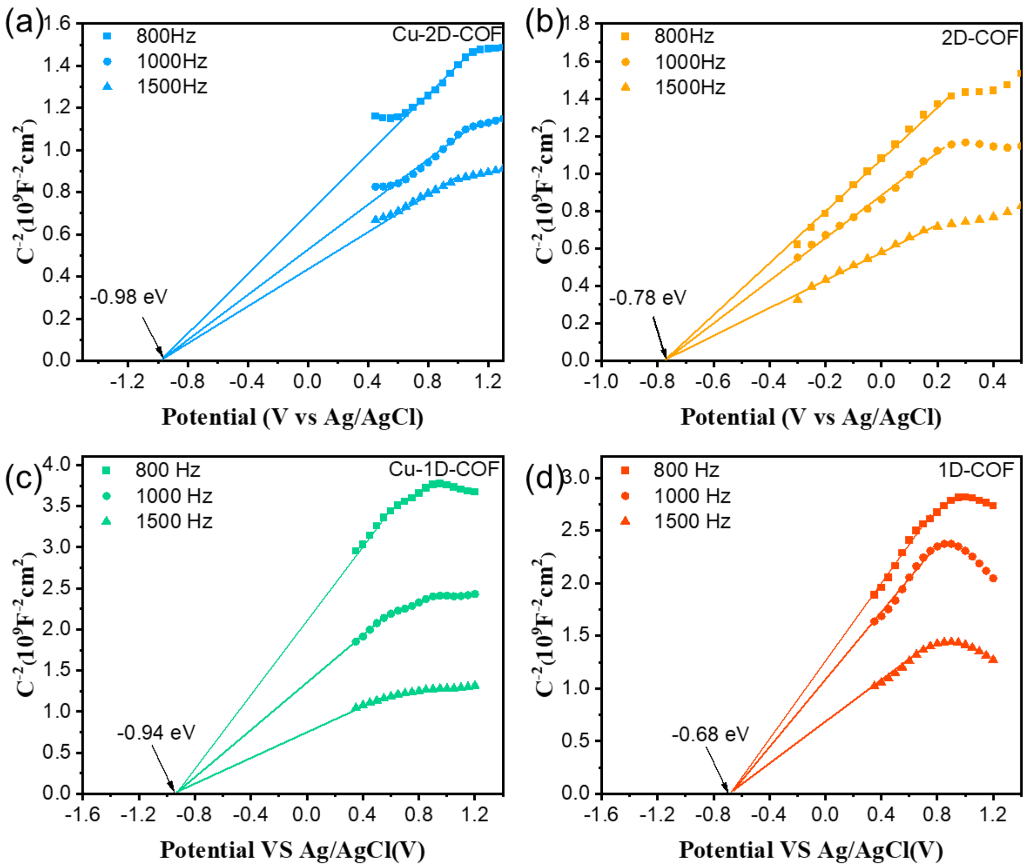

Figure S43. Mott-Schottky curves of (a) Cu-2D-COF, (b) 2D-COF, (c) Cu-1D-COF, and (d) 1D-COF.

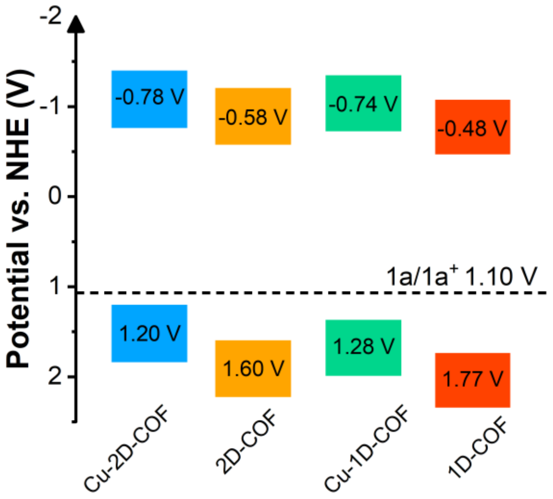

Figure S44. Experimental estimated band structure diagrams of various samples.

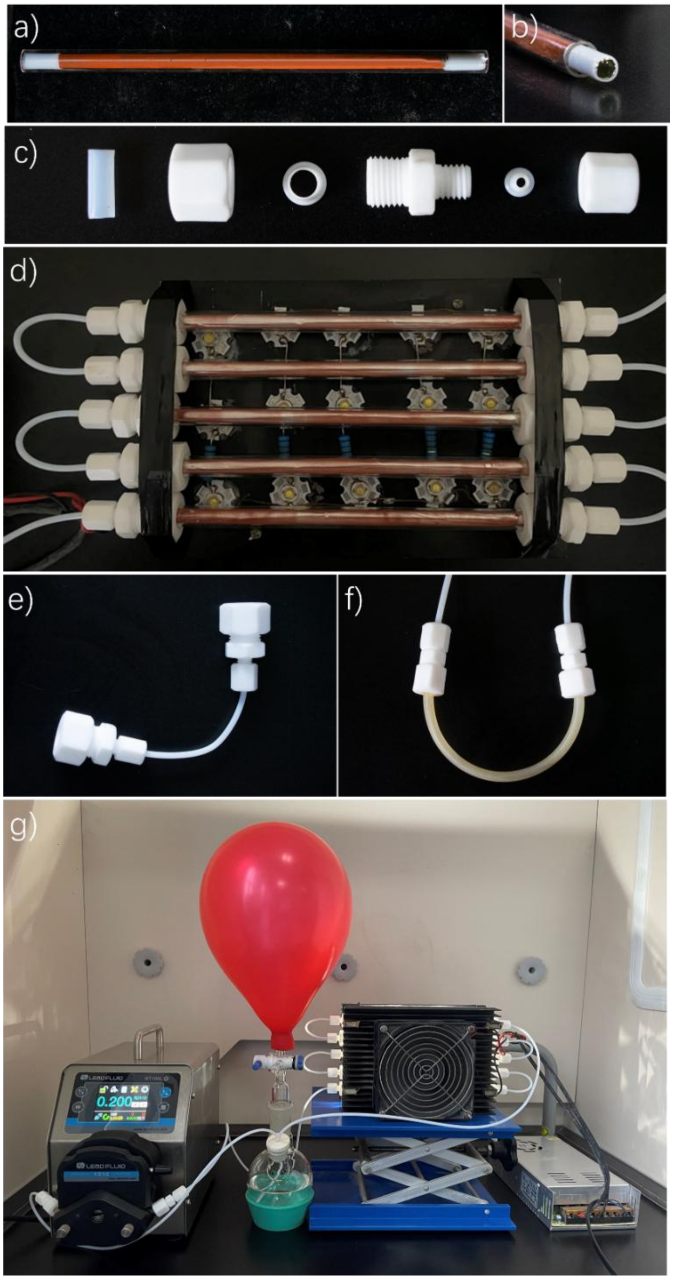

Figure S45. (a, b) Photographs of the assembled tube; (c) PTFE connector; (d) module with 5 tubes and planar LED panel. (e) Photographs of PTFE tubing and fittings. (f) peristaltic pump tube (lined with PTFE), (g) the complete tubular flow system.

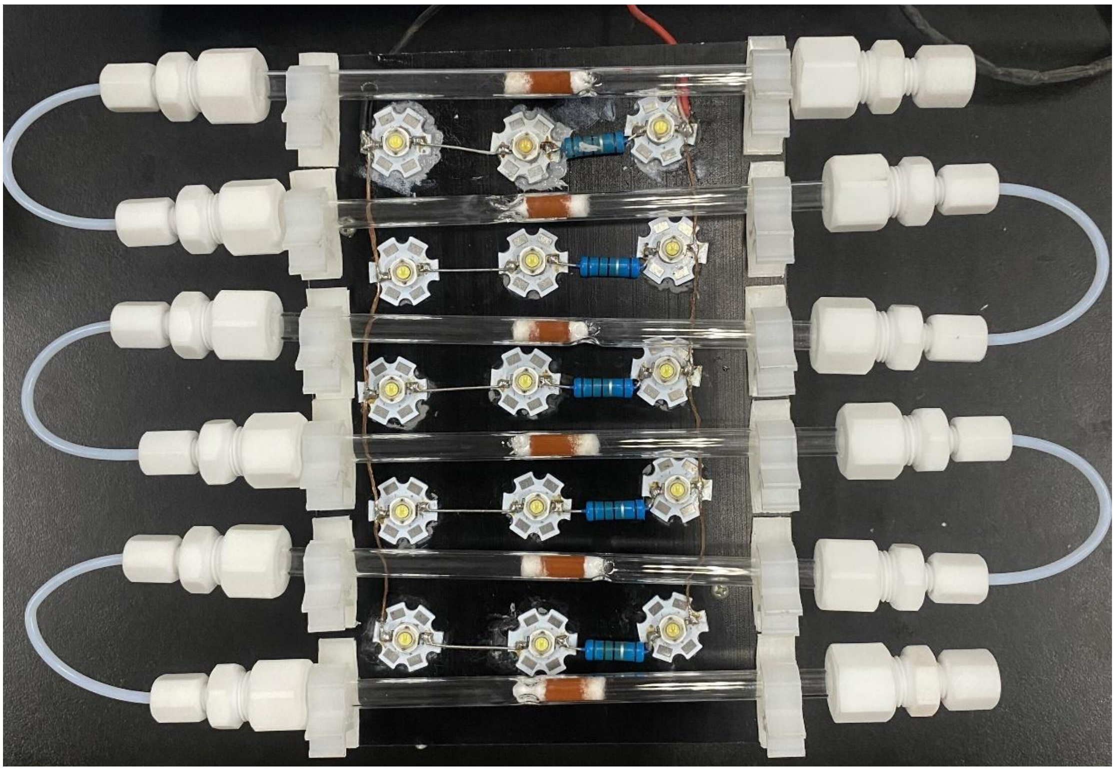

Figure S46. Photographs of the micro fixed-bed device filled with Cu-2D-COF powder.

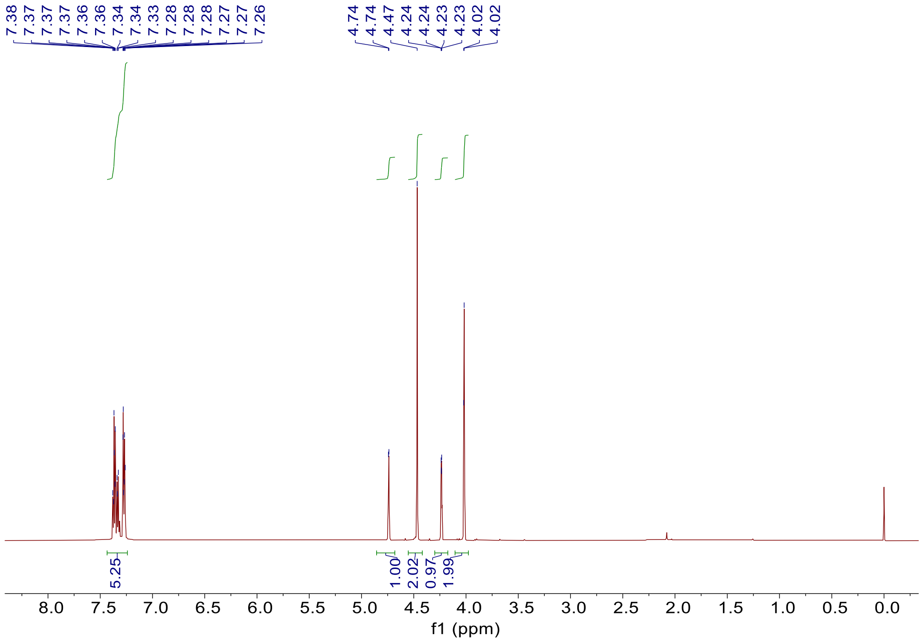

Figure S47. ^{1}H NMR spectrum of target product **2a** was obtained using the continuous-flow system.

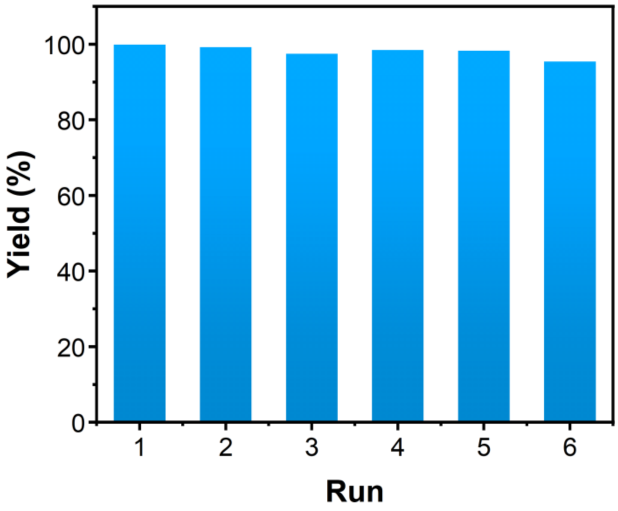

Figure S48. Cyclic tests for Cu-2D-COF photocatalyst membrane in photothermal carboxylative cyclization of propargylic amines with CO_2.

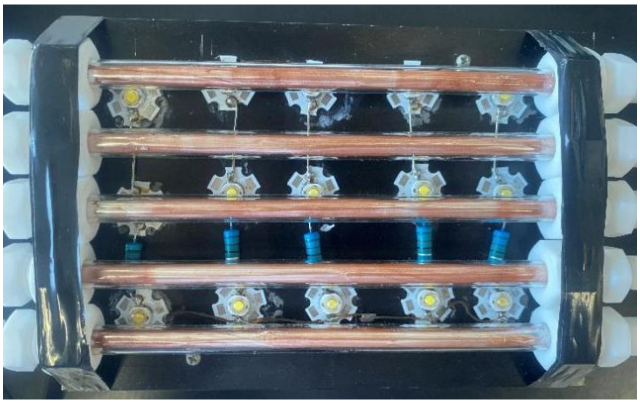

Figure S49. Cu-2D-COF photocatalyst membrane after irradiation by LED lamp for 48 h.

Section 3. Supplement tables

Table S1. Atomic coordinates of the AA-stacking mode of 2D-COF.

2D-COF space group: P1							
a = 50.0316 Å, b = 49.7033 Å, c= 3.7129 Å, α = β= γ= 90°							
Atom	x	y	z	Atom	x	y	z
H1	0.25791	0.82387	-0.05564	C155	0.54157	0.48432	0.35468
H2	0.29591	0.79957	-0.04359	C156	0.52118	0.43793	0.21366
H3	0.31936	0.75673	0.07992	C157	0.54276	0.41959	0.20483
H4	0.21546	0.82314	0.04713	C158	0.54245	0.39244	0.35593
H5	0.28478	0.6821	0.46373	C159	0.56211	0.37465	0.34467
H6	0.18418	0.74619	0.42578	C160	0.5829	0.38385	0.18739
C7	0.437	0.57109	0.07478	C161	0.58326	0.41071	0.03552
C8	0.47866	0.53083	0.2938	N162	0.60401	0.36707	0.17856
C9	0.45803	0.48493	0.32841	C163	0.39408	0.65954	0.35506
C10	0.47817	0.4382	0.19881	C164	0.8712	0.16972	0.17152
C11	0.45645	0.4201	0.17548	C165	0.86865	0.19732	0.33561
C12	0.45631	0.39279	0.32306	C166	0.84696	0.21109	0.33984
C13	0.43649	0.37529	0.30048	C167	0.82731	0.19767	0.17627
C14	0.41598	0.38494	0.13534	C168	0.83009	0.1701	0.00754
C15	0.41606	0.41193	-0.01322	C169	0.8517	0.15622	0.00754
N16	0.39466	0.36859	0.11815	C170	0.80406	0.21218	0.18306
C17	0.60779	0.65742	0.40149	C171	0.78332	0.19598	0.18213
C18	0.12691	0.17228	0.17684	C172	0.7616	0.2097	0.18117
C19	0.12891	0.20008	0.33737	C173	0.76074	0.24006	0.19502
C20	0.15019	0.21473	0.33737	N174	0.78094	0.25539	0.19842
C21	0.16996	0.20205	0.17238	C175	0.8022	0.24241	0.18961
C22	0.16778	0.17416	0.00965	H176	0.52708	0.38512	0.49154
C23	0.14658	0.15938	0.01417	H177	0.5609	0.35431	0.46982
C24	0.19269	0.21774	0.17043	H178	0.59896	0.41796	-0.09192
C25	0.21399	0.20263	0.16708	H179	0.40883	0.67202	0.45606
N26	0.23461	0.21697	0.161	H180	0.88339	0.20805	0.46712
C27	0.23571	0.24619	0.16459	H181	0.84556	0.23193	0.48049
C28	0.21493	0.26246	0.17697	H182	0.81571	0.15933	-0.13205
C29	0.19348	0.24802	0.17325	H183	0.85317	0.13504	-0.1249
H30	0.4714	0.38515	0.46582	H184	0.56404	0.4478	-0.10193
H31	0.43729	0.35486	0.42461	H185	0.55789	0.47401	0.41123
H32	0.40056	0.41955	-0.14604	C186	0.06294	0.06848	-0.16746
H33	0.5932	0.6702	0.49946	C187	0.02142	0.02787	0.10743
H34	0.11407	0.21032	0.46884	C188	0.04235	0.98431	0.24869

H35	0.15118	0.23572	0.47548	C189	0.02163	0.93982	0.39502
H36	0.18228	0.16384	-0.12917	C190	0.04294	0.92109	0.40073
H37	0.14553	0.138	-0.11415	C191	0.04242	0.89363	0.22265
H38	0.43581	0.44881	-0.13816	C192	0.06198	0.87566	0.21934
H39	0.44149	0.47487	0.37485	C193	0.08278	0.88494	0.39113
C40	0.93666	0.06779	-0.18081	C194	0.08324	0.91195	0.57339
C41	0.97846	0.02765	0.10233	N195	0.10394	0.8683	0.38007
C42	0.9578	0.98394	0.24101	C196	0.89416	0.15533	0.17739
C43	0.97879	0.93963	0.39159	C197	0.37122	0.674	0.33198
C44	0.95758	0.92074	0.39545	C198	0.36924	0.70315	0.44356
C45	0.95821	0.89318	0.21999	C199	0.34779	0.71728	0.42416
C46	0.93882	0.87495	0.21815	C200	0.32786	0.70249	0.29313
C47	0.91807	0.88406	0.38876	C201	0.32986	0.67328	0.18151
C48	0.91748	0.91122	0.56703	C202	0.35133	0.65916	0.20063
N49	0.89716	0.86696	0.38346	C203	0.30507	0.71748	0.27381
C50	0.10431	0.15714	0.18482	C204	0.28438	0.70374	0.37037
C51	0.63091	0.67128	0.38753	N205	0.26359	0.71772	0.35653
C52	0.63338	0.70028	0.50404	C206	0.26188	0.74516	0.24917
C53	0.65513	0.71377	0.4953	C207	0.28215	0.75986	0.14796
C54	0.67486	0.69848	0.37022	C208	0.30372	0.7458	0.16118
C55	0.67235	0.66945	0.2524	H209	0.02697	0.8863	0.07649
C56	0.6506	0.65597	0.26089	H210	0.0608	0.85519	0.07069
C57	0.698	0.71271	0.36352	H211	0.09898	0.91929	0.71134
C58	0.71823	0.69804	0.46439	H212	0.90849	0.16661	0.3106
C59	0.74001	0.71156	0.45508	H213	0.3843	0.71498	0.54784
C60	0.74129	0.74011	0.34452	H214	0.3467	0.73967	0.5173
N61	0.72145	0.75392	0.24893	H215	0.3149	0.66145	0.07421
C62	0.70017	0.7411	0.25622	H216	0.35233	0.63668	0.11106
H63	0.97363	0.88593	0.0757	H217	0.06424	0.9495	0.74295
H64	0.94009	0.85438	0.07236	H218	0.05928	0.97464	0.26897
H65	0.90179	0.91842	0.70434	H219	0.31393	0.24397	0.09163
H66	0.0899	0.16804	0.31793	H220	0.21441	0.17943	0.17435
H67	0.61848	0.71246	0.60444	H221	0.28097	0.32738	0.22158
H68	0.6566	0.73604	0.59255	H222	0.17753	0.26048	0.16887
H69	0.68716	0.65726	0.14897	C223	0.43617	0.42892	-0.00264
H70	0.64922	0.63361	0.16718	C224	0.47835	0.46868	0.25977
H71	0.93623	0.94913	0.73258	C225	0.45817	0.51494	0.34409
H72	0.94093	0.97415	0.25898	C226	0.47879	0.56126	0.26718
C73	0.25886	0.80199	0.0304	C227	0.45728	0.5797	0.26493
C74	0.28049	0.78816	0.03726	C228	0.45739	0.60713	0.44434
H75	0.74129	0.17037	0.15407	C229	0.43781	0.62504	0.43815
H76	0.70346	0.19502	0.14563	C230	0.41735	0.61567	0.2576

H77	0.68096	0.23981	0.15364	C231	0.41713	0.58847	0.08028
H78	0.78395	0.17272	0.18566	N232	0.39639	0.63263	0.24995
H79	0.71749	0.32093	0.28315	C233	0.60669	0.34118	0.288
H80	0.81756	0.25583	0.1827	C234	0.12969	0.82805	0.25315
C81	0.56333	0.42801	0.03537	C235	0.13198	0.79947	0.11877
C82	0.5212	0.46838	0.27403	C236	0.15378	0.78598	0.11344
C83	0.54174	0.51432	0.36816	C237	0.17373	0.80087	0.24132
C84	0.52179	0.5609	0.27824	C238	0.17142	0.82951	0.37535
C85	0.54354	0.57891	0.28523	C239	0.14962	0.84296	0.38191
C86	0.54364	0.60634	0.46461	C240	0.19685	0.78656	0.23693
C87	0.56346	0.62387	0.466	C241	0.21688	0.80128	0.13276
C88	0.58392	0.6141	0.29296	C242	0.23866	0.78765	0.13379
C89	0.58394	0.58691	0.11581	C243	0.24014	0.75903	0.24155
N90	0.60512	0.63061	0.29336	N244	0.22049	0.74518	0.34039
C91	0.39167	0.34245	0.21704	C245	0.1992	0.75805	0.33947
C92	0.87203	0.82578	0.27426	H246	0.47251	0.61457	0.59768
C93	0.87017	0.79682	0.15318	H247	0.4387	0.64559	0.5857
C94	0.84875	0.78255	0.1621	H248	0.40164	0.58108	-0.0641
C95	0.82873	0.79702	0.2914	H249	0.59229	0.32959	0.39731
C96	0.83059	0.82608	0.41073	H250	0.11691	0.78762	0.01558
C97	0.85202	0.84034	0.40249	H251	0.15511	0.76403	0.00375
C98	0.80606	0.78179	0.30523	H252	0.18643	0.84134	0.48085
C99	0.78523	0.79545	0.20964	H253	0.14838	0.86499	0.48909
N100	0.76449	0.78142	0.22489	H254	0.43647	0.55108	-0.08322
C101	0.76299	0.75396	0.33229	H255	0.4417	0.52528	0.39951
C102	0.78344	0.73929	0.43065	C256	0.93714	0.92898	0.5789
C103	0.80494	0.75342	0.41605	C257	0.97863	0.96897	0.29821
H104	0.52847	0.61409	0.61153	C258	0.95771	0.01258	0.15646
H105	0.56272	0.64445	0.61283	C259	0.97845	0.05691	0.00669
H106	0.59945	0.57919	-0.02218	C260	0.95724	0.07581	0.00439
H107	0.40598	0.33022	0.32132	C261	0.95808	0.10321	0.18362
H108	0.8853	0.78523	0.05063	C262	0.93872	0.12149	0.18882
H109	0.84777	0.76027	0.06343	C263	0.91778	0.11257	0.01839
H110	0.81555	0.83764	0.51704	C264	0.91703	0.08563	-0.16585
H111	0.85292	0.86267	0.49881	N265	0.89684	0.12965	0.02889
H112	0.56429	0.54983	-0.05345	C266	0.10651	0.84193	0.25387
H113	0.55824	0.52442	0.43144	C267	0.62961	0.32671	0.26744
C114	0.06341	0.92944	0.5869	C268	0.63187	0.299	0.39408
C115	0.02161	0.96916	0.30193	C269	0.65337	0.28487	0.38266
C116	0.04226	0.01298	0.16574	C270	0.67311	0.29793	0.23789
C117	0.02124	0.05713	0.0121	C271	0.67072	0.32576	0.10951
C118	0.04231	0.07625	0.01414	C272	0.64925	0.34006	0.12623

C119	0.04124	0.10368	0.1917	C273	0.69597	0.28253	0.2212
C120	0.06034	0.12234	0.19687	C274	0.71722	0.29771	0.24699
C121	0.08131	0.11376	0.02878	N275	0.73791	0.2834	0.23468
C122	0.08233	0.08672	-0.15253	C276	0.73907	0.25417	0.20298
N123	0.10196	0.13135	0.0376	C277	0.7183	0.2379	0.18158
C124	0.89489	0.84034	0.26475	C278	0.69686	0.25229	0.18355
C125	0.36845	0.32872	0.19379	H279	0.97365	0.1103	0.32805
C126	0.36558	0.3011	0.3203	H280	0.94026	0.14199	0.33575
C127	0.34369	0.28789	0.31022	H281	0.9012	0.07864	-0.3042
C128	0.32415	0.30184	0.1678	H282	0.09183	0.82959	0.14787
C129	0.32721	0.32945	0.03663	H283	0.61704	0.28839	0.50793
C130	0.34906	0.34282	0.0519	H284	0.65453	0.26396	0.49572
C131	0.30077	0.28772	0.15945	H285	0.68526	0.33632	-0.01209
C132	0.28015	0.30412	0.18881	H286	0.64798	0.36137	0.02366
C133	0.25833	0.29055	0.18313	H287	0.93563	0.0478	-0.33846
C134	0.25735	0.26021	0.1596	H288	0.9408	0.02227	0.13407
N135	0.27742	0.24472	0.12999	C289	0.50002	0.51567	0.28576
C136	0.2987	0.25753	0.1254	C290	0.49967	0.4239	0.15741
H137	0.02564	0.11057	0.33382	H291	0.49965	0.40107	0.08325
H138	0.05856	0.1429	0.34116	C292	0.49985	0.48351	0.26846
H139	0.09816	0.07998	-0.28934	C293	0.5004	0.57521	0.23995
H140	0.90964	0.82818	0.16099	H294	0.50058	0.59797	0.19103
H141	0.38022	0.28993	0.43454	C295	-0.00001	0.01304	0.15077
H142	0.34206	0.26705	0.42369	C296	0.00026	0.92635	0.45314
H143	0.31287	0.34062	-0.0846	H297	0.00033	0.90433	0.53602
H144	0.3508	0.36406	-0.05123	C298	0.00008	0.98376	0.25429
H145	0.06415	0.04843	-0.32332	C299	-0.00019	0.07028	-0.05393
H146	0.05911	0.02286	0.14767	H300	-0.00026	0.09224	-0.13879
C147	0.74085	0.1937	0.16578	C301	0.7604	0.6972	0.55376
C148	0.71931	0.20773	0.16106	C302	0.782	0.71098	0.54142
H149	0.685	0.75294	0.17217	C303	0.23762	0.30662	0.20095
H150	0.78545	0.81714	0.11758	C304	0.21601	0.29264	0.19349
H151	0.71699	0.67632	0.55489	H305	0.75961	0.67533	0.64046
H152	0.8207	0.74254	0.49656	H306	0.79757	0.69956	0.6188
C153	0.56386	0.5699	0.10308	H307	0.23813	0.32995	0.21755
C154	0.52152	0.53049	0.3057	H308	0.20019	0.3054	0.20433

Table S2. Atomic coordinates of the AA-stacking mode of 1D-COF.

1D-COF space group: P1							
a =41.4203 Å, b = 24.5156 Å, c= 3.9021 Å, α =88.6838° β=89.8567° γ= 91.5895°							
Atom	x	y	z	Atom	x	y	z
C1	1.54351	0.43368	0.51024	C155	0.98645	0.15509	0.46163
C2	1.54308	0.48904	0.55532	C156	0.98667	0.10185	0.54469
C3	1.46692	0.52514	0.3977	C157	1.01418	0.076	0.57477
C4	1.46389	0.40314	0.38355	C158	1.04126	0.10616	0.55212
C5	1.44447	0.41931	0.30359	C159	1.04107	0.16001	0.47253
C6	1.41718	0.39165	0.28124	C160	1.01365	0.18196	0.42013
C7	1.4097	0.34609	0.32507	C161	1.06865	0.0798	0.62066
C8	1.42938	0.32955	0.40017	C162	0.96036	0.07385	0.60284
C9	1.45616	0.35796	0.43075	C163	1.06832	0.19555	0.44079
N10	1.38426	0.31209	0.28261	C164	0.95861	0.18456	0.4153
C11	1.38129	0.26062	0.33083	C165	1.09497	0.17582	0.36768
C12	1.35332	0.2297	0.29623	C166	1.11982	0.21064	0.33711
C13	1.32837	0.25525	0.21946	C167	1.11773	0.2668	0.36319
C14	1.30101	0.22735	0.20914	C168	1.09087	0.28678	0.42594
C15	1.29761	0.17403	0.28625	C169	1.06678	0.25164	0.46989
C16	1.32322	0.14694	0.34862	C170	0.95682	0.2397	0.45553
C17	1.35087	0.17448	0.35188	C171	0.93096	0.2679	0.41673
C18	1.26768	0.14762	0.31592	C172	0.90561	0.24076	0.34805
C19	1.24161	0.17783	0.32193	C173	0.90728	0.18599	0.30692
C20	1.21401	0.15155	0.35237	C174	0.93408	0.15887	0.32983
C21	1.21252	0.09524	0.38527	N175	1.14254	0.30431	0.33645
C22	1.23923	0.06639	0.38872	C176	1.16634	0.2959	0.23845
N23	1.26567	0.09317	0.35308	C177	1.1918	0.33472	0.23658
C24	1.18523	0.0677	0.42147	C178	1.21923	0.31782	0.1705
H25	1.44664	0.50753	0.34307	C179	1.24472	0.3505	0.18783
H26	1.4022	0.40432	0.22076	C180	1.24338	0.4017	0.26839
H27	1.42385	0.29504	0.43831	C181	1.21528	0.42066	0.31916
H28	1.47087	0.34483	0.49204	C182	1.18972	0.38713	0.30585
H29	1.33	0.29743	0.17409	C183	1.27103	0.43477	0.30203
H30	1.28243	0.24822	0.14677	C184	1.2991	0.41067	0.30836
H31	1.37008	0.15342	0.40608	C185	1.3247	0.44211	0.33612
H32	1.24254	0.22141	0.31122	C186	1.32244	0.49769	0.36043
H33	1.19408	0.17517	0.3562	C187	1.29389	0.52087	0.3638
H34	1.16472	0.08984	0.42092	N188	1.26923	0.48885	0.33345
H35	1.45039	0.45349	0.2605	C189	1.34818	0.5302	0.37963
H36	1.39944	0.2395	0.39939	C190	1.34592	0.58607	0.39547

H37	1.32158	0.10519	0.40321	C191	1.31789	0.60967	0.399
C38	1.49088	0.43647	0.42568	C192	1.29159	0.57696	0.39108
C39	1.49149	0.49343	0.44545	C193	1.31556	0.66585	0.40334
C40	1.56741	0.51443	0.64376	C194	1.28761	0.68858	0.41184
C41	1.57184	0.40418	0.49876	C195	1.26184	0.65499	0.42753
C42	1.59606	0.42829	0.39766	N196	1.26451	0.60044	0.40885
C43	1.62259	0.40076	0.38091	C197	1.23213	0.67818	0.46792
C44	1.62496	0.34788	0.45551	C198	1.2056	0.648	0.43886
C45	1.6008	0.32346	0.55746	C199	1.17781	0.67054	0.47414
C46	1.57439	0.35152	0.57874	C200	1.17613	0.72387	0.53576
N47	1.65206	0.32045	0.42204	C201	1.20243	0.75313	0.57071
C48	1.65527	0.26818	0.43232	C202	1.22997	0.73017	0.54257
C49	1.68426	0.24465	0.39294	N203	0.87748	0.26658	0.33251
C50	1.70878	0.27718	0.33086	C204	1.14727	0.75005	0.55759
C51	1.7364	0.25415	0.29978	C205	0.87408	0.31865	0.31206
C52	1.7401	0.19782	0.33271	C206	0.84453	0.34201	0.33475
C53	1.71512	0.16553	0.3906	C207	0.84235	0.39803	0.35406
C54	1.68762	0.18861	0.42054	C208	0.81476	0.42075	0.38759
C55	1.7695	0.17159	0.31898	C209	0.78862	0.38795	0.40105
C56	1.79604	0.19933	0.26455	C210	0.79111	0.3314	0.38397
C57	1.8232	0.17291	0.27887	C211	0.8188	0.30877	0.35136
C58	1.82434	0.11874	0.34732	C212	0.75961	0.41387	0.43851
C59	1.79756	0.09005	0.39498	N213	0.7597	0.46733	0.48106
N60	1.77128	0.11558	0.37977	C214	0.73449	0.49387	0.52337
C61	1.85167	0.09309	0.37221	C215	0.70678	0.46531	0.52599
H62	1.58618	0.49113	0.69349	C216	0.70588	0.41069	0.4793
H63	1.64126	0.42008	0.30559	C217	0.73213	0.38505	0.43388
H64	1.60231	0.28319	0.62199	C218	0.73568	0.54943	0.56283
H65	1.55602	0.33241	0.65831	C219	0.70908	0.57583	0.60694
H66	1.70641	0.32053	0.30655	C220	0.68187	0.54664	0.61517
H67	1.75454	0.28086	0.25242	C221	0.6807	0.49167	0.57375
H68	1.66898	0.16278	0.46783	N222	0.76202	0.57841	0.55456
H69	1.79628	0.24104	0.21297	C223	0.7636	0.63265	0.58538
H70	1.8435	0.19531	0.23929	C224	0.73762	0.65952	0.63234
H71	1.8723	0.11484	0.33574	C225	0.71035	0.63123	0.6423
H72	1.59435	0.46844	0.331	C226	0.79262	0.66264	0.57105
H73	1.63661	0.24081	0.4651	C227	0.81871	0.63786	0.63423
H74	1.71703	0.1221	0.41628	C228	0.84571	0.66745	0.63603
C75	1.51755	0.51959	0.51401	C229	0.8474	0.72144	0.56469
C76	1.51707	0.40763	0.45399	C230	0.82163	0.74542	0.49231
H77	1.51703	0.36472	0.42574	C231	0.79448	0.71636	0.49558
C78	1.49271	0.6631	0.53793	C232	0.87615	0.75183	0.56819

C79	1.49272	0.60718	0.49794	N233	0.87844	0.80287	0.52844
C80	1.56785	0.5699	0.67259	C234	0.90569	0.83436	0.51814
C81	1.57534	0.69195	0.6177	C235	0.92811	0.82195	0.56548
C82	1.60165	0.67302	0.53527	C236	0.95488	0.85212	0.55977
C83	1.62794	0.70488	0.53025	C237	0.95952	0.89575	0.51038
C84	1.62771	0.75869	0.58332	C238	0.93705	0.90848	0.46498
C85	1.60134	0.77888	0.65558	C239	0.91023	0.87826	0.4725
C86	1.57622	0.74512	0.68437	C240	0.98699	0.93011	0.53264
N87	1.65461	0.79115	0.56326	C241	1.01402	0.90355	0.54892
C88	1.65647	0.84394	0.5466	C242	1.04146	0.93227	0.54181
C89	1.68589	0.87219	0.53054	C243	1.04173	0.98987	0.51568
C90	1.71212	0.84324	0.55365	C244	1.01442	1.01676	0.48754
C91	1.73998	0.86933	0.53227	C245	0.98704	0.9872	0.50801
C92	1.74238	0.92541	0.48614	C246	1.06817	0.89794	0.54141
C93	1.71586	0.95457	0.46705	C247	1.07007	0.8495	0.58726
C94	1.68794	0.92827	0.48962	C248	1.09566	0.818	0.57834
C95	1.77191	0.95377	0.44944	C249	1.11994	0.83501	0.52869
C96	1.79929	0.92916	0.49022	C250	1.11833	0.88353	0.48805
C97	1.82615	0.95658	0.44469	C251	1.09245	0.91402	0.48754
C98	1.82591	1.00893	0.36074	N252	1.14539	0.80186	0.51756
C99	1.79828	1.03351	0.32854	C253	0.96042	1.01574	0.50929
N100	1.77248	1.00531	0.37207	C254	1.0683	1.0213	0.52452
C101	1.85247	1.03719	0.3108	H255	1.01333	0.22137	0.34684
H102	1.58663	0.58721	0.747	H256	1.08899	0.10309	0.64179
H103	1.64819	0.68924	0.47254	H257	0.93936	0.09404	0.61163
H104	1.60009	0.82058	0.6913	H258	1.09658	0.13369	0.33172
H105	1.55735	0.76173	0.75481	H259	1.14025	0.1934	0.28964
H106	1.71085	0.80015	0.58718	H260	1.08889	0.32985	0.44408
H107	1.75926	0.84479	0.54936	H261	1.04683	0.26864	0.52741
H108	1.66797	0.95148	0.47153	H262	0.97527	0.26092	0.52066
H109	1.80032	0.88947	0.55867	H263	0.93046	0.31003	0.451
H110	1.847	0.93702	0.47568	H264	0.88793	0.16505	0.25183
H111	1.87376	1.01844	0.33442	H265	0.93539	0.1174	0.28577
H112	1.60198	0.63438	0.46857	H266	1.1687	0.25894	0.16325
H113	1.63662	0.86781	0.53795	H267	1.22092	0.27926	0.1037
H114	1.71673	0.99773	0.43185	H268	1.26534	0.33608	0.13246
C115	1.54627	0.66	0.61051	H269	1.21343	0.46041	0.37756
C116	1.5445	0.60243	0.60742	H270	1.16849	0.40136	0.35378
C117	1.46818	0.5811	0.41382	H271	1.30148	0.36752	0.29833
C118	1.4651	0.69602	0.52968	H272	1.34615	0.42289	0.33941
C119	1.43736	0.67338	0.58972	H273	1.37002	0.51249	0.37676
C120	1.41122	0.70127	0.55314	H274	1.36603	0.61106	0.40183

C121	1.41234	0.75459	0.47913	H275	1.33533	0.69204	0.39793
C122	1.44021	0.78032	0.44579	H276	1.28642	0.73217	0.40767
C123	1.46621	0.75078	0.4644	H277	1.20646	0.60737	0.38648
N124	1.38484	0.78203	0.4537	H278	1.1576	0.64742	0.44657
C125	1.38101	0.82915	0.36933	H279	1.20155	0.79163	0.61972
C126	1.35145	0.85342	0.36835	H280	1.24941	0.75323	0.58306
C127	1.32663	0.82492	0.44648	H281	1.12763	0.72516	0.592
C128	1.29865	0.84817	0.44373	H282	0.8931	0.34614	0.29284
C129	1.29487	0.90081	0.36535	H283	0.86204	0.42432	0.34484
C130	1.32022	0.92999	0.29532	H284	0.81377	0.46427	0.40211
C131	1.34808	0.90633	0.29437	H285	0.77309	0.30556	0.39889
C132	1.2652	0.9266	0.35677	H286	0.82031	0.26519	0.34151
C133	1.2381	0.89762	0.39131	H287	0.68497	0.38782	0.47869
C134	1.21114	0.92456	0.37946	H288	0.73061	0.34315	0.39637
C135	1.21105	0.98023	0.33229	H289	0.66162	0.56655	0.6525
C136	1.2385	1.00781	0.29525	H290	0.65948	0.46979	0.57815
N137	1.2643	0.98043	0.30895	H291	0.73868	0.70177	0.66639
C138	1.18445	1.00882	0.32349	H292	0.69043	0.65228	0.68014
H139	1.44969	0.60365	0.35753	H293	0.81791	0.59585	0.68551
H140	1.38984	0.68203	0.58655	H294	0.86529	0.6481	0.69324
H141	1.44206	0.82262	0.40022	H295	0.82263	0.78662	0.43304
H142	1.48701	0.77099	0.42341	H296	0.77503	0.73544	0.4356
H143	1.32902	0.78483	0.51214	H297	0.89533	0.73105	0.6237
H144	1.2804	0.82558	0.50974	H298	0.92466	0.78965	0.61258
H145	1.367	0.92954	0.23638	H299	0.97185	0.84209	0.59828
H146	1.23731	0.85451	0.4231	H300	0.94039	0.94189	0.4245
H147	1.19043	0.90191	0.40603	H301	0.89311	0.88846	0.43821
H148	1.16325	0.98807	0.35216	H302	1.01364	0.85958	0.55983
H149	1.43582	0.63323	0.65782	H303	1.05168	0.8361	0.62926
H150	1.39932	0.85191	0.30221	H304	1.09666	0.78097	0.61369
H151	1.31836	0.97114	0.23747	H305	1.13714	0.89683	0.45194
C152	1.51819	0.5764	0.54078	H306	1.09125	0.94998	0.44376
C153	1.51973	0.68858	0.58669	H307	0.93939	0.99537	0.54525
H154	1.51999	0.7317	0.603	H308	1.08888	1.00318	0.57017

Table S3. Atomic coordinates of the AA-stacking mode of Cu-2D-COF.

Cu-2D-COF space group: P1							
a = 50.5102 Å, b = 50.1498 Å, c= 3.8352 Å, α = β= γ= 90°							
Atom	x	y	z	Atom	x	y	z
H1	0.26717	0.8239	0.29174	C186	0.07507	0.06112	-0.2757
H2	0.30313	0.80034	-0.01281	C187	0.02832	0.02563	0.09227
H3	0.32243	0.75789	-0.22478	C188	0.05151	0.98731	0.33194
H4	0.2234	0.82245	0.57683	C189	0.02716	0.94834	0.55914
H5	0.27902	0.68502	-0.05382	C190	0.05065	0.93047	0.59682
H6	0.18621	0.74618	0.75184	C191	0.04887	0.90416	0.47042
C7	0.43182	0.56456	-0.00983	C192	0.06859	0.88559	0.54234
C8	0.47796	0.52877	0.29557	C193	0.09089	0.89302	0.73783
C9	0.45537	0.48614	0.37892	C194	0.09366	0.9195	0.84476
C10	0.47915	0.44346	0.28482	N195	0.11143	0.8744	0.8206
C11	0.45557	0.42593	0.32551	C196	0.89035	0.14109	0.44546
C12	0.458	0.40213	0.51771	C197	0.37253	0.66956	-0.20696
C13	0.43637	0.38538	0.56628	C198	0.37488	0.69623	-0.10629
C14	0.41153	0.39251	0.43112	C199	0.35287	0.71288	-0.11029
C15	0.40922	0.41559	0.22896	C200	0.32813	0.70336	-0.2246
N16	0.38813	0.37714	0.49122	C201	0.32651	0.67721	-0.35729
C17	0.61576	0.64497	0.83539	C202	0.34847	0.66029	-0.34344
C18	0.12691	0.17015	-0.43269	C203	0.30389	0.71924	-0.15396
C19	0.12159	0.19581	-0.31007	C204	0.28038	0.70639	-0.04918
C20	0.14204	0.21403	-0.26793	N205	0.26014	0.72083	0.08737
C21	0.16818	0.20726	-0.35961	C206	0.26244	0.74742	0.14658
C22	0.17292	0.18218	-0.51303	C207	0.28463	0.76151	0.02599
C23	0.15249	0.16359	-0.54415	C208	0.30509	0.74728	-0.13357
C24	0.19042	0.22493	-0.25472	H209	0.03193	0.89785	0.31807
C25	0.21514	0.21402	-0.15924	H210	0.06647	0.86565	0.43885
N26	0.23392	0.22993	-0.01637	H211	0.11099	0.92559	0.99049
C27	0.2288	0.25561	0.07226	H212	0.90785	0.14886	0.57107
C28	0.20503	0.2678	-0.02677	H213	0.39333	0.70371	-0.00025
C29	0.18622	0.25248	-0.20295	H214	0.35503	0.73258	0.00091
H30	0.47661	0.3967	0.63821	H215	0.30806	0.66944	-0.45844
H31	0.43924	0.36747	0.71765	H216	0.34647	0.63985	-0.43058
H32	0.39034	0.42083	0.11403	H217	0.0759	0.95777	0.87981
H33	0.59833	0.65177	0.97182	H218	0.07049	0.97859	0.38589
H34	0.10194	0.2011	-0.22073	H219	0.30948	0.26066	0.53611
H35	0.13737	0.23264	-0.1402	H220	0.21884	0.19286	-0.17512

H36	0.19253	0.17647	-0.59764	H221	0.26044	0.33125	0.58565
H37	0.1568	0.14393	-0.64602	H222	0.16801	0.26187	-0.28651
H38	0.42908	0.44864	-0.00588	C223	0.43119	0.43174	0.16721
H39	0.43692	0.47655	0.44286	C224	0.47874	0.47156	0.32093
C40	0.93237	0.06638	-0.14453	C225	0.45482	0.51383	0.35216
C41	0.97962	0.02668	0.10705	C226	0.47778	0.55678	0.24372
C42	0.9555	0.98782	0.30867	C227	0.45342	0.57337	0.19131
C43	0.97881	0.94825	0.53186	C228	0.45287	0.59968	0.31972
C44	0.95586	0.92985	0.49756	C229	0.43255	0.61704	0.22911
C45	0.9596	0.90578	0.31698	C230	0.41156	0.60831	0.01835
C46	0.93836	0.88919	0.24195	C231	0.41075	0.58166	-0.08704
C47	0.91258	0.89668	0.33872	N232	0.39109	0.62618	-0.0936
C48	0.90901	0.91959	0.54199	C233	0.60791	0.34844	-0.13815
N49	0.88971	0.88145	0.24075	C234	0.13118	0.83118	0.87588
C50	0.10606	0.15012	-0.40376	C235	0.12926	0.80495	0.7544
C51	0.64044	0.65993	0.88176	C236	0.152	0.78953	0.71308
C52	0.63984	0.685	1.04291	C237	0.17705	0.79996	0.79926
C53	0.66245	0.70098	1.04652	C238	0.17844	0.82543	0.95252
C54	0.68596	0.69208	0.88818	C239	0.15574	0.84098	0.98806
C55	0.68687	0.66618	0.74815	C240	0.20144	0.78606	0.68195
C56	0.66435	0.65024	0.7458	C241	0.22319	0.80092	0.55787
C57	0.70809	0.71072	0.82159	C242	0.24409	0.78823	0.38759
C58	0.73432	0.7013	0.7952	C243	0.24269	0.76056	0.33776
C59	0.75379	0.71725	0.64148	N244	0.22224	0.74623	0.47236
C60	0.74684	0.74306	0.53456	C245	0.20206	0.75808	0.64797
N61	0.72244	0.75295	0.60405	H246	0.46832	0.60704	0.49012
C62	0.70269	0.73729	0.72712	H247	0.43319	0.63716	0.32972
H63	0.979	0.90004	0.22424	H248	0.3946	0.57458	-0.24817
H64	0.94247	0.87103	0.10153	H249	0.58806	0.34075	-0.10176
H65	0.8894	0.925	0.63156	H250	0.11044	0.79708	0.66602
H66	0.08574	0.157	-0.39235	H251	0.15001	0.77028	0.58854
H67	0.62163	0.69263	1.15409	H252	0.19721	0.8339	1.03097
H68	0.66126	0.72063	1.16269	H253	0.15754	0.86104	1.09008
H69	0.70439	0.65881	0.61737	H254	0.4315	0.54485	-0.11928
H70	0.66536	0.63086	0.62183	H255	0.43583	0.52324	0.38454
H71	0.92744	0.95294	0.79397	C256	0.93061	0.93567	0.63024
H72	0.93627	0.97907	0.33979	C257	0.979	0.97439	0.39291
C73	0.26584	0.80252	0.25612	C258	0.95581	0.01374	0.18728
C74	0.28622	0.78914	0.0804	C259	0.98007	0.05285	-0.03138
H75	0.71972	0.16813	0.29442	C260	0.95743	0.07175	-0.00307
H76	0.6856	0.19407	-0.01372	C261	0.96125	0.09585	0.17777
H77	0.67074	0.23694	-0.26241	C262	0.94012	0.11289	0.24483

H78	0.7646	0.16576	0.54943	C263	0.91448	0.10591	0.13637
H79	0.72258	0.30506	-0.19056	C264	0.91095	0.08297	-0.06652
H80	0.8135	0.23621	0.53571	N265	0.89146	0.1209	0.2335
C81	0.57413	0.43623	0.0101	C266	0.10815	0.84879	0.84481
C82	0.52736	0.47218	0.30204	C267	0.62883	0.3292	-0.21875
C83	0.54969	0.51468	0.40786	C268	0.62481	0.30266	-0.12293
C84	0.52615	0.55753	0.31458	C269	0.64533	0.28434	-0.14681
C85	0.54938	0.57514	0.38113	C270	0.67027	0.2919	-0.27885
C86	0.54565	0.59858	0.57661	C271	0.67342	0.31814	-0.40459
C87	0.56668	0.61552	0.64957	C272	0.65301	0.3368	-0.36833
C88	0.59232	0.60899	0.53699	C273	0.69335	0.27372	-0.23189
C89	0.59602	0.58635	0.32937	C274	0.71875	0.28416	-0.16135
N90	0.61502	0.62479	0.62341	N275	0.73779	0.26821	-0.023
C91	0.38557	0.35621	0.68733	C276	0.73271	0.24247	0.06511
C92	0.86498	0.84671	-0.04284	C277	0.70868	0.23045	-0.02863
C93	0.86661	0.82212	-0.21432	C278	0.68938	0.24606	-0.19024
C94	0.84478	0.80516	-0.22341	H279	0.98055	0.10115	0.27854
C95	0.82099	0.81255	-0.05961	H280	0.94405	0.13081	0.39161
C96	0.81878	0.83828	0.08396	H281	0.89143	0.07782	-0.16193
C97	0.84061	0.85522	0.09235	H282	0.08878	0.8399	0.81184
C98	0.80059	0.7923	0.0204	H283	0.60627	0.29647	-0.00626
C99	0.77382	0.79961	0.05758	H284	0.64188	0.26481	-0.03717
N100	0.75676	0.78279	0.21987	H285	0.69188	0.3246	-0.52001
C101	0.7647	0.75865	0.34377	H286	0.65623	0.35717	-0.45174
C102	0.79051	0.74944	0.28552	H287	0.92918	0.04907	-0.30719
C103	0.80849	0.76652	0.12354	H288	0.93679	0.02289	0.15206
H104	0.52637	0.60378	0.67829	C289	0.50246	0.51527	0.31083
H105	0.56255	0.63321	0.80012	C290	0.50335	0.4309	0.21864
H106	0.61551	0.58168	0.22902	H291	0.50313	0.40987	0.15462
H107	0.40205	0.34799	0.83093	C292	0.50285	0.48568	0.30727
H108	0.88515	0.81545	-0.32624	C293	0.50216	0.57014	0.23607
H109	0.84695	0.78575	-0.34342	H294	0.50253	0.59125	0.17849
H110	0.80095	0.84455	0.21732	C295	0.00383	0.01312	0.17198
H111	0.83881	0.87426	0.22324	C296	0.00268	0.93677	0.6355
H112	0.57784	0.55354	0.06507	H297	0.00225	0.91696	0.74788
H113	0.56792	0.52414	0.48433	C298	0.00352	0.98738	0.33001
C114	0.07355	0.93794	0.78011	C299	0.00419	0.0637	-0.13441
C115	0.02771	0.97428	0.41085	H300	0.00423	0.08349	-0.2479
C116	0.0518	0.01212	0.1732	C301	0.77951	0.70781	0.57769
C117	0.02839	0.0515	-0.05715	C302	0.79794	0.72403	0.40505
C118	0.05223	0.06883	-0.09213	C303	0.21959	0.30899	0.24823
C119	0.05067	0.09509	0.03429	C304	0.20043	0.29459	0.06204

C120	0.07017	0.11359	-0.04458	H305	0.78532	0.68801	0.66069
C121	0.09224	0.10613	-0.2443	H306	0.81783	0.71667	0.36248
C122	0.09517	0.07953	-0.34531	H307	0.21597	0.32975	0.31014
N123	0.11182	0.12532	-0.34336	H308	0.18216	0.30432	-0.01259
C124	0.88928	0.86202	0.01686	Cu309	0.72121	0.78804	0.39969
C125	0.35984	0.34269	0.70441	O310	0.71993	0.81723	0.71726
C126	0.35778	0.31729	0.85381	C311	0.72232	0.84239	0.56704
C127	0.33393	0.30324	0.83513	C312	0.70609	0.86509	0.70122
C128	0.31186	0.31435	0.66393	O313	0.68986	0.7888	0.08942
C129	0.31384	0.3404	0.53073	C314	0.66594	0.78811	0.27821
C130	0.33744	0.35445	0.55325	C315	0.63954	0.7873	0.10454
C131	0.28813	0.29808	0.58222	O316	0.73817	0.84616	0.33343
C132	0.26347	0.31048	0.52027	O317	0.68556	0.78872	0.09792
C133	0.24328	0.29658	0.3507	H318	0.87548	0.23251	0.30689
C134	0.24749	0.2697	0.2687	Cu319	0.77239	0.2764	0.17149
N135	0.26996	0.25712	0.37129	O320	0.80119	0.28249	-0.14233
C136	0.29067	0.27063	0.51012	C321	0.82588	0.28673	0.0101
H137	0.03384	0.10155	0.1877	C322	0.84406	0.30761	-0.13143
H138	0.06796	0.13369	0.05273	O323	0.76946	0.30541	0.49226
H139	0.11235	0.0734	-0.49397	C324	0.76689	0.33067	0.34467
H140	0.90692	0.85583	-0.11995	C325	0.78223	0.35368	0.48812
H141	0.37479	0.30802	0.97579	O326	0.83318	0.27286	0.25132
H142	0.33302	0.28347	0.94756	O327	0.75156	0.33432	0.10518
H143	0.2978	0.34951	0.38722	Cu328	0.26785	0.22137	0.1871
H144	0.3384	0.37409	0.43672	O329	0.26282	0.19279	0.50418
H145	0.07728	0.04122	-0.37409	C330	0.26417	0.16816	0.33739
H146	0.07098	0.02044	0.11998	C331	0.29043	0.15617	0.24995
C147	0.72338	0.18893	0.23538	O332	0.29875	0.21525	-0.12162
C148	0.70408	0.20362	0.05673	C333	0.32304	0.22001	0.04723
H149	0.68264	0.74478	0.72443	C334	0.34911	0.21312	-0.11284
H150	0.76724	0.81904	-0.02456	O335	0.24385	0.156	0.27217
H151	0.7392	0.68116	0.87478	O336	0.30301	0.2154	-0.11516
H152	0.82896	0.76035	0.10144	Cu337	0.22712	0.70999	0.30518
C153	0.57463	0.56998	0.24284	O338	0.19846	0.69987	0.00653
C154	0.5265	0.52937	0.34204	C339	0.17368	0.69702	0.1617
C155	0.55033	0.48702	0.37277	C340	0.15458	0.67676	0.02617
C156	0.52767	0.44425	0.24514	O341	0.2334	0.68241	0.63527
C157	0.55212	0.42767	0.20547	C342	0.23654	0.65709	0.49152
C158	0.55215	0.40142	0.33474	C343	0.22095	0.63412	0.63067
C159	0.57216	0.38371	0.24544	O344	0.1671	0.71165	0.399
C160	0.5934	0.39201	0.03572	O345	0.25246	0.65345	0.25834
C161	0.5949	0.41872	-0.06641	H346	0.69194	0.85779	0.9008

N162	0.61306	0.37348	-0.08397	H347	0.69461	0.87418	0.48529
C163	0.39479	0.65158	-0.13835	H348	0.7192	0.88047	0.81773
C164	0.86479	0.154	0.51743	H349	0.63789	0.76909	-0.05888
C165	0.86393	0.17826	0.69848	H350	0.63686	0.80531	-0.06014
C166	0.84	0.192	0.73533	H351	0.62381	0.78688	0.30623
C167	0.81659	0.18183	0.58662	H352	0.83415	0.31875	-0.3437
C168	0.81747	0.15662	0.42481	H353	0.86234	0.29812	-0.23359
C169	0.8412	0.14283	0.39296	H354	0.84948	0.32196	0.07652
C170	0.7925	0.19867	0.548	H355	0.76838	0.36905	0.58857
C171	0.76762	0.18669	0.49245	H356	0.79481	0.36262	0.28194
C172	0.74727	0.2011	0.33422	H357	0.79526	0.34683	0.70226
C173	0.75163	0.22806	0.25538	H358	0.29224	0.15363	-0.03493
N174	0.77438	0.24034	0.35579	H359	0.29232	0.13635	0.37642
C175	0.79488	0.22641	0.49502	H360	0.3067	0.16921	0.34316
H176	0.5363	0.39429	0.50044	H361	0.34647	0.20027	-0.34531
H177	0.57092	0.36354	0.34277	H362	0.36131	0.20198	0.07794
H178	0.61124	0.4255	-0.22597	H363	0.35962	0.2317	-0.18705
H179	0.41417	0.66035	-0.10028	H364	0.13692	0.6869	-0.08395
H180	0.88188	0.18696	0.80294	H365	0.14824	0.66327	0.23942
H181	0.84005	0.21089	0.87143	H366	0.16414	0.66457	-0.1793
H182	0.80029	0.14817	0.29808	H367	0.20928	0.62476	0.41846
H183	0.8412	0.12401	0.25454	H368	0.20706	0.64114	0.83454
H184	0.57491	0.45601	-0.09689	H369	0.23456	0.61905	0.74382
H185	0.56923	0.47753	0.40942				

Table S4. Atomic coordinates of the AA-stacking mode of Cu-1D-COF.

Cu-1D-COF space group: P1							
a = 41.4305 Å, b = 24.5263 Å, c= 4.0228 Å, α =88.6838^{o} β=89.8567^{o} γ= 91.5895^{o}							
Atom	x	y	z	Atom	x	y	z
C1	1.54351	0.43368	0.51024	C185	1.3247	0.44211	0.33612
C2	1.54308	0.48904	0.55532	C186	1.32244	0.49769	0.36043
C3	1.46692	0.52514	0.3977	C187	1.29389	0.52087	0.3638
C4	1.46389	0.40314	0.38355	N188	1.26923	0.48885	0.33345
C5	1.44447	0.41931	0.30359	C189	1.34818	0.5302	0.37963
C6	1.41718	0.39165	0.28124	C190	1.34592	0.58607	0.39547
C7	1.4097	0.34609	0.32507	C191	1.31789	0.60967	0.399
C8	1.42938	0.32955	0.40017	C192	1.29159	0.57696	0.39108
C9	1.45616	0.35796	0.43075	C193	1.31556	0.66585	0.40334
N10	1.38426	0.31209	0.28261	C194	1.28761	0.68858	0.41184
C11	1.38129	0.26062	0.33083	C195	1.26184	0.65499	0.42753
C12	1.35332	0.2297	0.29623	N196	1.26451	0.60044	0.40885
C13	1.32837	0.25525	0.21946	C197	1.23213	0.67818	0.46792
C14	1.30101	0.22735	0.20914	C198	1.2056	0.648	0.43886
C15	1.29761	0.17403	0.28625	C199	1.17781	0.67054	0.47414
C16	1.32322	0.14694	0.34862	C200	1.17613	0.72387	0.53576
C17	1.35087	0.17448	0.35188	C201	1.20243	0.75313	0.57071
C18	1.26768	0.14762	0.31592	C202	1.22997	0.73017	0.54257
C19	1.24161	0.17783	0.32193	N203	0.87748	0.26658	0.33251
C20	1.21401	0.15155	0.35237	C204	1.14727	0.75005	0.55759
C21	1.21252	0.09524	0.38527	C205	0.87408	0.31865	0.31206
C22	1.23923	0.06639	0.38872	C206	0.84453	0.34201	0.33475
N23	1.26567	0.09317	0.35308	C207	0.84235	0.39803	0.35406
C24	1.18523	0.0677	0.42147	C208	0.81476	0.42075	0.38759
H25	1.44664	0.50753	0.34307	C209	0.78862	0.38795	0.40105
H26	1.4022	0.40432	0.22076	C210	0.79111	0.3314	0.38397
H27	1.42385	0.29504	0.43831	C211	0.8188	0.30877	0.35136
H28	1.47087	0.34483	0.49204	C212	0.75961	0.41387	0.43851
H29	1.33	0.29743	0.17409	N213	0.7597	0.46733	0.48106
H30	1.28243	0.24822	0.14677	C214	0.73449	0.49387	0.52337
H31	1.37008	0.15342	0.40608	C215	0.70678	0.46531	0.52599
H32	1.24254	0.22141	0.31122	C216	0.70588	0.41069	0.4793
H33	1.19408	0.17517	0.3562	C217	0.73213	0.38505	0.43388
H34	1.16472	0.08984	0.42092	C218	0.73568	0.54943	0.56283
H35	1.45039	0.45349	0.2605	C219	0.70908	0.57583	0.60694
H36	1.39944	0.2395	0.39939	C220	0.68187	0.54664	0.61517

H37	1.32158	0.10519	0.40321	C221	0.6807	0.49167	0.57375
C38	1.49088	0.43647	0.42568	N222	0.76202	0.57841	0.55456
C39	1.49149	0.49343	0.44545	C223	0.7636	0.63265	0.58538
C40	1.56741	0.51443	0.64376	C224	0.73762	0.65952	0.63234
C41	1.57184	0.40418	0.49876	C225	0.71035	0.63123	0.6423
C42	1.59606	0.42829	0.39766	C226	0.79262	0.66264	0.57105
C43	1.62259	0.40076	0.38091	C227	0.81871	0.63786	0.63423
C44	1.62496	0.34788	0.45551	C228	0.84571	0.66745	0.63603
C45	1.6008	0.32346	0.55746	C229	0.8474	0.72144	0.56469
C46	1.57439	0.35152	0.57874	C230	0.82163	0.74542	0.49231
N47	1.65206	0.32045	0.42204	C231	0.79448	0.71636	0.49558
C48	1.65527	0.26818	0.43232	C232	0.87615	0.75183	0.56819
C49	1.68426	0.24465	0.39294	N233	0.87844	0.80287	0.52844
C50	1.70878	0.27718	0.33086	C234	0.90569	0.83436	0.51814
C51	1.7364	0.25415	0.29978	C235	0.92811	0.82195	0.56548
C52	1.7401	0.19782	0.33271	C236	0.95488	0.85212	0.55977
C53	1.71512	0.16553	0.3906	C237	0.95952	0.89575	0.51038
C54	1.68762	0.18861	0.42054	C238	0.93705	0.90848	0.46498
C55	1.7695	0.17159	0.31898	C239	0.91023	0.87826	0.4725
C56	1.79604	0.19933	0.26455	C240	0.98699	0.93011	0.53264
C57	1.8232	0.17291	0.27887	C241	1.01402	0.90355	0.54892
C58	1.82434	0.11874	0.34732	C242	1.04146	0.93227	0.54181
C59	1.79756	0.09005	0.39498	C243	1.04173	0.98987	0.51568
N60	1.77128	0.11558	0.37977	C244	1.01442	1.01676	0.48754
C61	1.85167	0.09309	0.37221	C245	0.98704	0.9872	0.50801
H62	1.58618	0.49113	0.69349	C246	1.06817	0.89794	0.54141
H63	1.64126	0.42008	0.30559	C247	1.07007	0.8495	0.58726
H64	1.60231	0.28319	0.62199	C248	1.09566	0.818	0.57834
H65	1.55602	0.33241	0.65831	C249	1.11994	0.83501	0.52869
H66	1.70641	0.32053	0.30655	C250	1.11833	0.88353	0.48805
H67	1.75454	0.28086	0.25242	C251	1.09245	0.91402	0.48754
H68	1.66898	0.16278	0.46783	N252	1.14539	0.80186	0.51756
H69	1.79628	0.24104	0.21297	C253	0.96042	1.01574	0.50929
H70	1.8435	0.19531	0.23929	C254	1.0683	1.0213	0.52452
H71	1.8723	0.11484	0.33574	H255	1.01333	0.22137	0.34684
H72	1.59435	0.46844	0.331	H256	1.08899	0.10309	0.64179
H73	1.63661	0.24081	0.4651	H257	0.93936	0.09404	0.61163
H74	1.71703	0.1221	0.41628	H258	1.09658	0.13369	0.33172
C75	1.51755	0.51959	0.51401	H259	1.14025	0.1934	0.28964
C76	1.51707	0.40763	0.45399	H260	1.08889	0.32985	0.44408
H77	1.51703	0.36472	0.42574	H261	1.04683	0.26864	0.52741
C78	1.49271	0.6631	0.53793	H262	0.97527	0.26092	0.52066

C79	1.49272	0.60718	0.49794	H263	0.93046	0.31003	0.451
C80	1.56785	0.5699	0.67259	H264	0.88793	0.16505	0.25183
C81	1.57534	0.69195	0.6177	H265	0.93539	0.1174	0.28577
C82	1.60165	0.67302	0.53527	H266	1.1687	0.25894	0.16325
C83	1.62794	0.70488	0.53025	H267	1.22092	0.27926	0.1037
C84	1.62771	0.75869	0.58332	H268	1.26534	0.33608	0.13246
C85	1.60134	0.77888	0.65558	H269	1.21343	0.46041	0.37756
C86	1.57622	0.74512	0.68437	H270	1.16849	0.40136	0.35378
N87	1.65461	0.79115	0.56326	H271	1.30148	0.36752	0.29833
C88	1.65647	0.84394	0.5466	H272	1.34615	0.42289	0.33941
C89	1.68589	0.87219	0.53054	H273	1.37002	0.51249	0.37676
C90	1.71212	0.84324	0.55365	H274	1.36603	0.61106	0.40183
C91	1.73998	0.86933	0.53227	H275	1.33533	0.69204	0.39793
C92	1.74238	0.92541	0.48614	H276	1.28642	0.73217	0.40767
C93	1.71586	0.95457	0.46705	H277	1.20646	0.60737	0.38648
C94	1.68794	0.92827	0.48962	H278	1.1576	0.64742	0.44657
C95	1.77191	0.95377	0.44944	H279	1.20155	0.79163	0.61972
C96	1.79929	0.92916	0.49022	H280	1.24941	0.75323	0.58306
C97	1.82615	0.95658	0.44469	H281	1.12763	0.72516	0.592
C98	1.82591	1.00893	0.36074	H282	0.8931	0.34614	0.29284
C99	1.79828	1.03351	0.32854	H283	0.86204	0.42432	0.34484
N100	1.77248	1.00531	0.37207	H284	0.81377	0.46427	0.40211
C101	1.85247	1.03719	0.3108	H285	0.77309	0.30556	0.39889
H102	1.58663	0.58721	0.747	H286	0.82031	0.26519	0.34151
H103	1.64819	0.68924	0.47254	H287	0.68497	0.38782	0.47869
H104	1.60009	0.82058	0.6913	H288	0.73061	0.34315	0.39637
H105	1.55735	0.76173	0.75481	H289	0.66162	0.56655	0.6525
H106	1.71085	0.80015	0.58718	H290	0.65948	0.46979	0.57815
H107	1.75926	0.84479	0.54936	H291	0.73868	0.70177	0.66639
H108	1.66797	0.95148	0.47153	H292	0.69043	0.65228	0.68014
H109	1.80032	0.88947	0.55867	H293	0.81791	0.59585	0.68551
H110	1.847	0.93702	0.47568	H294	0.86529	0.6481	0.69324
H111	1.87376	1.01844	0.33442	H295	0.82263	0.78662	0.43304
H112	1.60198	0.63438	0.46857	H296	0.77503	0.73544	0.4356
H113	1.63662	0.86781	0.53795	H297	0.89533	0.73105	0.6237
H114	1.71673	0.99773	0.43185	H298	0.92466	0.78965	0.61258
C115	1.54627	0.66	0.61051	H299	0.97185	0.84209	0.59828
C116	1.5445	0.60243	0.60742	H300	0.94039	0.94189	0.4245
C117	1.46818	0.5811	0.41382	H301	0.89311	0.88846	0.43821
C118	1.4651	0.69602	0.52968	H302	1.01364	0.85958	0.55983
C119	1.43736	0.67338	0.58972	H303	1.05168	0.8361	0.62926
C120	1.41122	0.70127	0.55314	H304	1.09666	0.78097	0.61369

C121	1.41234	0.75459	0.47913	H305	1.13714	0.89683	0.45194
C122	1.44021	0.78032	0.44579	H306	1.09125	0.94998	0.44376
C123	1.46621	0.75078	0.4644	H307	0.93939	0.99537	0.54525
N124	1.38484	0.78203	0.4537	H308	1.08888	1.00318	0.57017
C125	1.38101	0.82915	0.36933	Cu309	1.24216	1.54172	0.42356
C126	1.35145	0.85342	0.36835	O310	1.21466	1.51786	0.72206
C127	1.32663	0.82492	0.44648	C311	1.18313	1.54104	0.68062
C128	1.29865	0.84817	0.44373	C312	1.14726	1.513	0.71194
C129	1.29487	0.90081	0.36535	O313	1.18474	1.58429	0.61607
C130	1.32022	0.92999	0.29532	H314	1.15075	1.47366	0.74702
C131	1.34808	0.90633	0.29437	H315	1.13212	1.51762	0.50536
C132	1.2652	0.9266	0.35677	H316	1.13166	1.52649	0.9041
C133	1.2381	0.89762	0.39131	O317	0.23012	1.53736	-0.0706
C134	1.21114	0.92456	0.37946	C318	0.19858	1.51146	-0.02193
C135	1.21105	0.98023	0.33229	C319	0.16271	1.53596	-0.05649
C136	1.2385	1.00781	0.29525	O320	0.20019	1.46872	0.05161
N137	1.2643	0.98043	0.30895	H321	0.1662	1.57543	-0.10015
C138	1.18445	1.00882	0.32349	H322	0.14757	1.5311	0.15191
H139	1.44969	0.60365	0.35753	H323	0.14711	1.5199	-0.24435
H140	1.38984	0.68203	0.58655	O324	-0.20431	1.52047	-0.05705
H141	1.44206	0.82262	0.40022	C325	-0.17277	1.54637	-0.01726
H142	1.48701	0.77099	0.42341	C326	-0.13691	1.52187	-0.04681
H143	1.32902	0.78483	0.51214	O327	-0.17438	1.58911	0.0444
H144	1.2804	0.82558	0.50974	H328	-0.1404	1.48239	-0.07923
H145	1.367	0.92954	0.23638	H329	-0.12176	1.52673	0.15946
H146	1.23731	0.85451	0.4231	H330	-0.1213	1.53792	-0.23996
H147	1.19043	0.90191	0.40603	Cu331	-0.21635	1.51611	0.43895
H148	1.16325	0.98807	0.35216	O332	-0.18886	1.53996	0.72936
H149	1.43582	0.63323	0.65782	C333	-0.15732	1.51679	0.69278
H150	1.39932	0.85191	0.30221	C334	-0.12145	1.54483	0.7144
H151	1.31836	0.97114	0.23747	O335	-0.15893	1.47354	0.64042
C152	1.51819	0.5764	0.54078	H336	-0.12494	1.58417	0.73864
C153	1.51973	0.68858	0.58669	H337	-0.10631	1.5402	0.50833
H154	1.51999	0.7317	0.603	H338	-0.10585	1.53134	0.90954
C155	0.98645	0.15509	0.46163	O339	0.73731	1.05117	-0.0156
C156	0.98667	0.10185	0.54469	C340	0.70577	1.02527	0.03306
C157	1.01418	0.076	0.57477	C341	0.66991	1.04977	-0.00149
C158	1.04126	0.10616	0.55212	O342	0.70738	0.98254	0.10661
C159	1.04107	0.16001	0.47253	H343	0.6734	1.08925	-0.04515
C160	1.01365	0.18196	0.42013	H344	0.65476	1.04491	0.20691
C161	1.06865	0.0798	0.62066	H345	0.6543	1.03372	-0.18935
C162	0.96036	0.07385	0.60284	O346	-0.69943	1.04113	0.02279

C163	1.06832	0.19555	0.44079	C347	-0.66789	1.06703	0.06258
C164	0.95861	0.18456	0.4153	C348	-0.63203	1.04253	0.03304
C165	1.09497	0.17582	0.36768	O349	-0.6695	1.10977	0.12425
C166	1.11982	0.21064	0.33711	H350	-0.63552	1.00305	0.00061
C167	1.11773	0.2668	0.36319	H351	-0.61688	1.04739	0.2393
C168	1.09087	0.28678	0.42594	H352	-0.61642	1.05859	-0.16012
C169	1.06678	0.25164	0.46989	Cu353	-0.71147	1.03677	0.51879
C170	0.95682	0.2397	0.45553	O354	-0.68398	1.06062	0.8092
C171	0.93096	0.2679	0.41673	C355	-0.65244	1.03745	0.77263
C172	0.90561	0.24076	0.34805	C356	-0.61657	1.06549	0.79424
C173	0.90728	0.18599	0.30692	O357	-0.65405	0.9942	0.72026
C174	0.93408	0.15887	0.32983	H358	-0.62006	1.10483	0.81849
N175	1.14254	0.30431	0.33645	H359	-0.60143	1.06086	0.58818
C176	1.16634	0.2959	0.23845	H360	-0.60097	1.052	0.98938
C177	1.1918	0.33472	0.23658	Cu361	0.74935	1.05553	0.47856
C178	1.21923	0.31782	0.1705	O362	0.72186	1.03168	0.77706
C179	1.24472	0.3505	0.18783	C363	0.69032	1.05485	0.73562
C180	1.24338	0.4017	0.26839	C364	0.65445	1.02682	0.76694
C181	1.21528	0.42066	0.31916	O365	0.69193	1.0981	0.67107
C182	1.18972	0.38713	0.30585	H366	0.65794	0.98747	0.80201
C183	1.27103	0.43477	0.30203	H367	0.63931	1.03144	0.56035
C184	1.2991	0.41067	0.30836	H368	0.63885	1.0403	0.9591

Table S5. N_2 and CO_2 physisorption results of various samples.

Sample	S_{BET} [m^2 g^{-1}]^a	V_{Tot} [cm^3 g^{-1}]^b	V_{micro} [cm^3 g^{-1}]^c	CO_2 [cm^3 g^{-1}]^d
2D-COF	738.9	0.95	0.25	22.7
Cu-2D-COF	483.4	0.56	0.04	51.4
1D-COF	685.2	1.15	0.19	14.9
Cu-1D-COF	440.1	1.04	0.09	17.4

$^{a)}$ Specific surface area measured by BET model; $^{b)}$ The total pore volume measured at P/P_0 = 0.99; $^{c)}$ The microporous volume analyzed by t-plot method; $^{d)}$ CO_2 physisorption data in 273K.

Table S6. EXAFS fitting parameters at the Cu K-edge for various samples.

Sample	Shell	CN^a	R(Å)^b	σ^2(Å^2)^c	ΔE_0(eV)^d	R factor
Cu foil	Cu-Cu	12*	2.54±0.01	0.0089±0.0005	4.46±0.89	0.002
CuPc	Cu-N	3.81±0.65	1.4±0.01	0.0024±0.0015	2.18±2.37	0.015
CuO	Cu-O	4.05±0.37	1.93±0.01	0.0044±0.0011	-2.10±3.87	0.006
	Cu-Cu	4.61±2.27	2.92±0.01	0.0103±0.0043	-5.67±8.76	
Cu_2O	Cu-O	1.82±0.15	1.85±0.01	0.004±0.0013	7.54±1.14	0.010
	Cu-Cu	12.81±0.92	3.01±0.01	0.024±0.0017	8.99±1.40	
Cu-1D-COF	Cu-N/O	4.10±0.25	1.94±0.01	0.004±0.001	2.45±0.88	0.003
Cu-2D-COF	Cu-N/O	4.17±0.59	1.95±0.02	0.006±0.002	-0.59±1.87	0.002

^{a}*CN*, coordination number; ^{b}*R*, distance between absorber and backscatter atoms; $^c\sigma^2$, Debye-Waller factor to account for both thermal and structural disorders; $^d\Delta E_0$, inner potential correction; *R* factor indicates the goodness of the fit. $S0^2$ was fixed to 0.937, according to the experimental EXAFS fit of Cu foil by fixing CN as the known crystallographic value.

Table S7. Carboxylative cyclization of propargylic amines with CO_2.[a]

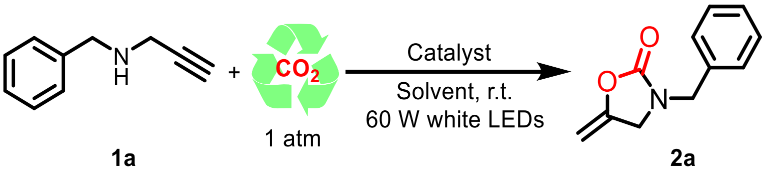

Entry	Catalyst	Solvent	Yield[b] (%)	TOF (h^{-1})
1	Cu-2D-COF	CH_3OH	99.9	78.1
2	Cu-1D-COF	CH_3OH	45.3	35.4
3	$Cu(OAc)_2$	CH_3OH	6.94	0.7
4	CuOAc	CH_3OH	6.81	0.7
5	Cu-Phen	CH_3OH	5.3	1.7
6	2D-COF	CH_3OH	n.d.	-
7	1D-COF	CH_3OH	n.d.	-
8[c]	Cu-2D-COF	CH_3OH	86.8	84.7
9[d]	Cu-2D-COF	CH_3OH	65.3	102.0
10[e]	Cu-2D-COF	CH_3OH	48.2	125.4
11[f]	Cu-2D-COF	CH_3OH	17.6	137.1
12	Cu-2D-COF	CH_3CN	95.3	74.4
13	Cu-2D-COF	DMF	88.4	69.1
14	Cu-2D-COF	THF	33.5	26.1
15	Cu-2D-COF	Toluene	25.8	20.1
16[g]	Cu-2D-COF	CH_3OH	n.d.	-
17[h]	Cu-2D-COF	CH_3OH	99.9	15.6
18[i]	Cu-2D-COF	CH_3OH	24.6	19.2

[a]Reaction conditions: 1a (73uL, 0.5 mmol), Cat. (1.32 mmol% based on Cu), CH_3OH 5mL, 1 atm CO_2, 60W White LED, room temperature for 60min. [b]Yield as determined by gas chromatography using mesitylene as the internal standard. [c]Cat. 8mg, [d]Cat. 5mg, [e]Cat. 3mg, [f]Cat. 1mg, [g]N_2 instead of CO_2, [h]10% CO_2/N_2 by volume, 6h. [i]Dark.

Table S8. 2D-COF loading with varying amounts of $Cu(OAc)_2$ for the carboxylative cyclization of propargylic amines with CO_2.

Entry	2D-COF Amount (mg)	$Cu(OAc)_2$ Amount (mg)	Yield[b] (%)
1	50	5	53.8
2	50	10	99.9
3	50	15	94.3
4	50	25	84.0
5	50	35	64.1

[a]Reaction conditions: 1a (73uL, 0.5 mmol), Cat. (10mg), CH_3OH 5mL, 1 atm CO_2, 60W White LED, room temperature for 60min. [b]Yield as determined by gas chromatography using mesitylene as the internal standard.

Section 4. NMR data and spectra of oxazolidinones

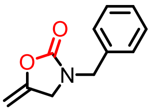

2a: ^{1}H NMR (600 MHz, $CHCl_3$) δ 7.41 -7.21 (m, 5H), 4.74 (q, *J* = 2.8 Hz, 1H), 4.47 (s, 2H), 4.24 (q, *J* = 2.5 Hz, 1H), 4.02 (t, *J* = 2.4 Hz, 2H). ^{13}C NMR (150 MHz, $CDCl_3$) δ 155.66, 148.99, 135.01, 129.00, 128.27, 128.19, 86.75, 47.90, 47.26.

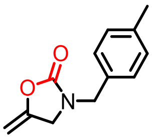

2b:^{1}H NMR (600 MHz, $CHCl_3$) δ 7.16 (s, 4H), 4.73 (q, *J* = 2.7 Hz, 1H), 4.42 (s, 2H), 4.25 - 4.17 (m, 1H), 4.00 (t, *J* = 2.4 Hz, 2H), 2.35 (s, 3H). ^{13}C NMR (150 MHz, $CDCl_3$) δ 155.60, 149.06, 138.07, 131.93, 129.62, 128.20, 86.62, 47.60, 47.16, 21.11.

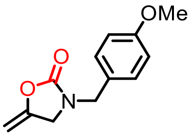

2c:^{1}H NMR (600 MHz, $CHCl_3$) δ 7.20 (d, *J* = 8.6 Hz, 2H), 6.89 (d, *J* = 8.6 Hz, 2H), 4.72 (q, *J* = 2.8 Hz, 1H), 4.40 (s, 2H), 4.31 - 4.15 (m, 1H), 3.99 (s, 2H), 3.81 (s, 3H). ^{13}C NMR (150 MHz, $CDCl_3$) δ 159.60, 155.55, 149.07, 129.59, 127.02, 114.34, 86.60, 55.31, 47.29, 47.10.

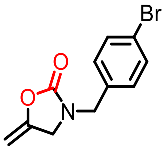

2d:^{1}H NMR (600 MHz, $CHCl_3$) δ 7.50 (d, *J* = 8.0 Hz, 2H), 7.16 (d, *J* = 8.1 Hz, 2H), 4.76 (d, *J* = 2.9 Hz, 1H), 4.42 (s, 2H), 4.26 (d, *J* = 2.7 Hz, 1H), 4.02 (s, 2H). ^{13}C NMR (150 MHz, $CDCl_3$) δ 155.61, 148.69, 134.06, 132.16, 129.83, 122.33, 87.05, 47.29, 47.21.

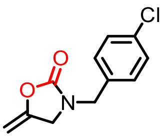

2e:^{1}H NMR (600 MHz, $CHCl_3$) δ 7.34 (d, *J* = 8.3 Hz, 2H), 7.22 (d, *J* = 8.4 Hz, 2H), 4.76 (q, *J* = 2.7 Hz, 1H), 4.44 (s, 2H), 4.26 (dt, *J* = 3.4, 2.2 Hz, 1H), 4.02 (t, *J* = 2.4 Hz, 2H). ^{13}C NMR (150 MHz, $CDCl_3$) δ 155.60, 148.71, 134.26, 133.54, 129.52, 129.19, 87.03, 47.23, 47.21.

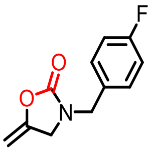

2f:^{1}H NMR (600 MHz, $CHCl_3$) δ 7.32 - 7.18 (m, 2H), 7.06 (t, *J* = 8.6 Hz, 2H), 4.75 (q, *J* = 2.7 Hz, 1H), 4.44 (s, 2H), 4.25 (dt, *J* = 3.2, 2.2 Hz, 1H), 4.02 (t, *J* = 2.4 Hz, 2H). ^{13}C NMR (150 MHz, $CDCl_3$) δ 163.46, 161.82, 155.58, 148.79, 130.86, 130.83, 129.97, 129.91, 116.00, 115.86, 86.93, 47.17.

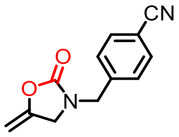

2g:^{1}H NMR (600 MHz, $CHCl_3$) δ 7.68 (d, *J* = 8.3 Hz, 2H), 7.41 (d, *J* = 8.0 Hz, 2H), 4.80 (d, *J* = 2.9 Hz, 1H), 4.54 (s, 2H), 4.31 (d, *J* = 2.8 Hz, 1H), 4.08 (t, *J* = 2.3 Hz, 2H). ^{13}C NMR (150 MHz, $CDCl_3$) δ 155.77, 148.40, 140.53, 132.88, 128.68, 118.42, 112.32, 87.58, 47.54, 47.51.

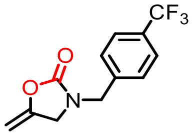

2h:^{1}H NMR (600 MHz, $CHCl_3$) δ 7.64 (d, *J* = 7.9 Hz, 2H), 7.41 (d, *J* = 7.9 Hz, 2H), 4.78 (d, *J* = 2.9 Hz, 1H), 4.54 (s, 2H), 4.29 (d, *J* = 2.7 Hz, 1H), 4.06 (t, *J* = 2.4 Hz, 2H). ^{13}C NMR (150 MHz, $CDCl_3$) δ 155.75, 148.57, 139.14, 128.43, 126.06, 126.02, 125.30, 122.59, 87.35, 47.46, 47.38.

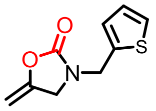

2i:^{1}H NMR (600 MHz, $CHCl_3$) δ 7.29 (dd, *J* = 5.1, 1.3 Hz, 1H), 7.02 (dd, *J* = 3.5, 1.2 Hz, 1H), 6.99 (dd, *J* = 5.1, 3.4 Hz, 1H), 4.75 (q, *J* = 2.8 Hz, 1H), 4.65 (d, *J* = 0.8 Hz, 2H), 4.26 (dt, *J* = 3.2, 2.2 Hz, 1H), 4.10 (t, *J* = 2.4 Hz, 2H). ^{13}C NMR (150 MHz, $CDCl_3$) δ 155.18, 148.83, 137.09, 127.46, 127.19, 126.27, 86.95, 47.09, 42.25.

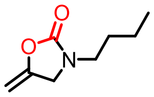

2j:^{1}H NMR (600 MHz, $CHCl_3$) δ 4.74 (q, *J* = 2.7 Hz, 1H), 4.28 (d, *J* = 2.6 Hz, 1H), 4.16 (t, *J* = 2.4 Hz, 2H), 3.30 (t, *J* = 7.3 Hz, 2H), 1.60 - 1.48 (m, 2H), 1.41 - 1.30 (m, 2H), 0.95 (t, *J* = 7.4 Hz, 3H). ^{13}C NMR (150 MHz, $CDCl_3$) δ 155.60, 149.21, 86.37, 47.81, 43.51, 29.32, 19.79, 13.63.

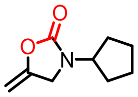

2k:^{1}H NMR (600 MHz, $CHCl_3$) δ 4.73 (q, *J* = 2.8 Hz, 1H), 4.28 (s, 1H), 4.26 (t, *J* = 6.9 Hz, 1H), 4.14 (t, *J* = 2.4 Hz, 2H), 1.96 - 1.85 (m, 2H), 1.66 - 1.50 (m, 6H). ^{13}C NMR (150 MHz, $CDCl_3$) δ 155.14, 149.39, 86.26, 54.36, 44.34, 28.89, 23.82.

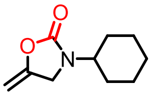

2l:^{1}H NMR (600 MHz, $CHCl_3$) δ 4.72 (q, *J* = 2.8 Hz, 1H), 4.30 – 4.23 (m, 1H), 4.13 (t, *J* = 2.4 Hz, 2H), 3.79 – 3.64 (m, 1H), 1.82 (d, *J* = 9.0 Hz, 4H), 1.71 - 1.66 (m, 1H), 1.35 (d, *J* = 8.9 Hz, 4H), 1.09 (dd, *J* = 12.8, 3.1 Hz, 1H). ^{13}C NMR (150 MHz, $CDCl_3$) δ 154.90, 149.67, 86.19, 52.38, 44.12, 30.28, 25.27, 25.25.

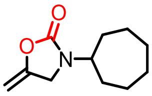

2m:^{1}H NMR (600 MHz, $CHCl_3$) δ 4.71 (d, *J* = 2.8 Hz, 1H), 4.27 (d, *J* = 2.6 Hz, 1H), 4.15 (t, *J* = 2.5 Hz, 2H), 3.91 (td, *J* = 10.3, 5.0 Hz, 1H), 1.77 – 1.51 (m, 12H). ^{13}C NMR (151 MHz, $CDCl_3$) δ 154.67, 149.59, 86.11, 54.42, 44.10, 32.47, 27.63, 24.37.

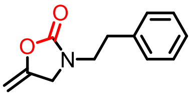

2n: ^{1}H NMR (600 MHz, $CDCl_3$) δ 7.39 - 7.14 (m, 5H), 4.70 (d, *J* = 2.9 Hz, 1H), 4.21 (d, *J* = 2.9 Hz, 1H), 3.99 (t, *J* = 2.4 Hz, 2H), 3.56 (t, *J* = 7.3 Hz, 2H), 2.89 (t, *J* = 7.3 Hz, 2H). ^{13}C NMR (150 MHz, $CDCl_3$) δ 155.47, 149.04, 137.97, 128.80, 128.60, 126.83, 86.45, 48.43, 45.19, 33.96.

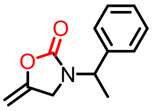

2o: ^{1}H NMR (600 MHz, $CDCl_3$) δ 7.47 - 7.28 (m, 5H), 5.26 (q, *J* = 7.1 Hz, 1H), 4.71 (q, *J* = 2.8 Hz, 1H), 4.21 (q, *J* = 2.3 Hz, 1H), 4.16 - 4.02 (m, 1H), 3.86 - 3.63 (m, 1H), 1.60 (d, *J* = 7.1 Hz, 3H). ^{13}C NMR (150 MHz, $CDCl_3$) δ 155.10, 149.27, 138.85, 128.85, 128.15, 126.98, 86.53, 51.36, 43.68, 16.41.

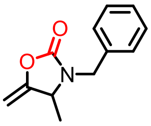

2p: ^{1}H NMR (600 MHz, $CDCl_3$) δ 7.46 - 7.15 (m, 5H), 4.83 (d, *J* = 15.3 Hz, 1H), 4.72 (dd, *J* = 3.2, 2.5 Hz, 1H), 4.21 (dd, *J* = 3.3, 2.1 Hz, 1H), 4.18 (dt, *J* = 6.4, 2.3 Hz, 1H), 4.12 (d, *J* = 15.3 Hz, 1H), 1.33 (d, *J* = 6.4 Hz, 3H). ^{13}C NMR (150 MHz, $CDCl_3$) δ 155.68, 155.13, 135.24, 128.92, 128.14, 128.11, 85.88, 53.20, 45.54, 19.24.

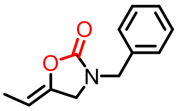

2q: ^{1}H NMR (600 MHz, $CDCl_3$) δ 7.43 - 7.19 (m, 5H), 4.60 - 4.51 (m, 1H), 4.45 (s, 2H), 3.96 (t, *J* = 2.3 Hz, 2H), 1.67 (d, *J* = 7.0 Hz, 3H). ^{13}C NMR (150 MHz, $CDCl_3$) δ 156.04, 141.70, 135.20, 128.91, 128.13, 97.54, 47.86, 47.09, 9.92.

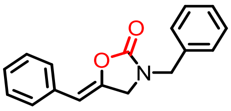

2r: ^{1}H NMR (600 MHz, $CDCl_3$) δ 7.54 (d, *J* = 7.7 Hz, 2H), 7.43 - 7.25 (m, 8H), 5.45 (s, 1H), 4.53 (s, 2H), 4.17 (s, 2H). ^{13}C NMR (150 MHz, $CDCl_3$) δ 155.60, 141.70, 134.90, 133.38, 129.03, 128.48, 128.33, 128.23, 128.20, 126.87, 103.19, 48.20, 47.94.

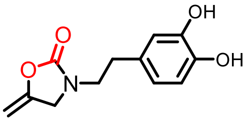

2s: ^{1}H NMR (600 MHz, DMSO-d_6) δ 8.77 (d, *J* = 26.9 Hz, 2H), 6.68 - 6.57 (m, 2H), 6.46 (dd, *J* = 8.0, 2.1 Hz, 1H), 4.61 (d, *J* = 2.7 Hz, 1H), 4.32 (d, *J* = 2.5 Hz, 1H), 4.14 (t, *J* = 2.4 Hz, 2H), 2.62 (t, *J* = 7.2 Hz, 2H). ^{13}C NMR (150 MHz, DMSO) δ 155.15, 150.33, 145.68, 144.23, 129.65, 119.69, 116.39, 116.08, 86.06, 48.04, 45.21, 32.71.

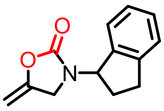

2t:^{1}H NMR (600 MHz, $CHCl_3$) δ 7.33 - 7.13 (m, 4H), 5.59 (dd, *J* = 8.2, 6.8 Hz, 1H), 4.74 (d, *J* = 2.8 Hz, 1H), 4.28 - 4.12 (m, 1H), 3.98 (d, *J* = 14.3 Hz, 1H), 3.81 (d, *J* = 14.3 Hz, 1H), 3.10 - 2.85 (m, 2H), 2.47 (dtd, *J* = 13.1, 8.4, 4.6 Hz, 1H), 2.00 (ddt, *J* = 13.7, 8.9, 7.1 Hz, 1H). ^{13}C NMR (150 MHz, $CDCl_3$) δ 155.44, 149.31, 143.47, 139.17, 128.61, 127.05, 125.15, 124.33, 86.66, 58.12, 43.71, 30.38, 29.14.

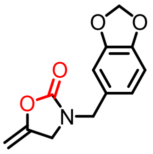

2u: ^{1}H NMR (600 MHz, $CHCl_3$) δ 6.82 - 6.75 (m, 2H), 6.73 (dd, *J* = 7.9, 1.8 Hz, 1H), 5.97 (s, 2H), 4.73 (d, *J* = 2.7 Hz, 1H), 4.36 (s, 2H), 4.24 (d, *J* = 2.7 Hz, 1H), 4.01 (d, *J* = 2.4 Hz, 2H). ^{13}C NMR (150 MHz, $CDCl_3$) δ 155.54, 148.97, 148.26, 147.65, 128.75, 121.74, 108.55, 108.44, 101.27, 86.74, 47.67, 47.10.

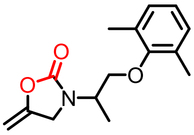

2v: ^{1}H NMR (600 MHz, $CHCl_3$) δ 7.00 (d, *J* = 7.4 Hz, 2H), 6.96 - 6.90 (m, 1H), 4.76 (d, *J* = 2.9 Hz, 1H), 4.51 - 4.44 (m, 1H), 4.37 - 4.26 (m, 3H), 3.93 - 3.86 (m, 1H), 3.82 (ddd, *J* = 9.8,

5.3, 1.0 Hz, 1H), 2.25 (s, 6H), 1.43 (dd, J = 7.0, 1.0 Hz, 3H). ^{13}C NMR (150 MHz, $CDCl_3$) δ 155.26, 154.96, 149.64, 130.67, 129.12, 124.30, 86.43, 73.28, 49.24, 46.01, 16.27, 14.60.

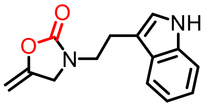

2w: ^{1}H NMR (600 MHz, $CHCl_3$) δ 8.15 (s, 1H), 7.59 (d, J = 7.9 Hz, 1H), 7.38 (d, J = 8.1 Hz, 1H), 7.24 - 7.10 (m, 2H), 7.06 (d, J = 2.3 Hz, 1H), 4.69 (d, J = 2.8 Hz, 1H), 4.18 (d, J = 2.7 Hz, 1H), 4.03 (s, 2H), 3.66 (t, J = 7.2 Hz, 2H), 3.05 (t, J = 7.1 Hz, 2H). ^{13}C NMR (150 MHz, $CDCl_3$) δ 155.71, 149.18, 136.36, 127.15, 122.31, 122.04, 119.63, 118.44, 112.06, 111.42, 86.49, 48.36, 44.00, 23.60.

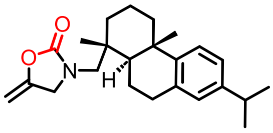

2x: ^{1}H NMR (600 MHz, $CHCl_3$) δ 7.17 (d, J = 8.2 Hz, 1H), 6.99 (dd, J = 8.2, 2.1 Hz, 1H), 6.90 (d, J = 2.1 Hz, 1H), 4.71 (d, J = 2.8 Hz, 1H), 4.24 (d, J = 2.6 Hz, 3H), 3.24 - 3.05 (m, 2H), 3.01 - 2.73 (m, 3H), 2.30 (d, J = 13.1 Hz, 1H), 1.91 (d, J = 7.2 Hz, 1H), 1.87 - 1.61 (m, 3H), 1.40 (ddd, J = 25.8, 12.2, 3.2 Hz, 3H), 1.22 (d, J = 7.0 Hz, 9H), 0.98 (s, 3H). ^{13}C NMR (150 MHz, $CDCl_3$) δ 157.24, 149.05, 147.05, 145.79, 134.55, 127.03, 124.12, 123.98, 86.35, 57.06, 52.20, 45.24, 39.08, 38.24, 37.54, 37.27, 33.50, 30.07, 25.61, 24.06, 24.02, 19.10, 18.92, 18.67.

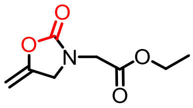

2y: ^{1}H NMR (600 MHz, $CDCl_3$) δ 4.74 (q, J = 2.8 Hz, 1H), 4.33 - 4.26 (m, 1H), 4.25 (t, J = 2.4 Hz, 2H), 4.16 (q, J = 7.2 Hz, 2H), 3.60 (t, J = 6.5 Hz, 2H), 2.63 (t, J = 6.4 Hz, 2H), 1.27 (t, J = 7.2 Hz, 3H). ^{13}C NMR (150 MHz, $CDCl_3$) δ 171.45, 155.51, 149.12, 86.63, 61.02, 48.77, 39.72, 32.82, 14.15.

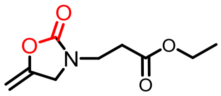

2z: ^{1}H NMR (600 MHz, $CDCl_3$) δ 4.79 (q, J = 2.8 Hz, 1H), 4.34 (s, 1H), 4.31 (t, J = 2.4 Hz, 2H), 4.23 (q, J = 7.1 Hz, 2H), 4.06 (s, 2H), 1.30 (t, J = 7.2 Hz, 3H). ^{13}C NMR (150 MHz, $CDCl_3$) δ 167.96, 155.86, 148.83, 87.06, 61.74, 48.34, 44.90, 14.14.

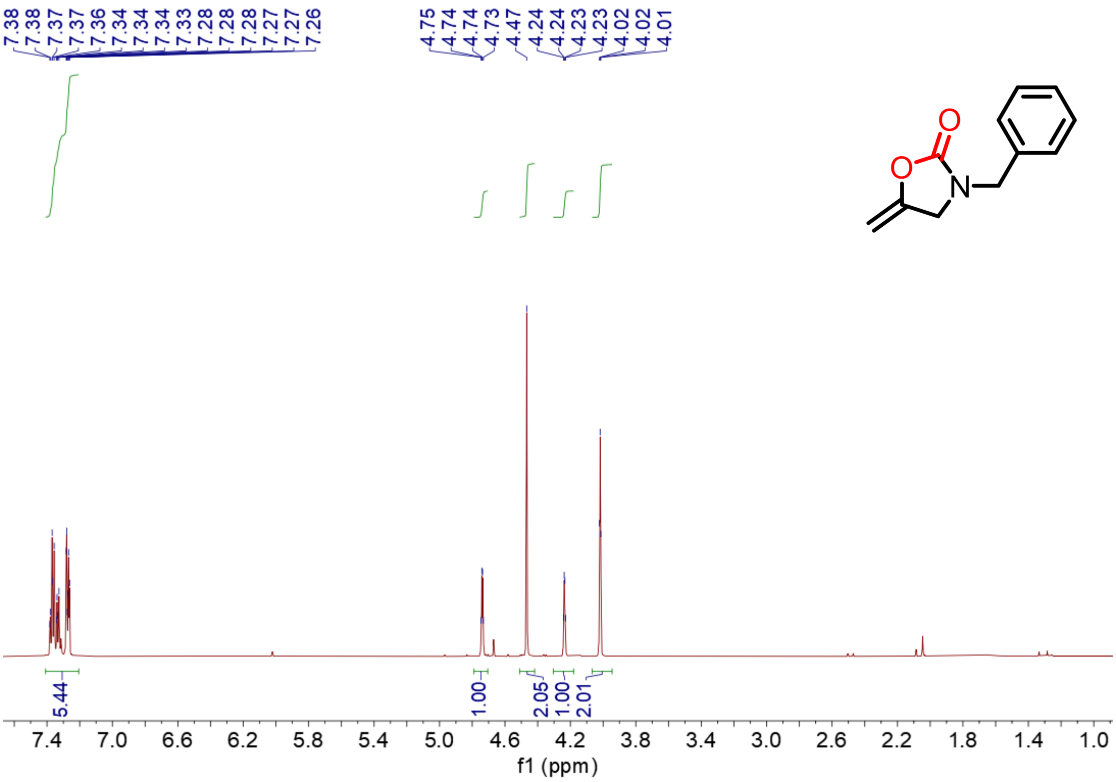
7.38
7.38
7.37
7.37
7.36
7.34
7.34
7.34
7.33
7.28
7.28
7.28
7.27
7.27
7.26
4.75
4.74
4.74
4.73
4.47
4.24
4.24
4.23
4.23
4.02
4.02
4.01
O
O
N
5.44
1.00
2.05
1.00
2.01
7.4 7.0 6.6 6.2 5.8 5.4 5.0 4.6 4.2 3.8 3.4 3.0 2.6 2.2 1.8 1.4 1.0
f1 (ppm)

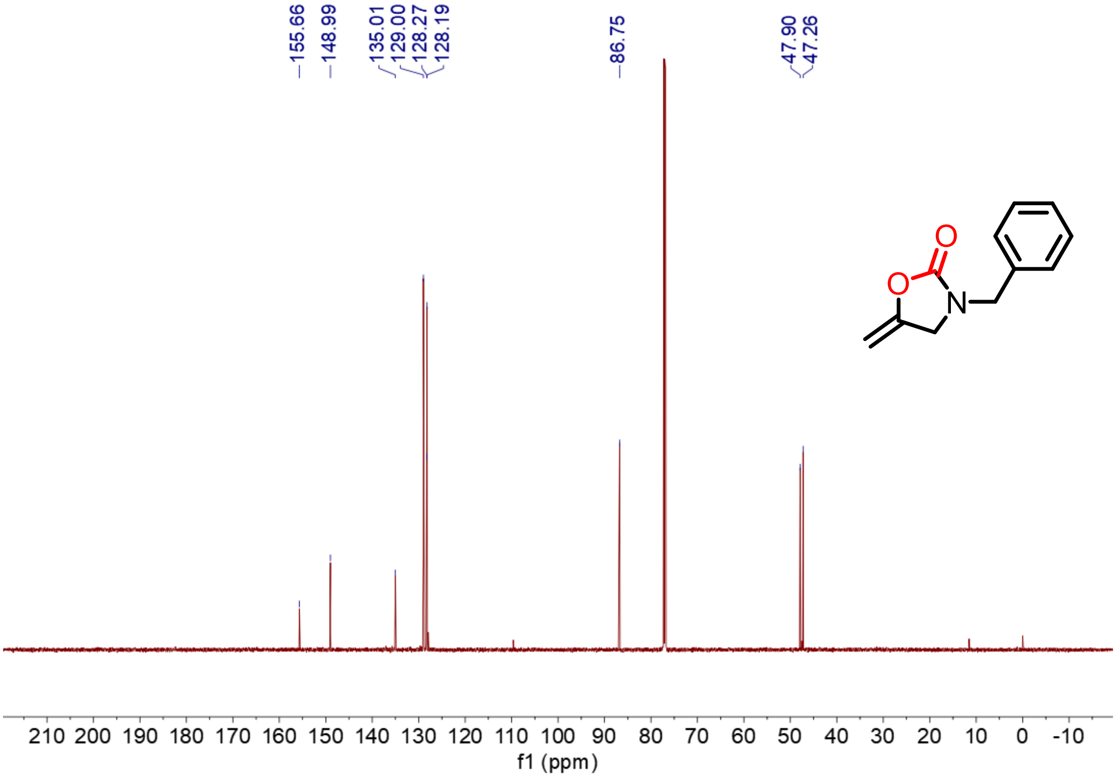
155.66
148.99
135.01
129.00
128.27
128.19
86.75
47.90
47.26
O
O
N
210 200 190 180 170 160 150 140 130 120 110 100 90 80 70 60 50 40 30 20 10 0 -10
f1 (ppm)

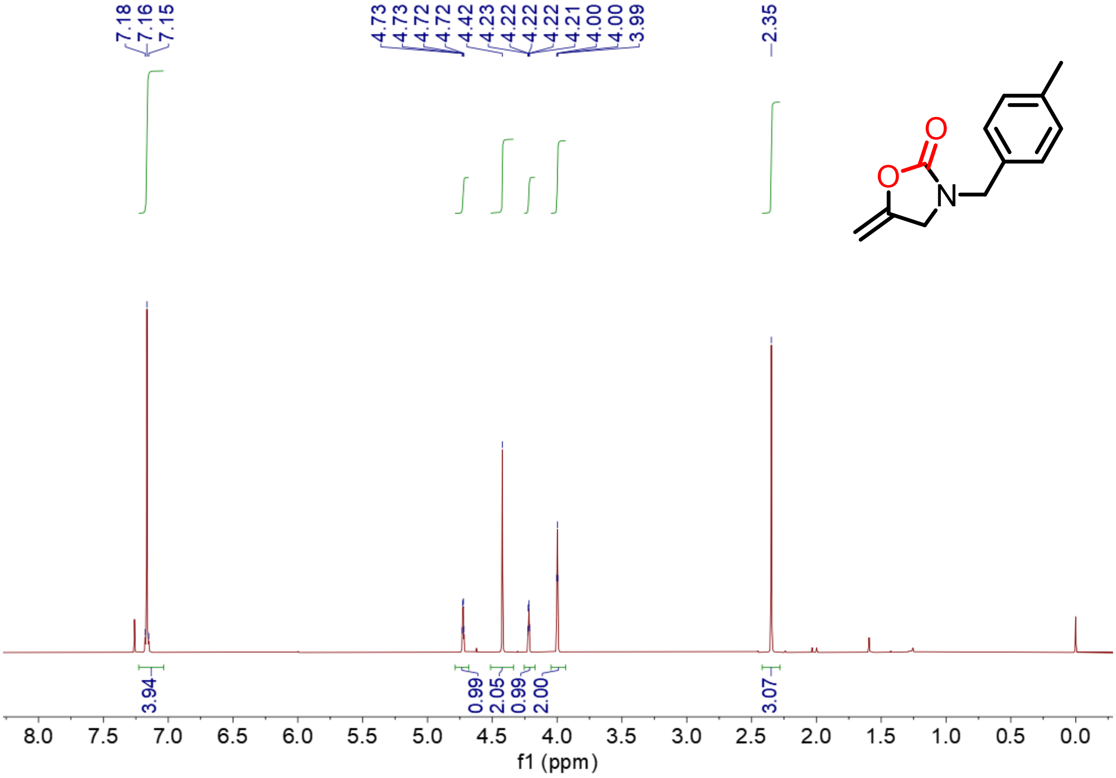
7.18
7.16
7.15
4.73
4.73
4.72
4.72
4.42
4.23
4.22
4.22
4.22
4.21
4.00
4.00
3.99
2.35
O
O
N
3.94
0.99
2.05
0.99
2.00
3.07
8.0 7.5 7.0 6.5 6.0 5.5 5.0 4.5 4.0 3.5 3.0 2.5 2.0 1.5 1.0 0.5 0.0
f1 (ppm)

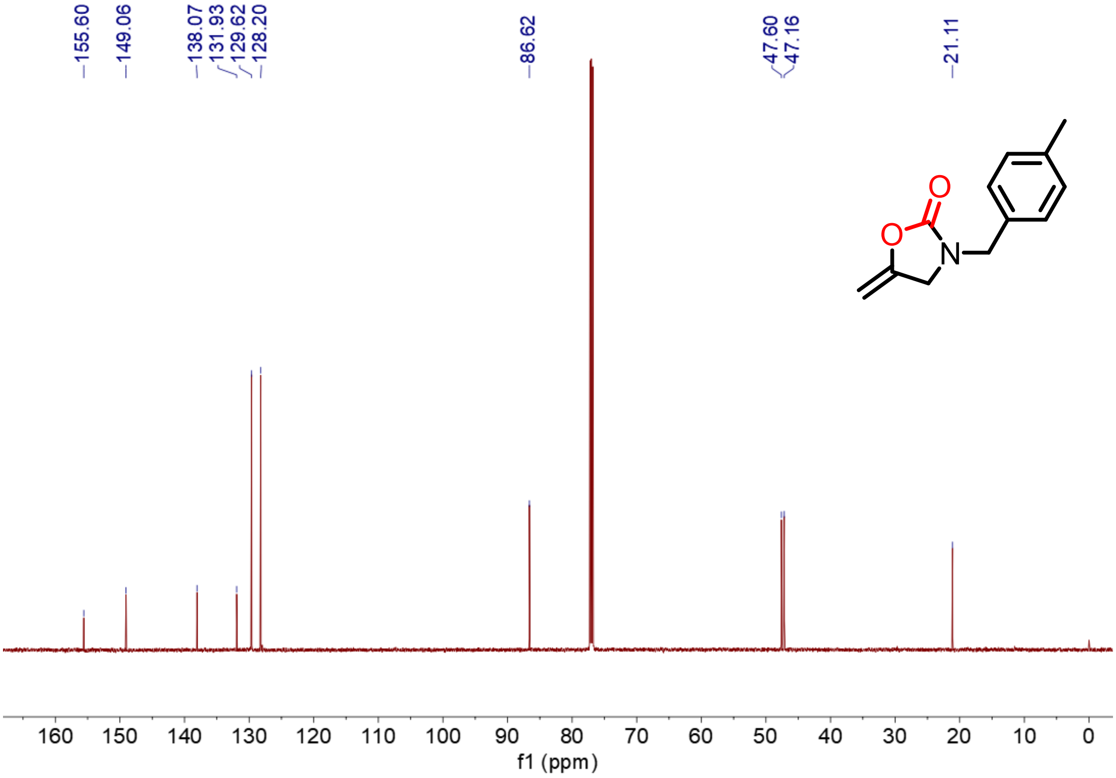
155.60
149.06
138.07
131.93
129.62
128.20
86.62
47.60
47.16
21.11
O
O
N
160 150 140 130 120 110 100 90 80 70 60 50 40 30 20 10 0
f1 (ppm)

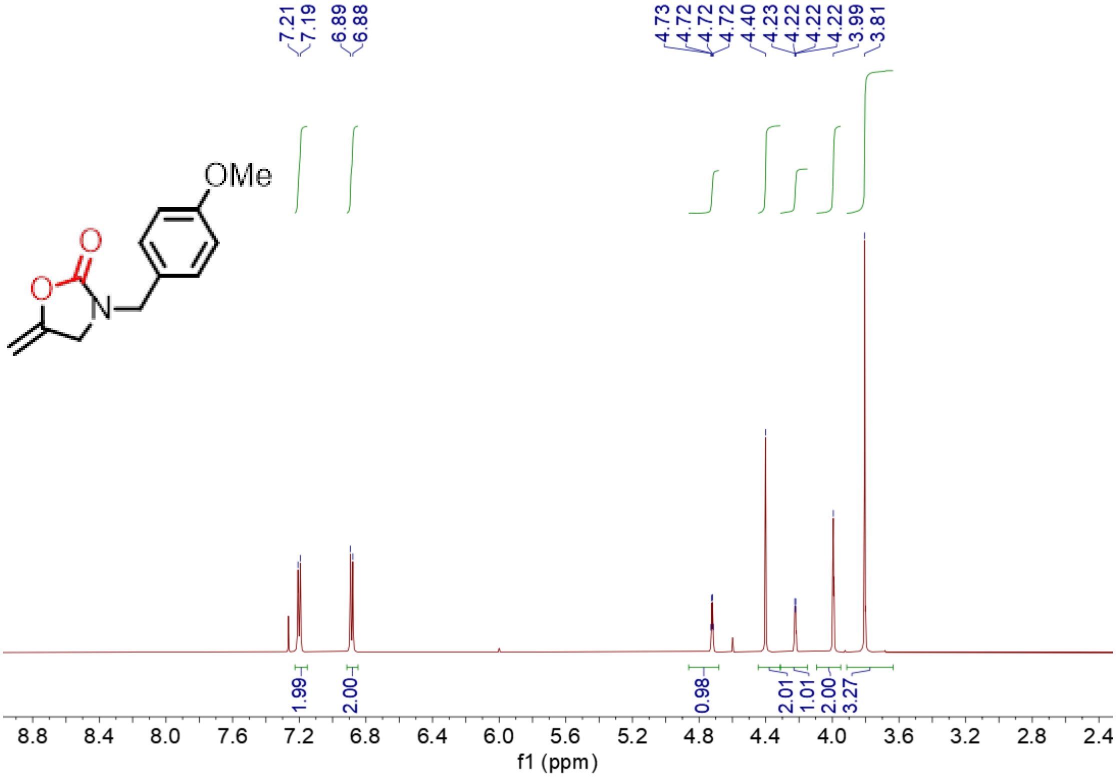
7.21
7.19
6.89
6.88
4.73
4.72
4.72
4.72
4.40
4.23
4.22
4.22
4.22
3.99
3.81
OMe
O
O
N
1.99
2.00
0.98
2.01
1.01
2.00
3.27
8.8 8.4 8.0 7.6 7.2 6.8 6.4 6.0 5.6 5.2 4.8 4.4 4.0 3.6 3.2 2.8 2.4
f1 (ppm)

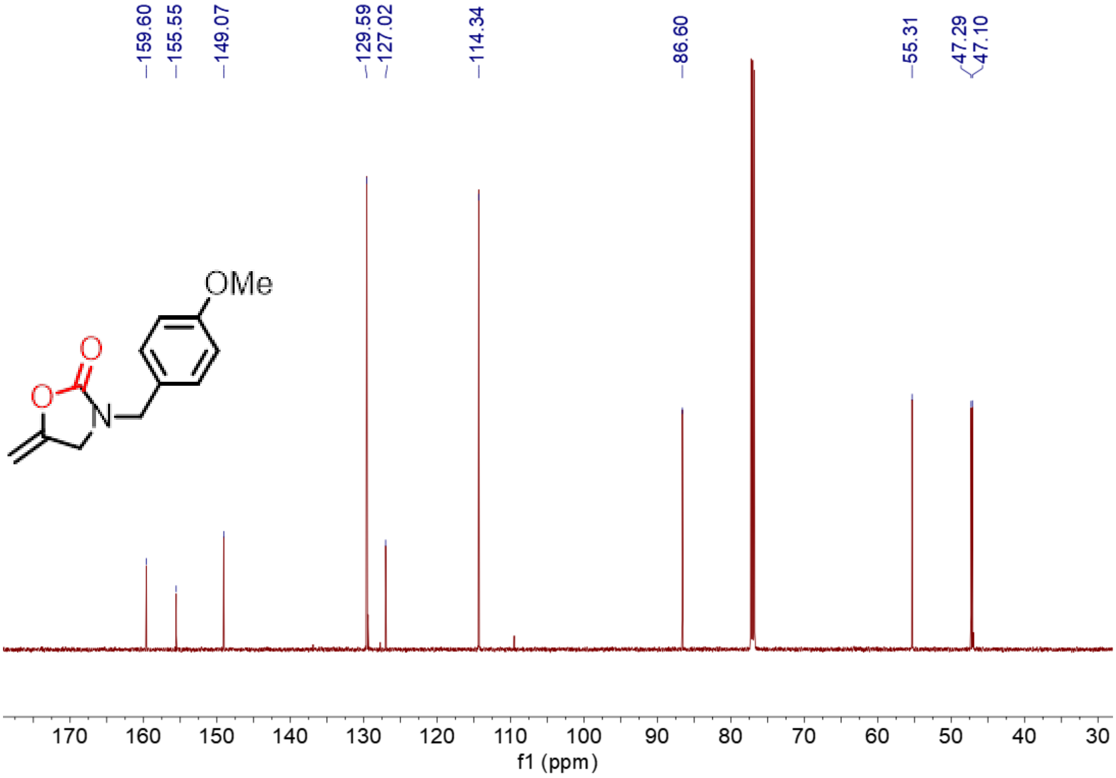
159.60
155.55
149.07
129.59
127.02
114.34
86.60
55.31
47.29
47.10
OMe
O
O
N
170 160 150 140 130 120 110 100 90 80 70 60 50 40 30
f1 (ppm)

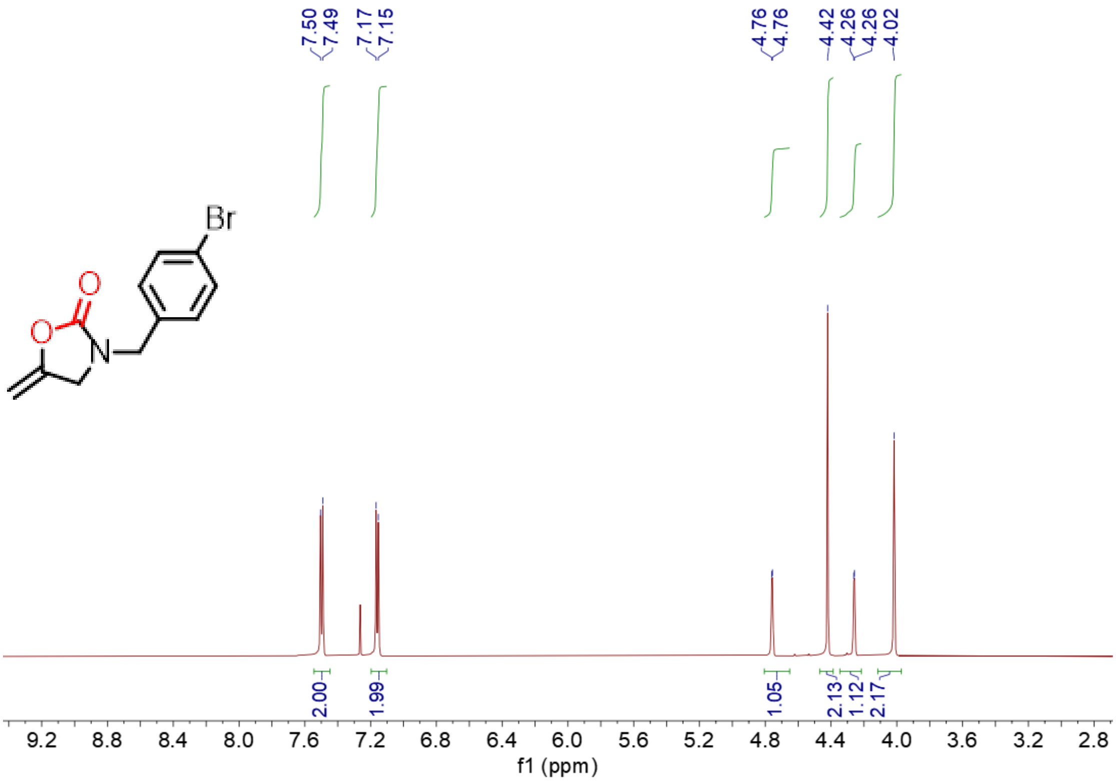
7.50
7.49
7.17
7.15
4.76
4.76
4.42
4.26
4.26
4.02
Br
O
O
N
2.00
1.99
1.05
2.13
1.12
2.17
9.2
8.8
8.4
8.0
7.6
7.2
6.8
6.4
6.0
5.6
5.2
4.8
4.4
4.0
3.6
3.2
2.8
f1 (ppm)

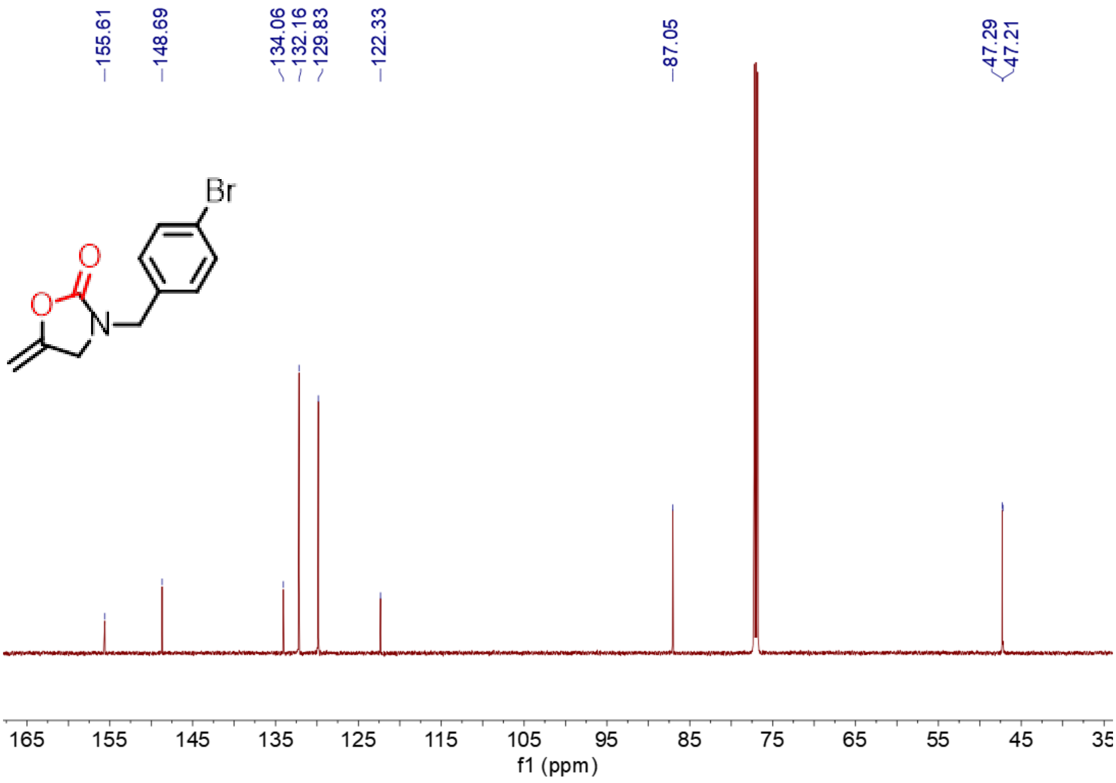
155.61
148.69
134.06
132.16
129.83
122.33
87.05
47.29
47.21
Br
O
O
N
165
155
145
135
125
115
105
95
85
75
65
55
45
35
f1 (ppm)

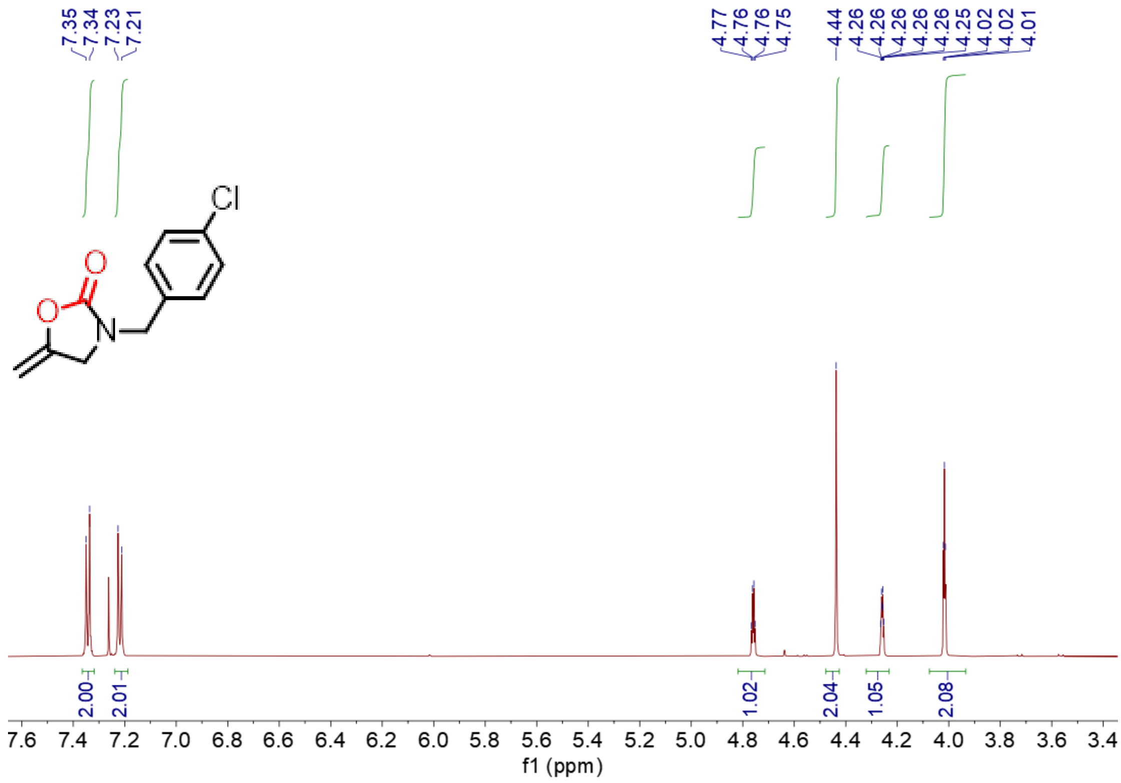
7.35
7.34
7.23
7.21
4.77
4.76
4.76
4.75
4.44
4.26
4.26
4.26
4.26
4.26
4.25
4.02
4.02
4.01
Cl
O
O
N
2.00
2.01
1.02
2.04
1.05
2.08
7.6 7.4 7.2 7.0 6.8 6.6 6.4 6.2 6.0 5.8 5.6 5.4 5.2 5.0 4.8 4.6 4.4 4.2 4.0 3.8 3.6 3.4
f1 (ppm)

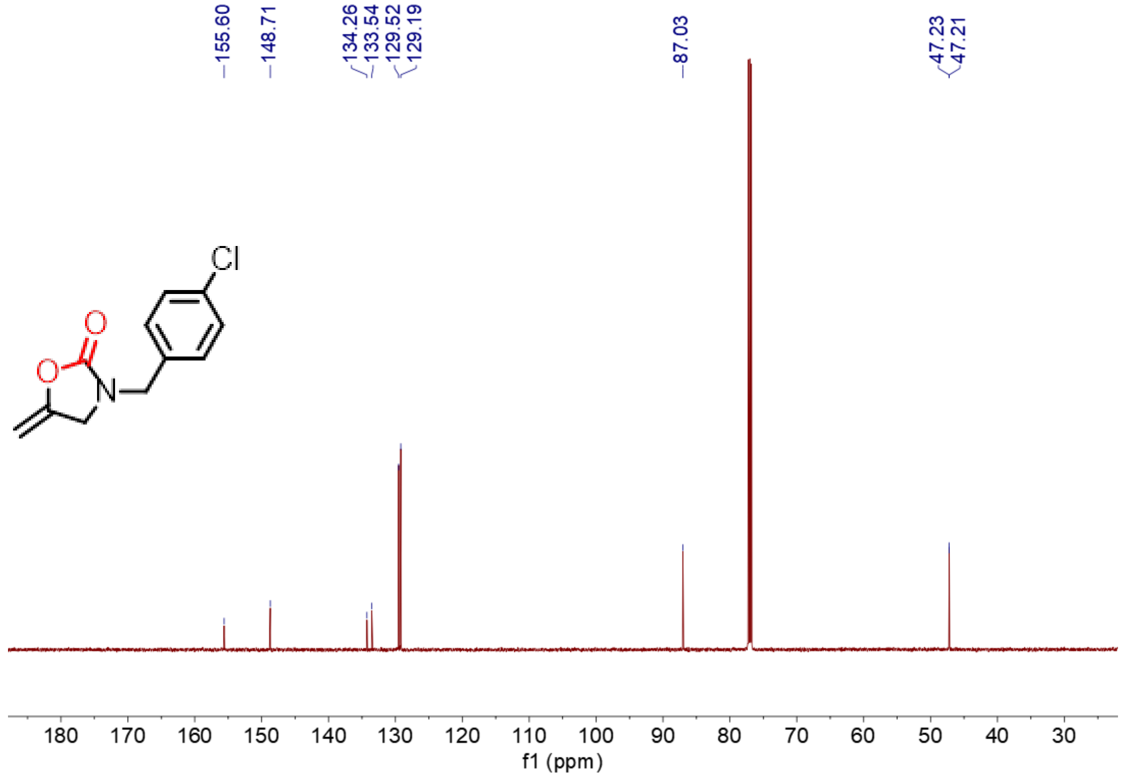
155.60
148.71
134.26
133.54
129.52
129.19
87.03
47.23
47.21
Cl
O
O
N
180 170 160 150 140 130 120 110 100 90 80 70 60 50 40 30
f1 (ppm)

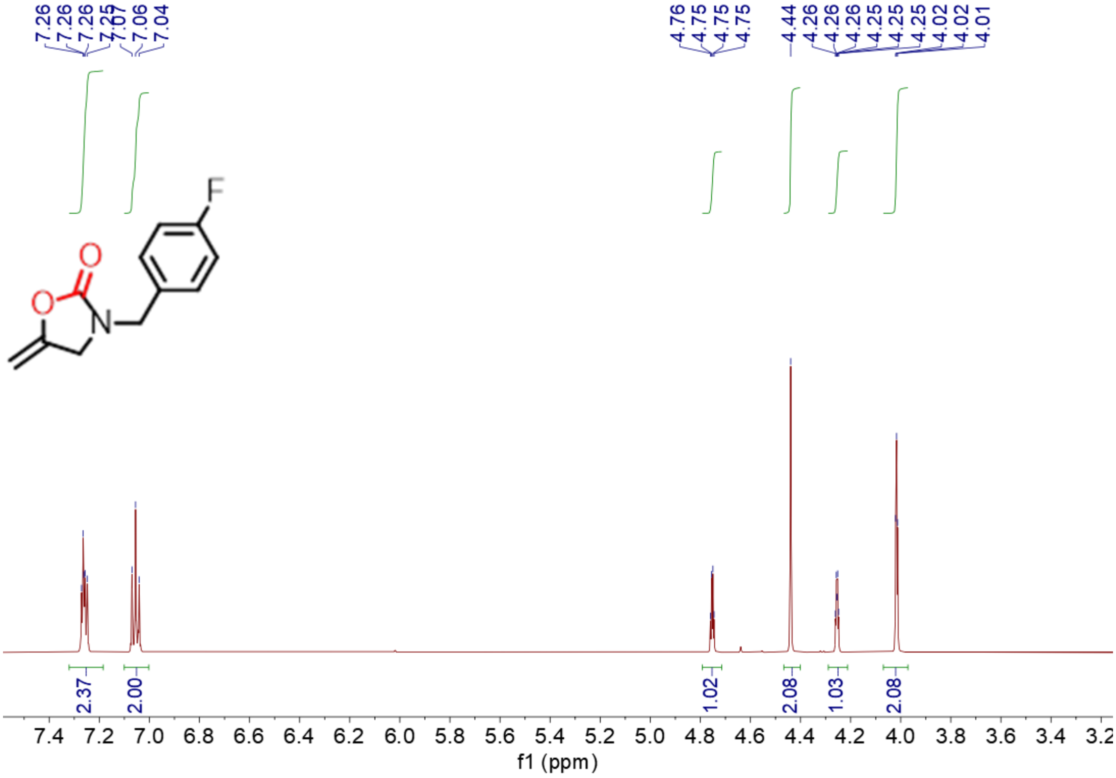
7.26
7.26
7.26
7.25
7.07
7.06
7.04
4.76
4.75
4.75
4.75
4.44
4.26
4.26
4.26
4.25
4.25
4.25
4.02
4.02
4.01
F
O
O
N
2.37
2.00
1.02
2.08
1.03
2.08
f1 (ppm)

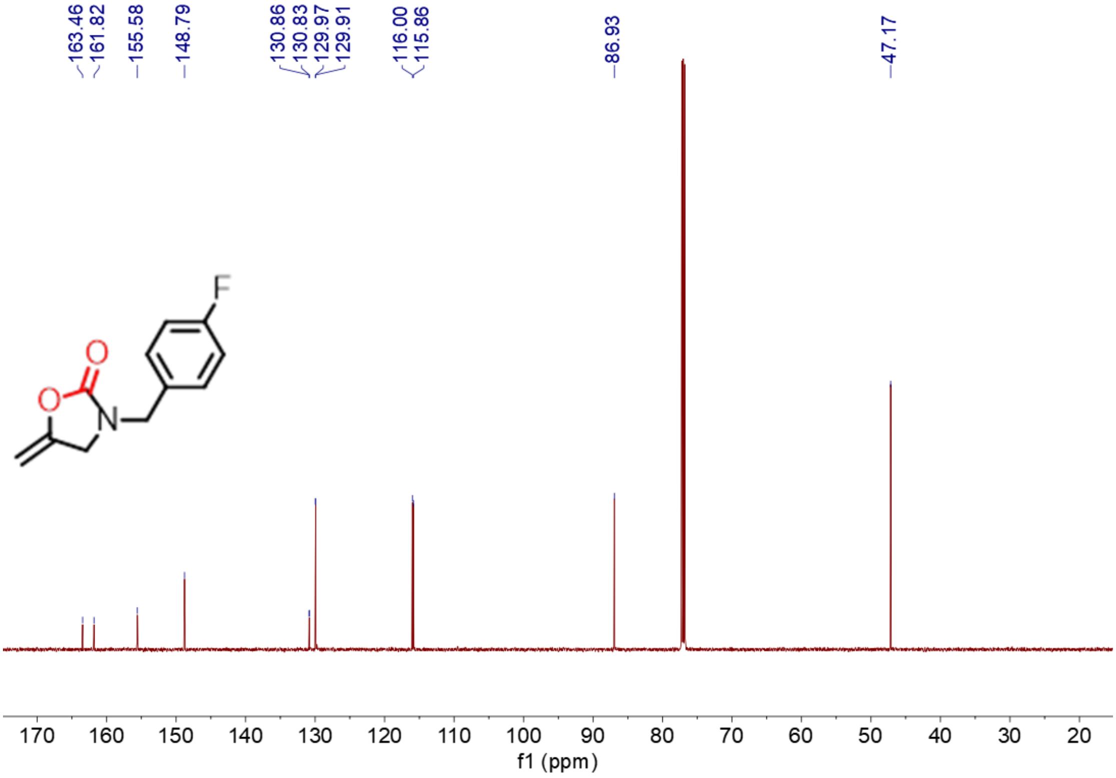
163.46
161.82
155.58
148.79
130.86
130.83
129.97
129.91
116.00
115.86
86.93
47.17
F
O
O
N
f1 (ppm)

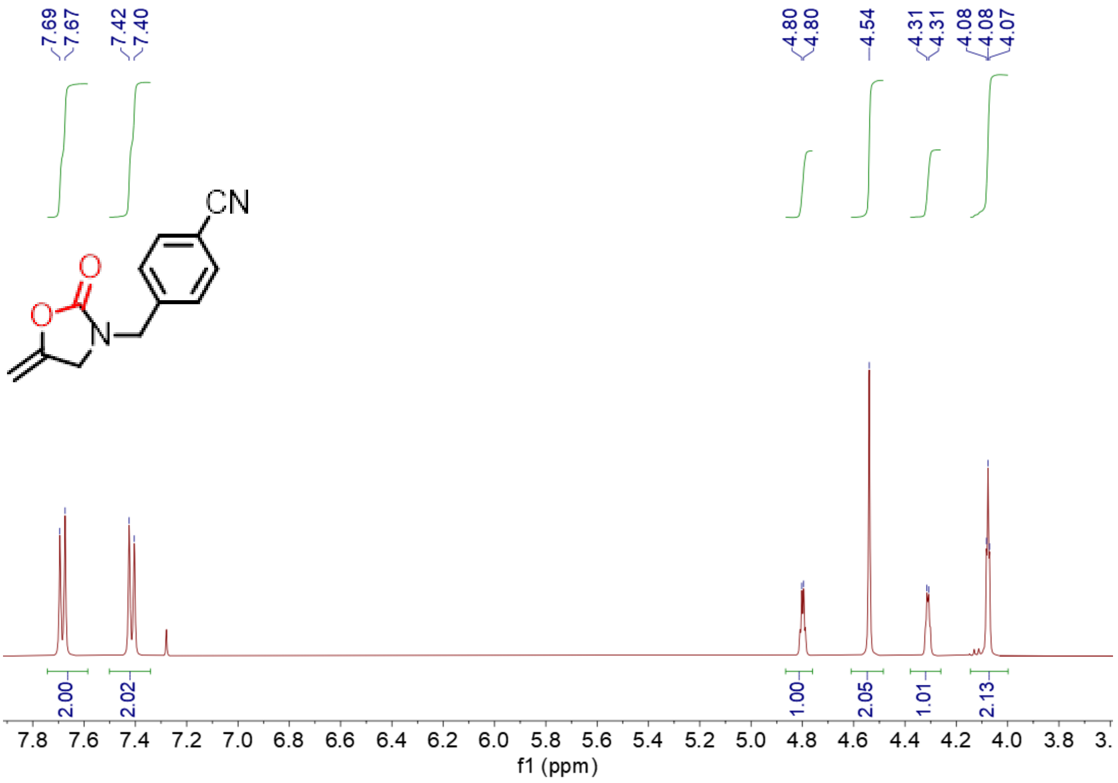
7.69
7.67
7.42
7.40
4.80
4.80
4.54
4.31
4.31
4.08
4.08
4.07
CN
O
O
N
2.00
2.02
1.00
2.05
1.01
2.13
7.8 7.6 7.4 7.2 7.0 6.8 6.6 6.4 6.2 6.0 5.8 5.6 5.4 5.2 5.0 4.8 4.6 4.4 4.2 4.0 3.8
f1 (ppm)

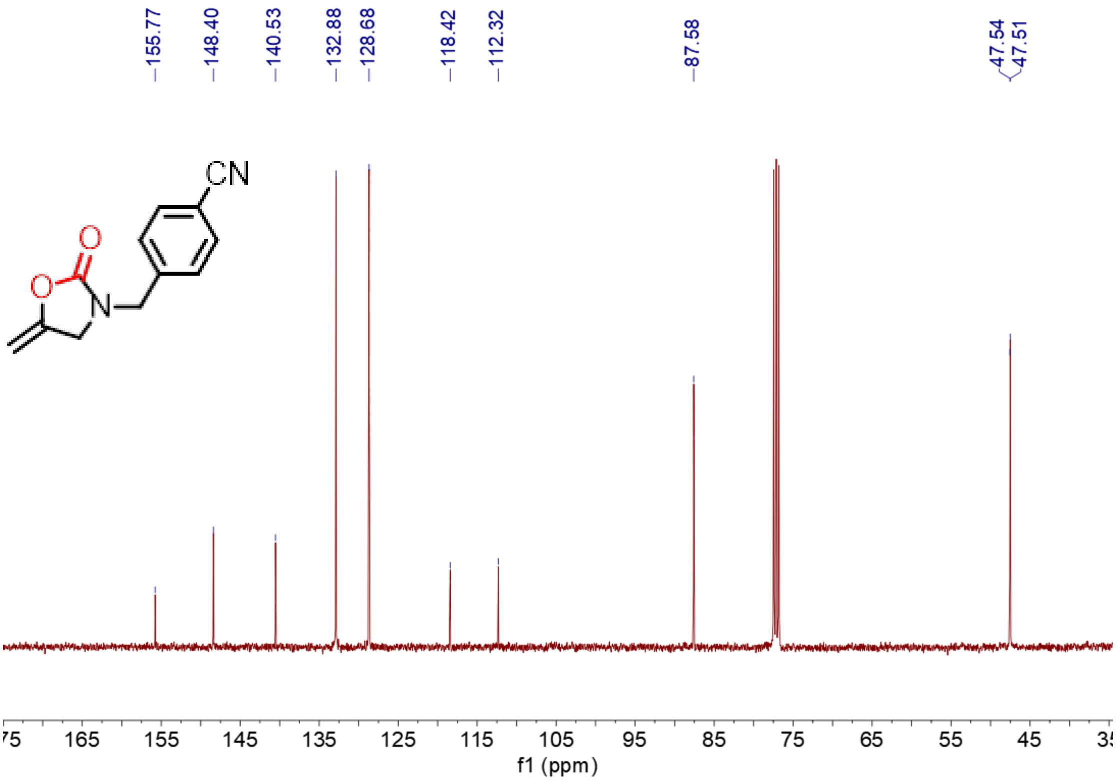
155.77
148.40
140.53
132.88
128.68
118.42
112.32
87.58
47.54
47.51
CN
O
O
N
165 155 145 135 125 115 105 95 85 75 65 55 45
f1 (ppm)

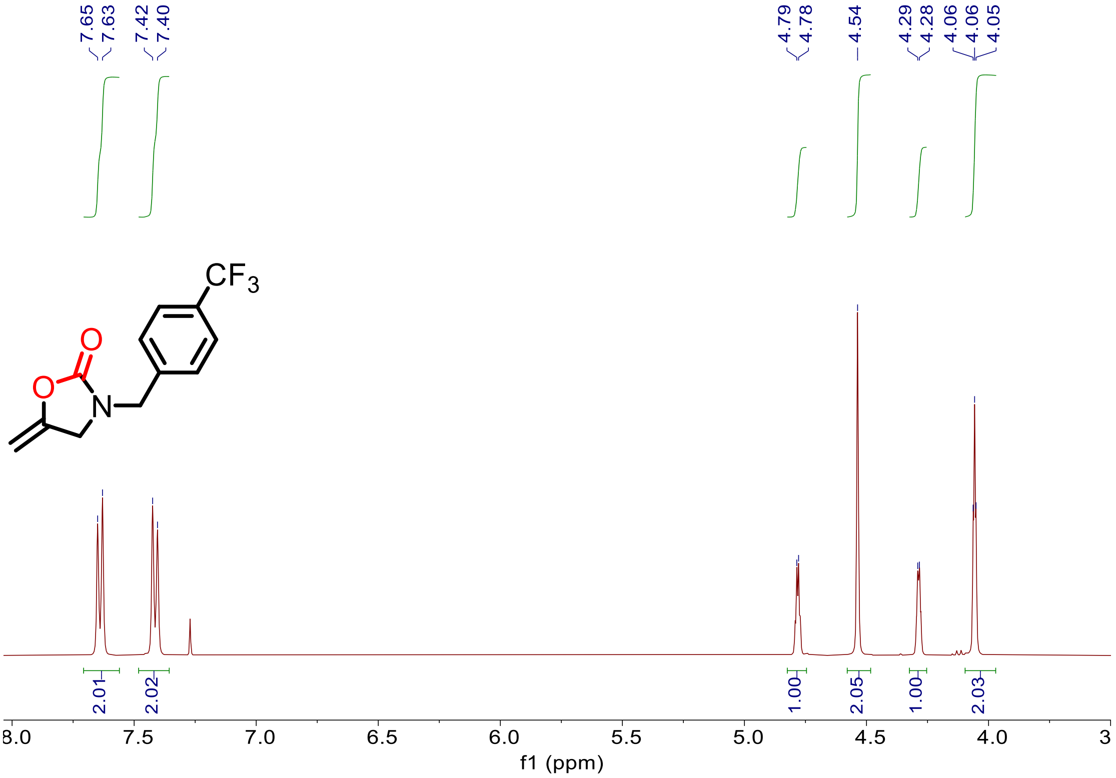
7.65
7.63
7.42
7.40
4.79
4.78
4.54
4.29
4.28
4.06
4.06
4.05
CF3
O
O
N
2.01
2.02
1.00
2.05
1.00
2.03
8.0 7.5 7.0 6.5 6.0 5.5 5.0 4.5 4.0 3
f1 (ppm)

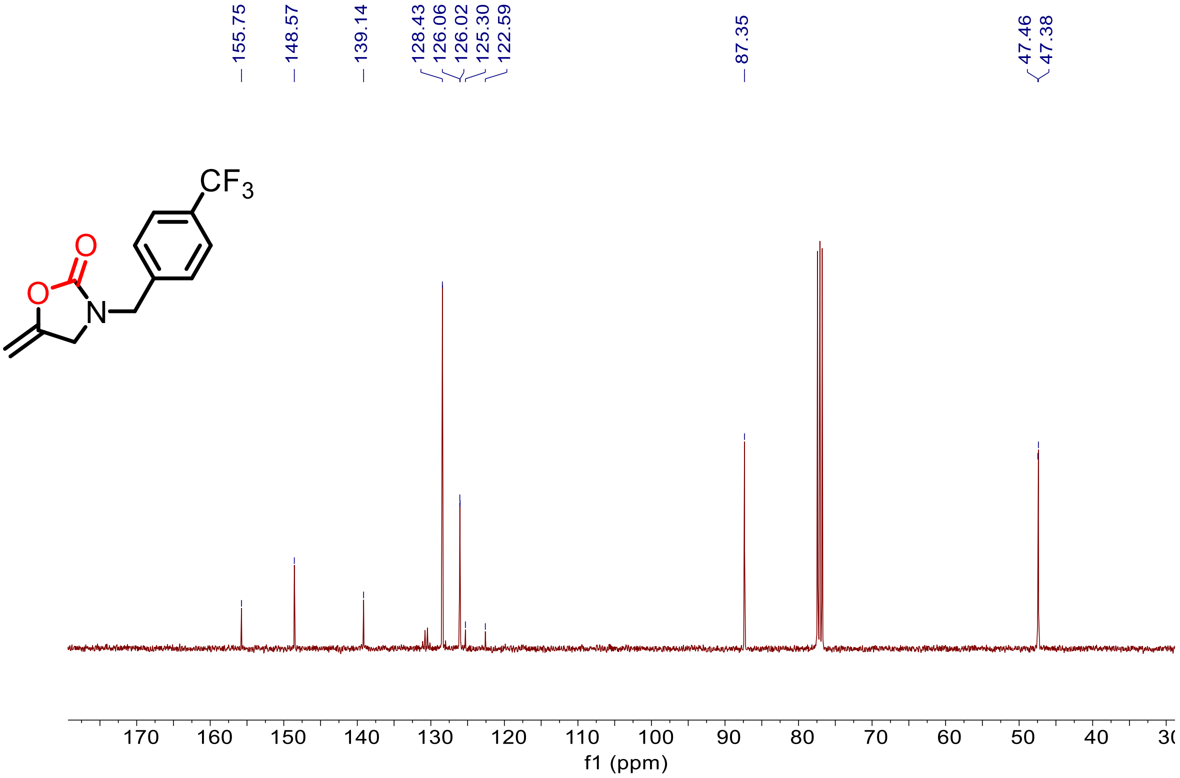
155.75
148.57
139.14
128.43
126.06
126.02
125.30
122.59
87.35
47.46
47.38
CF3
O
O
N
170 160 150 140 130 120 110 100 90 80 70 60 50 40 30
f1 (ppm)

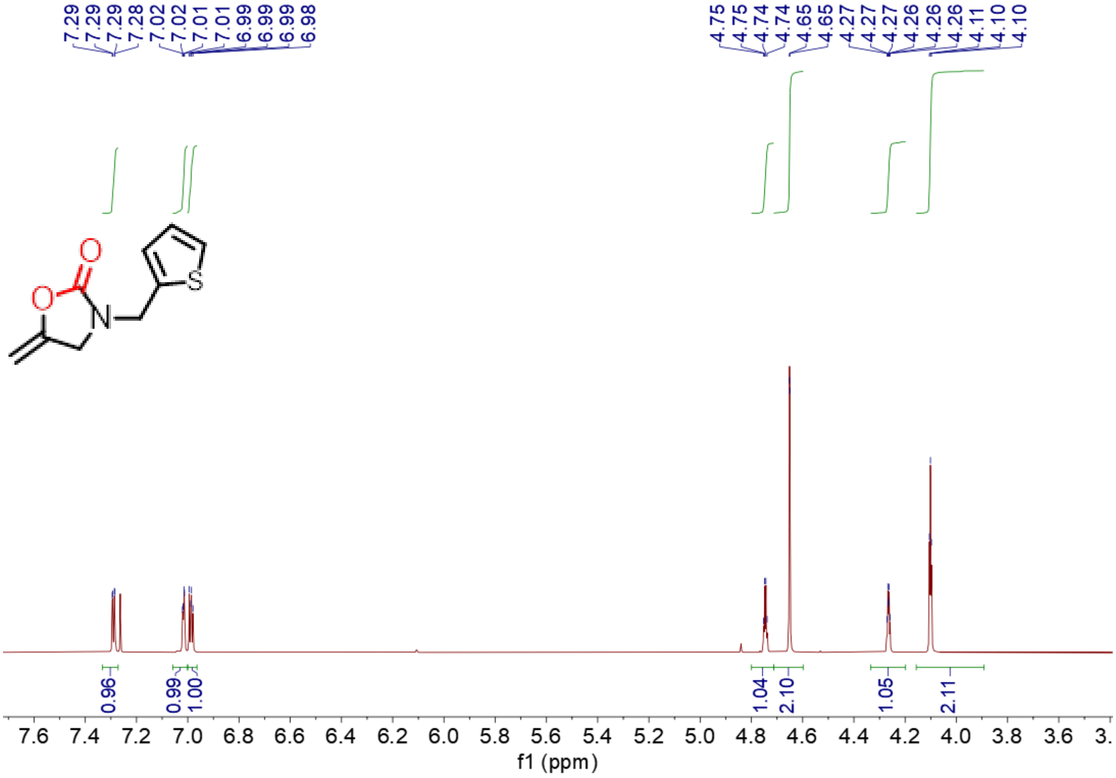
7.29 7.29 7.29 7.28 7.02 7.02 7.01 7.01 6.99 6.99 6.99 6.98
4.75 4.75 4.74 4.74 4.65 4.65 4.27 4.27 4.27 4.26 4.26 4.26 4.11 4.10 4.10
O
O
N
S
0.96
0.99
1.00
1.04
2.10
1.05
2.11
7.6 7.4 7.2 7.0 6.8 6.6 6.4 6.2 6.0 5.8 5.6 5.4 5.2 5.0 4.8 4.6 4.4 4.2 4.0 3.8 3.6 3.
f1 (ppm)

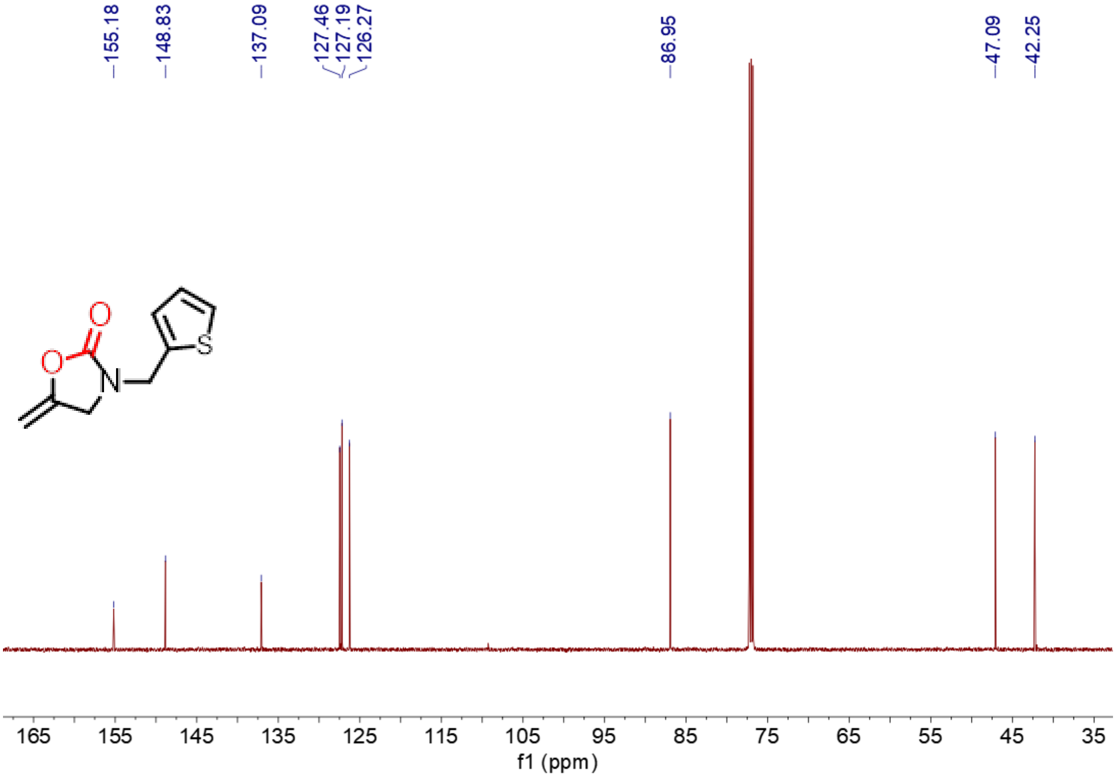
155.18
148.83
137.09
127.46 127.19 126.27
86.95
47.09
42.25
O
O
N
S
165 155 145 135 125 115 105 95 85 75 65 55 45 35
f1 (ppm)

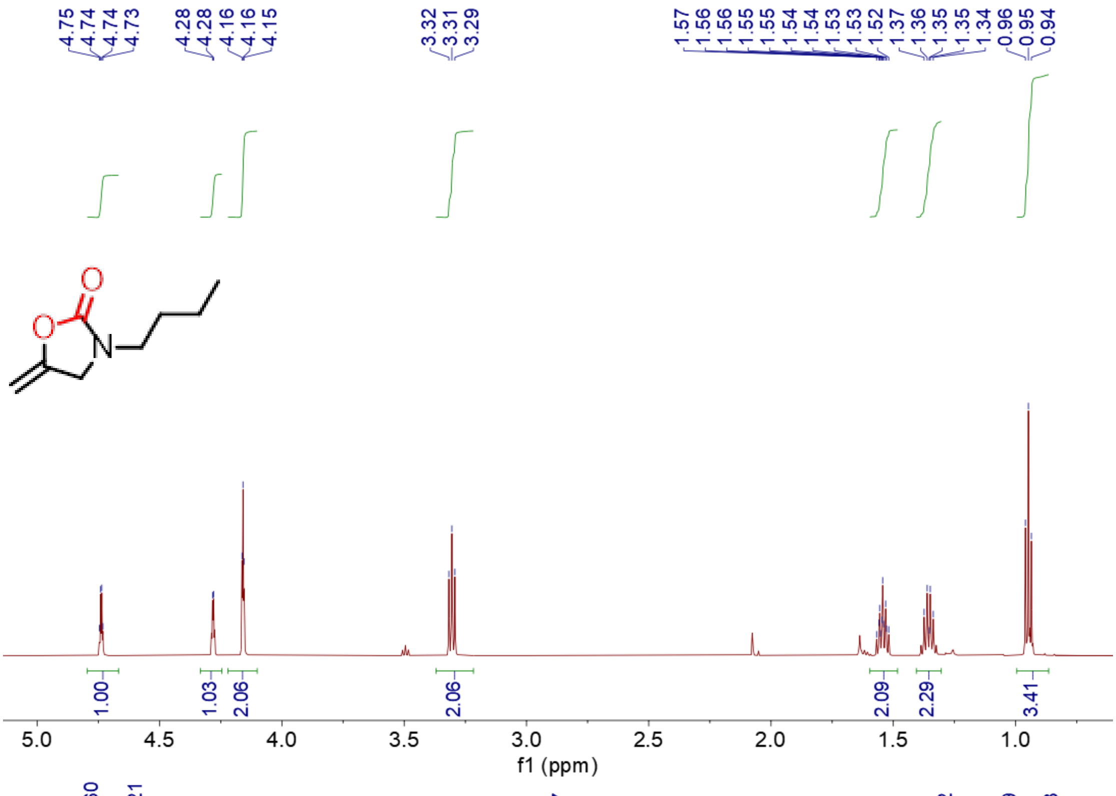

4.75
4.74
4.74
4.73
4.28
4.28
4.16
4.16
4.15
3.32
3.31
3.29
1.57
1.56
1.56
1.55
1.55
1.54
1.54
1.53
1.53
1.52
1.37
1.36
1.35
1.35
1.34
0.96
0.95
0.94
O
O
N
1.00
1.03
2.06
2.06
2.09
2.29
3.41
5.0
4.5
4.0
3.5
3.0
2.5
2.0
1.5
1.0
f1 (ppm)

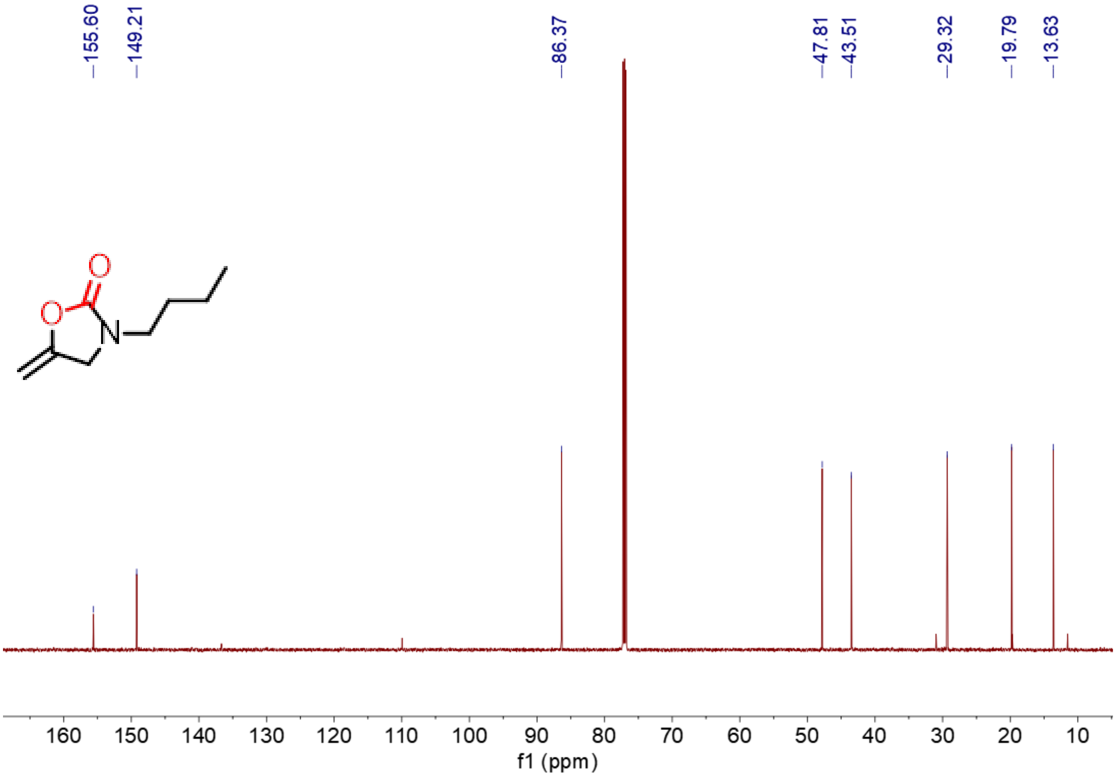

155.60
149.21
86.37
47.81
43.51
29.32
19.79
13.63
O
O
N
160
150
140
130
120
110
100
90
80
70
60
50
40
30
20
10
f1 (ppm)

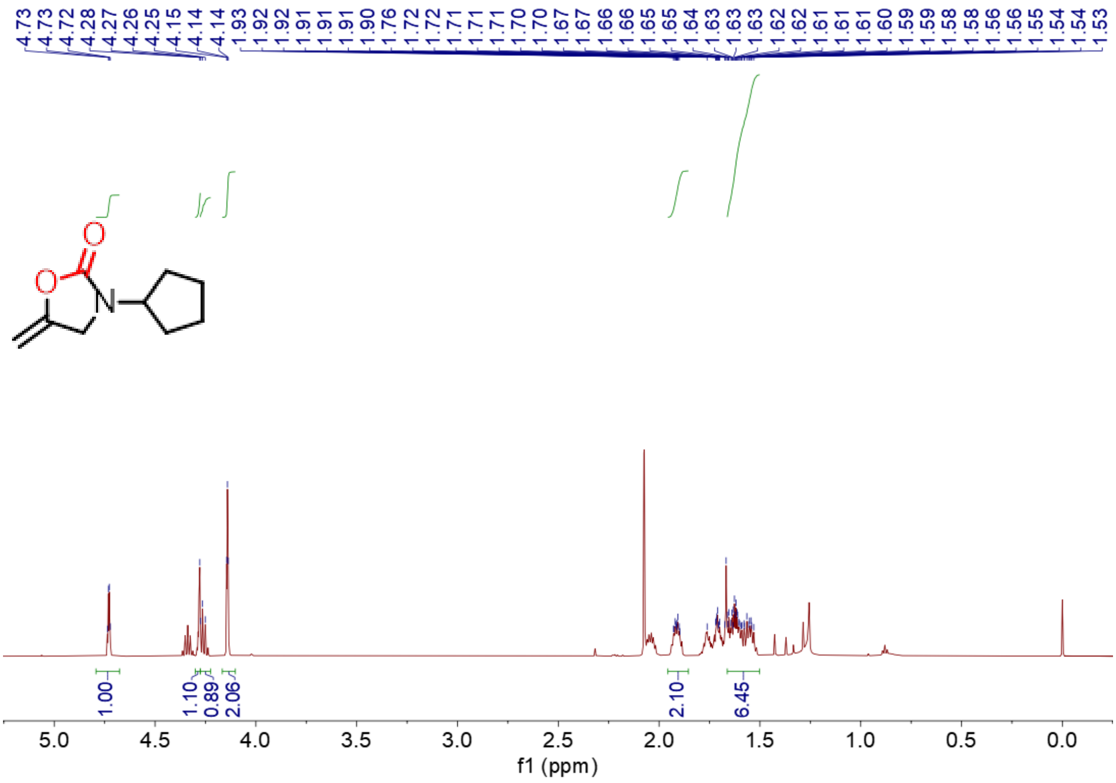
4.73 4.73 4.72 4.28 4.27 4.26 4.25 4.15 4.14 4.14 1.93 1.92 1.92 1.91 1.91 1.91 1.90 1.76 1.72 1.72 1.71 1.71 1.70 1.70 1.67 1.67 1.66 1.66 1.65 1.65 1.64 1.63 1.63 1.63 1.62 1.62 1.61 1.61 1.61 1.60 1.59 1.59 1.58 1.58 1.56 1.56 1.55 1.54 1.54 1.53
O
O
N
1.00
1.10
0.89
2.06
2.10
6.45
5.0 4.5 4.0 3.5 3.0 2.5 2.0 1.5 1.0 0.5 0.0
f1 (ppm)

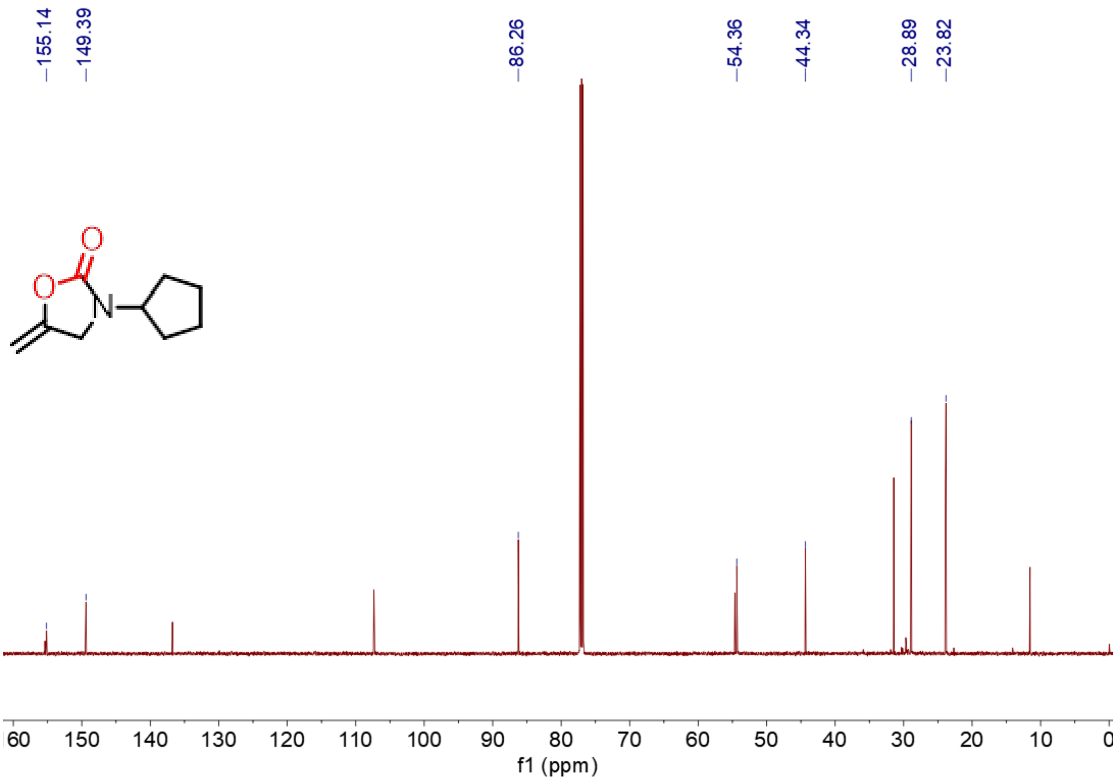
155.14
149.39
86.26
54.36
44.34
28.89
23.82
O
O
N
60 150 140 130 120 110 100 90 80 70 60 50 40 30 20 10 0
f1 (ppm)

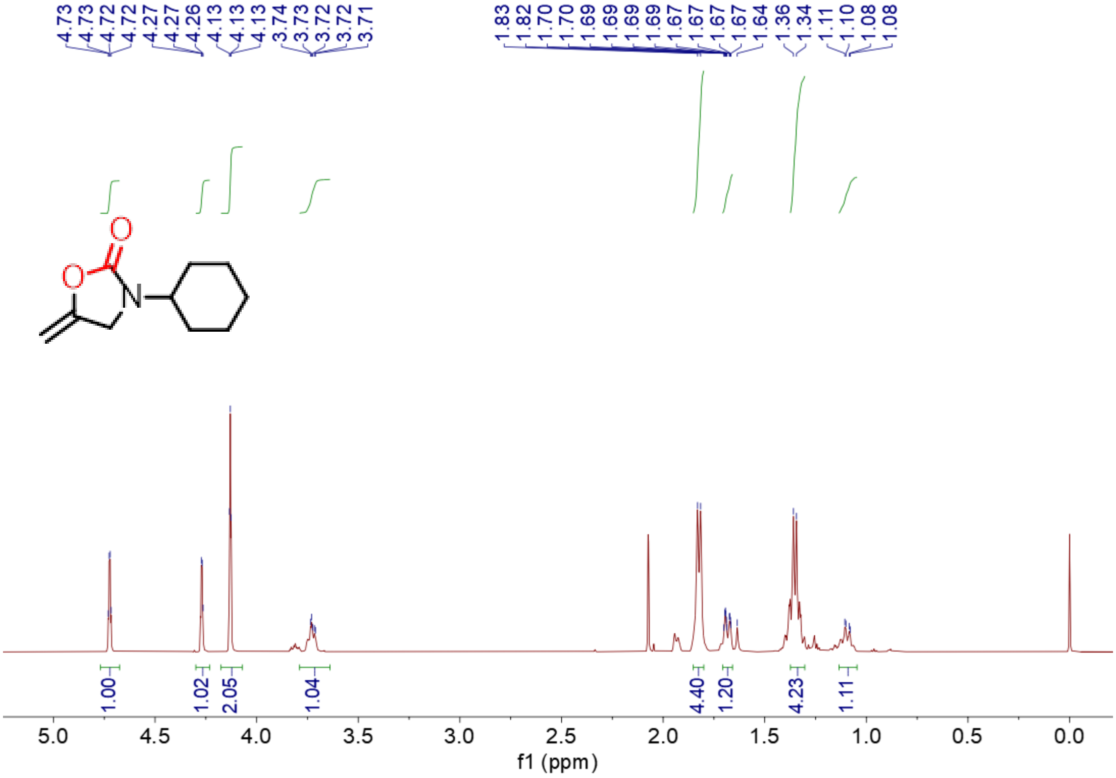
4.73
4.73
4.72
4.72
4.27
4.27
4.26
4.13
4.13
4.13
3.74
3.73
3.72
3.72
3.71
1.83
1.82
1.70
1.70
1.69
1.69
1.69
1.67
1.67
1.67
1.64
1.36
1.34
1.11
1.10
1.08
1.08
O
O
N
1.00
1.02
2.05
1.04
4.40
1.20
4.23
1.11
5.0
4.5
4.0
3.5
3.0
2.5
2.0
1.5
1.0
0.5
0.0
f1 (ppm)

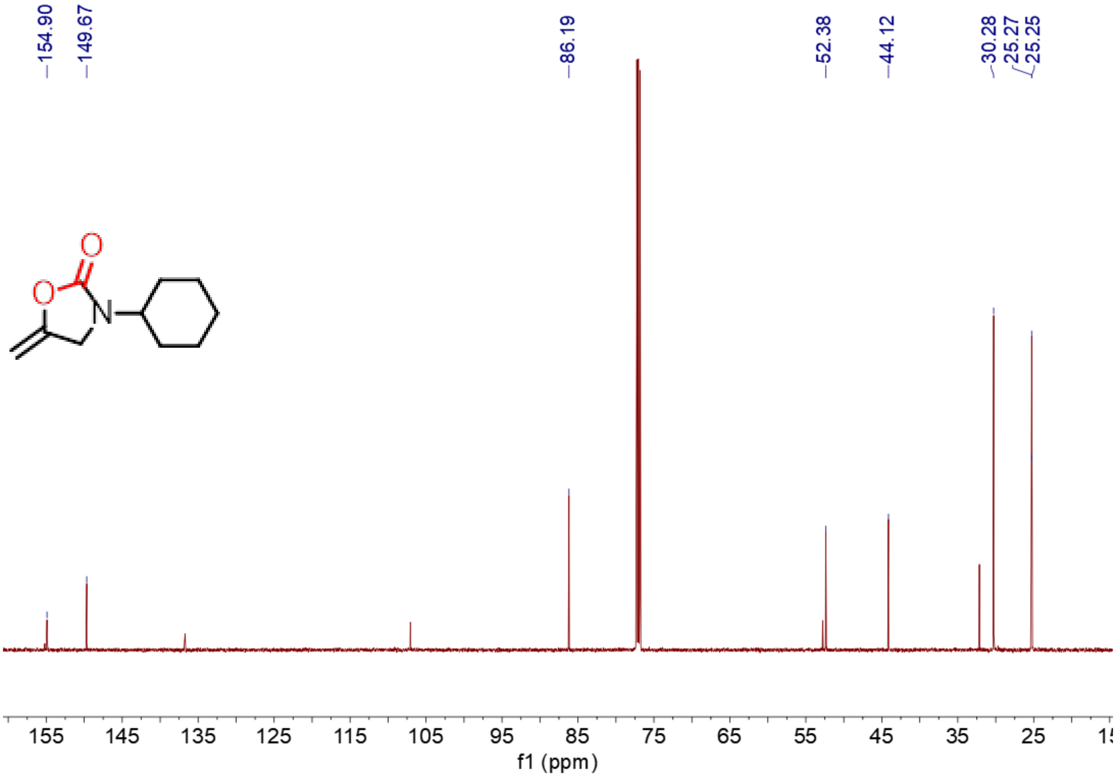
154.90
149.67
86.19
52.38
44.12
30.28
25.27
25.25
O
O
N
155
145
135
125
115
105
95
85
75
65
55
45
35
25
f1 (ppm)

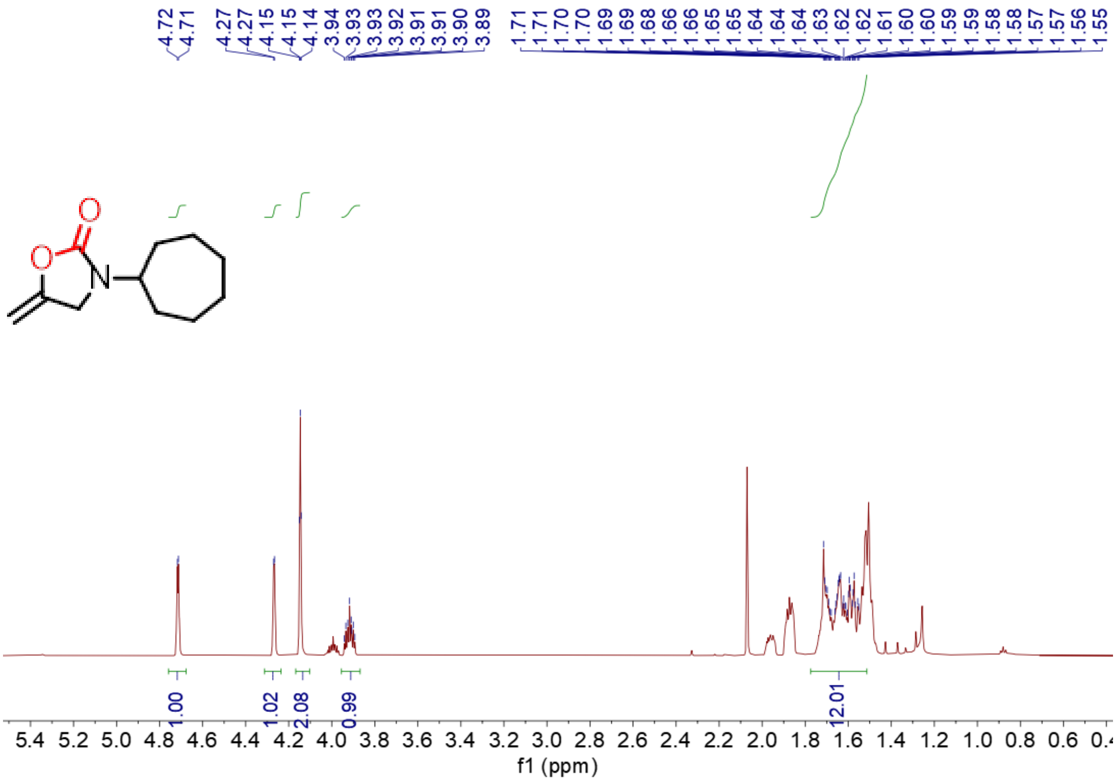

4.72
4.71
4.27
4.27
4.15
4.15
4.14
3.94
3.93
3.93
3.92
3.91
3.91
3.90
3.89
1.71
1.71
1.70
1.70
1.69
1.69
1.68
1.66
1.66
1.65
1.65
1.64
1.64
1.64
1.63
1.62
1.62
1.61
1.60
1.60
1.59
1.59
1.58
1.58
1.57
1.57
1.56
1.55
O
O
N
1.00
1.02
2.08
0.99
12.01
5.4 5.2 5.0 4.8 4.6 4.4 4.2 4.0 3.8 3.6 3.4 3.2 3.0 2.8 2.6 2.4 2.2 2.0 1.8 1.6 1.4 1.2 1.0 0.8 0.6
f1 (ppm)

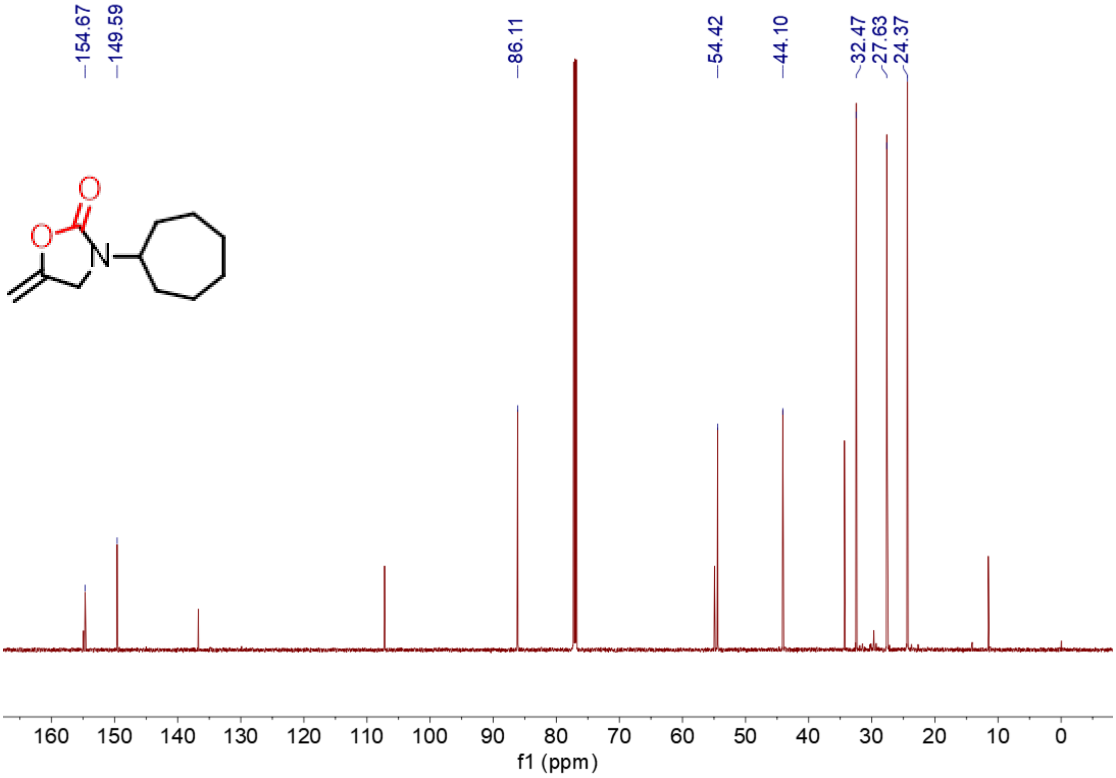

154.67
149.59
86.11
54.42
44.10
32.47
27.63
24.37
O
O
N
160 150 140 130 120 110 100 90 80 70 60 50 40 30 20 10 0
f1 (ppm)

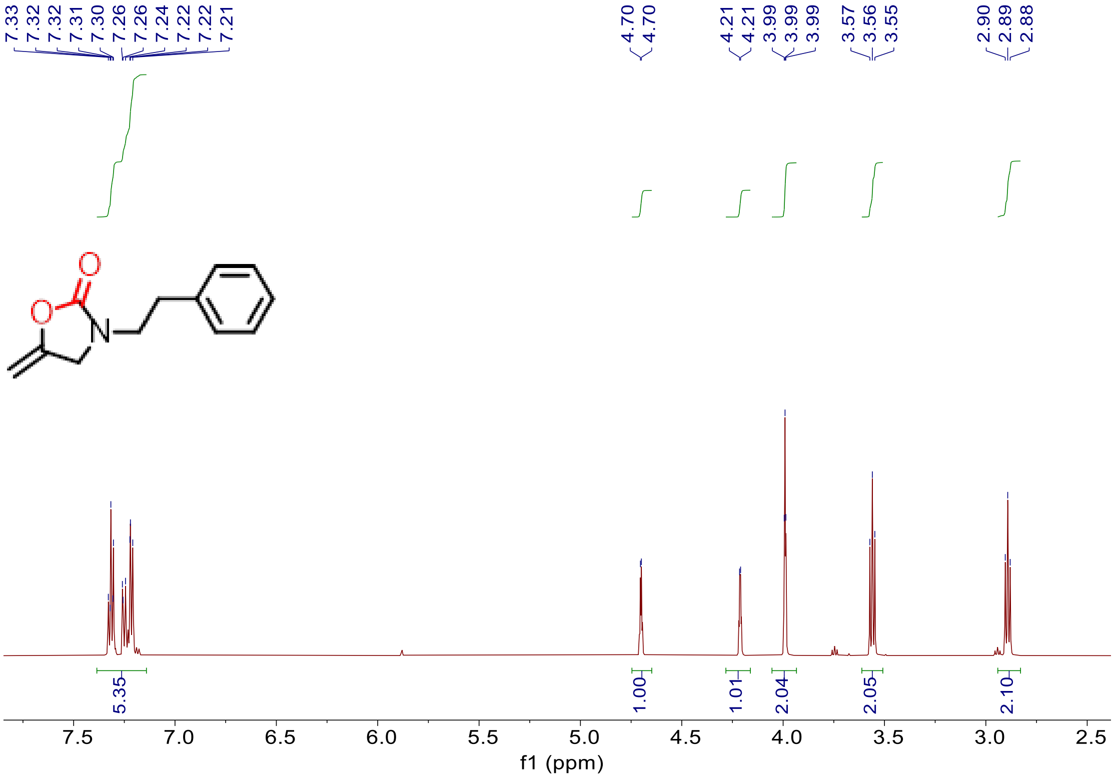
7.33 7.32 7.32 7.31 7.30 7.26 7.26 7.24 7.22 7.22 7.21
4.70 4.70
4.21 4.21 3.99 3.99 3.99
3.57 3.56 3.55
2.90 2.89 2.88
O
O
N
5.35
1.00
1.01
2.04
2.05
2.10
7.5 7.0 6.5 6.0 5.5 5.0 4.5 4.0 3.5 3.0 2.5
f1 (ppm)

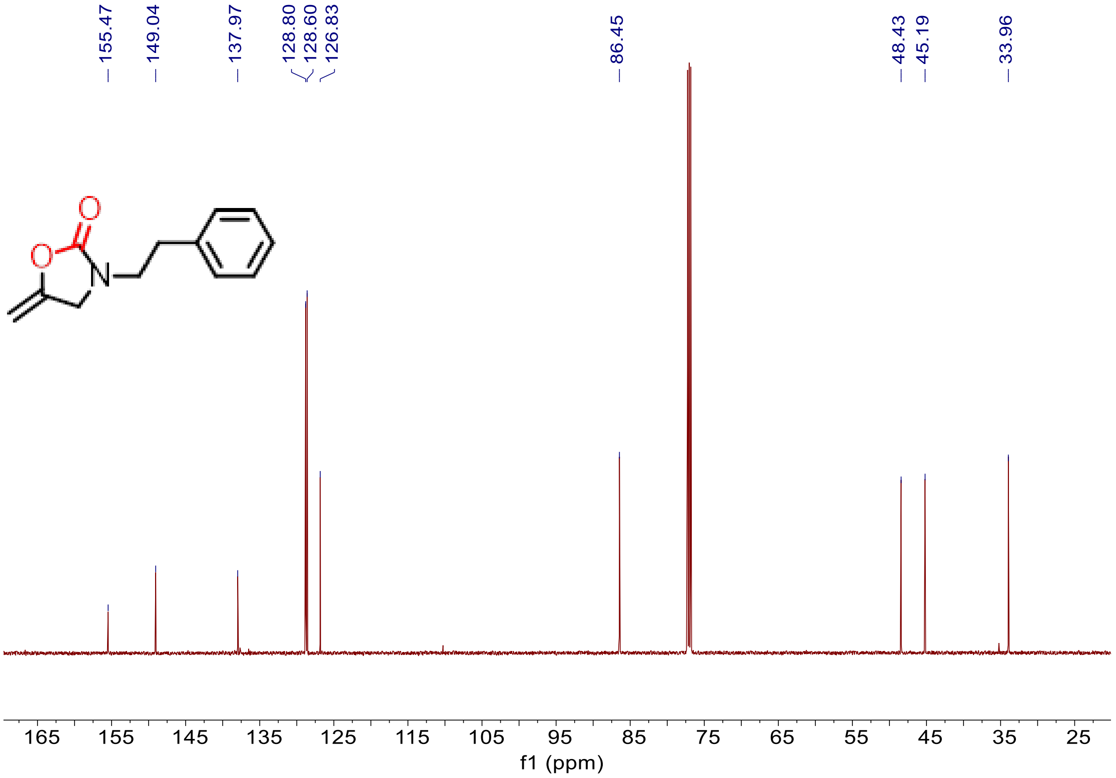
155.47
149.04
137.97
128.80 128.60 126.83
86.45
48.43 45.19
33.96
O
O
N
165 155 145 135 125 115 105 95 85 75 65 55 45 35 25
f1 (ppm)

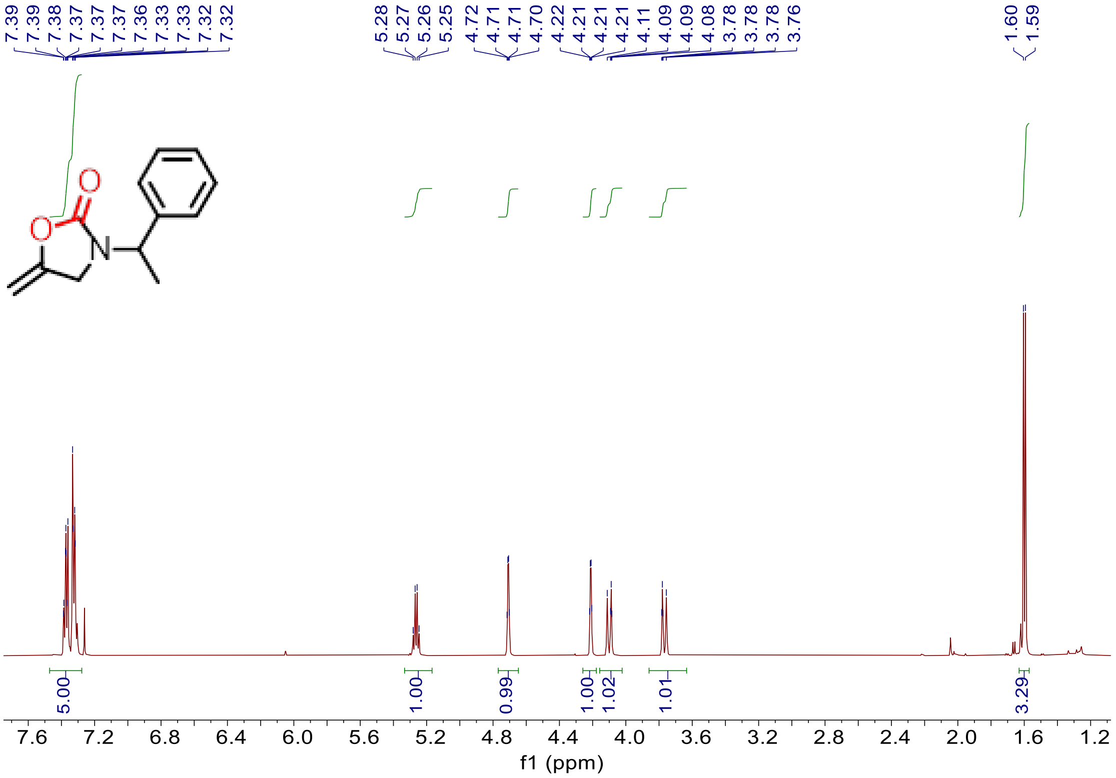
7.39
7.39
7.38
7.37
7.37
7.37
7.36
7.33
7.33
7.32
7.32
5.28
5.27
5.26
5.25
4.72
4.71
4.71
4.70
4.22
4.21
4.21
4.21
4.11
4.09
4.09
4.08
3.78
3.78
3.78
3.76
1.60
1.59
5.00
1.00
0.99
1.00
1.02
1.01
3.29
7.6 7.2 6.8 6.4 6.0 5.6 5.2 4.8 4.4 4.0 3.6 3.2 2.8 2.4 2.0 1.6 1.2
f1 (ppm)

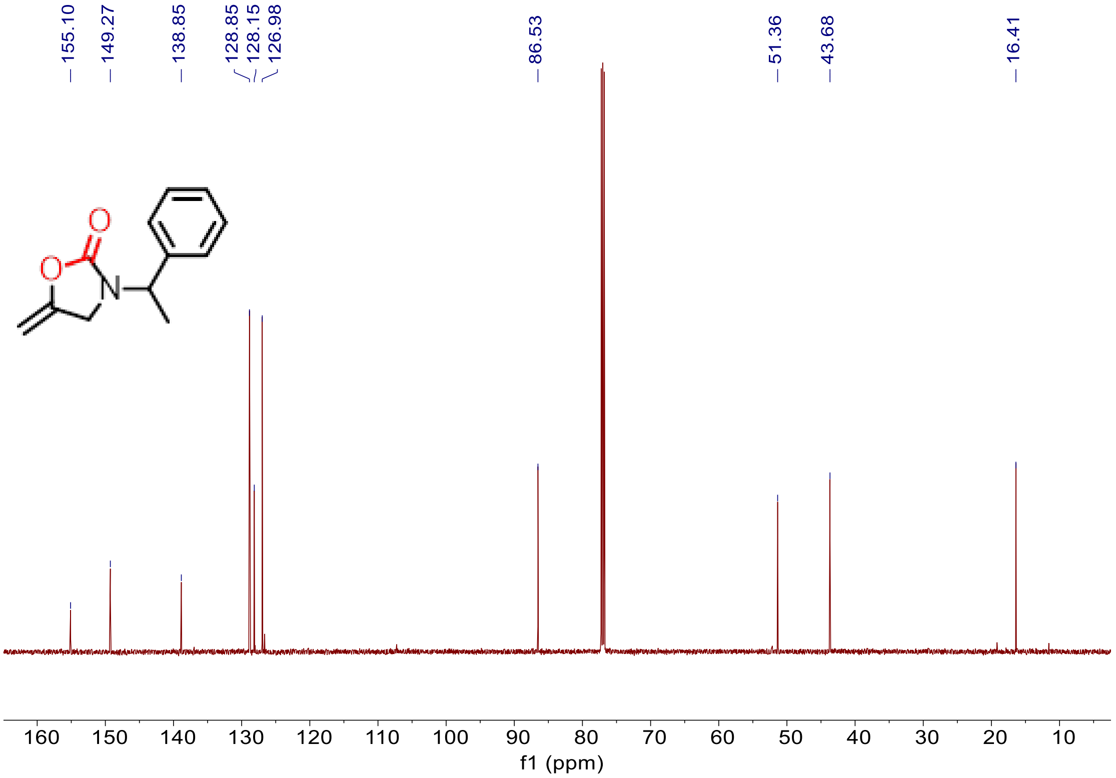
155.10
149.27
138.85
128.85
128.15
126.98
86.53
51.36
43.68
16.41
160 150 140 130 120 110 100 90 80 70 60 50 40 30 20 10
f1 (ppm)

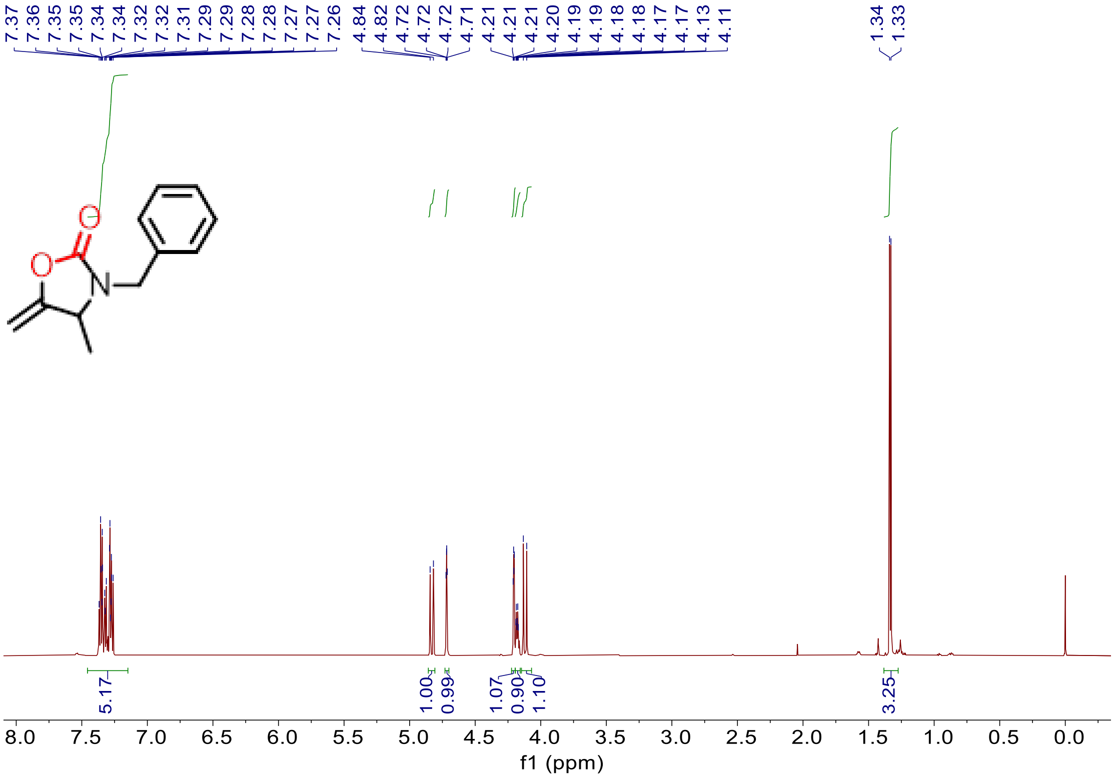
7.37
7.36
7.35
7.35
7.34
7.34
7.32
7.32
7.31
7.29
7.29
7.28
7.28
7.27
7.27
7.26
4.84
4.82
4.72
4.72
4.72
4.71
4.21
4.21
4.21
4.20
4.19
4.19
4.18
4.18
4.17
4.17
4.13
4.11
1.34
1.33
5.17
1.00
0.99
1.07
0.90
1.10
3.25
8.0
7.5
7.0
6.5
6.0
5.5
5.0
4.5
4.0
3.5
3.0
2.5
2.0
1.5
1.0
0.5
0.0
f1 (ppm)

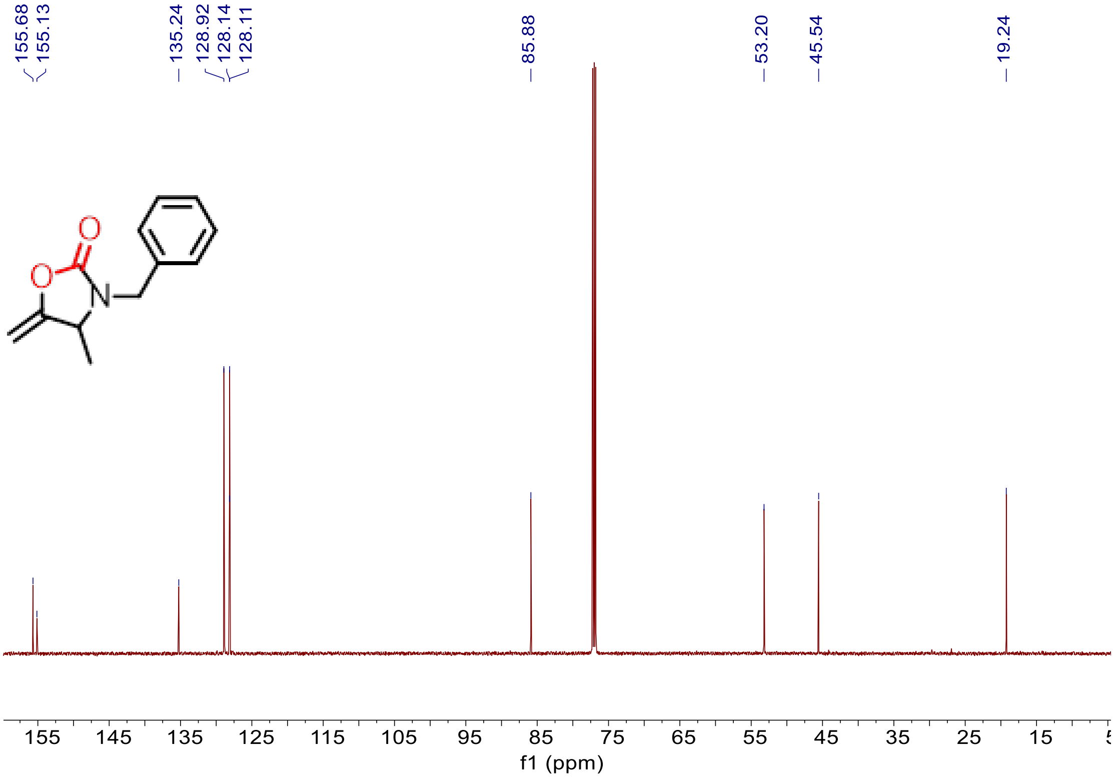
155.68
155.13
135.24
128.92
128.14
128.11
85.88
53.20
45.54
19.24
155
145
135
125
115
105
95
85
75
65
55
45
35
25
15
f1 (ppm)

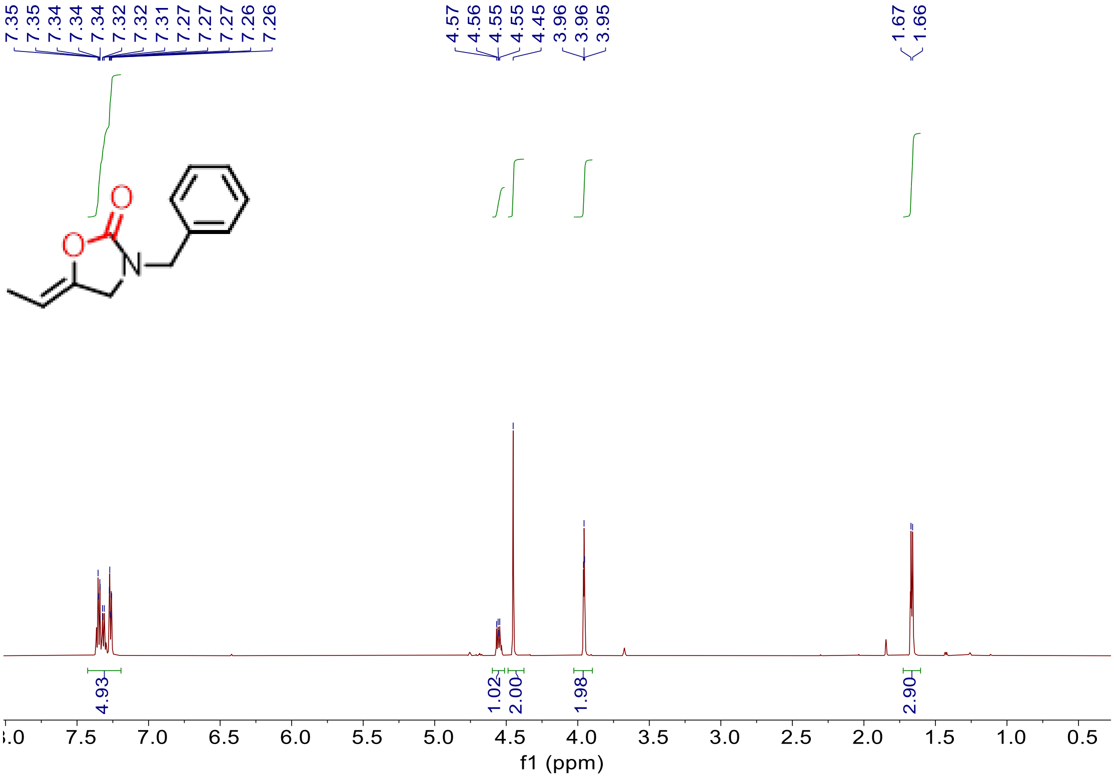
7.35
7.35
7.34
7.34
7.34
7.32
7.32
7.31
7.27
7.27
7.27
7.26
7.26
4.57
4.56
4.55
4.55
4.45
3.96
3.96
3.95
1.67
1.66
4.93
1.02
2.00
1.98
2.90
f1 (ppm)

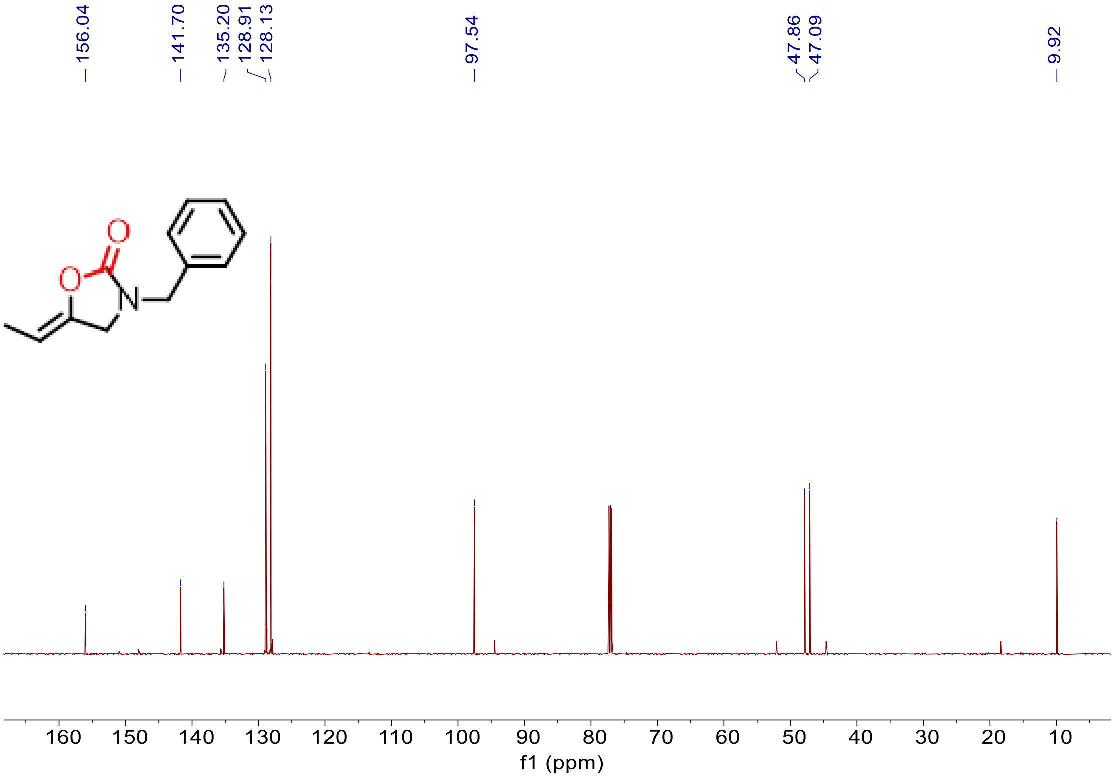
156.04
141.70
135.20
128.91
128.13
97.54
47.86
47.09
9.92
f1 (ppm)

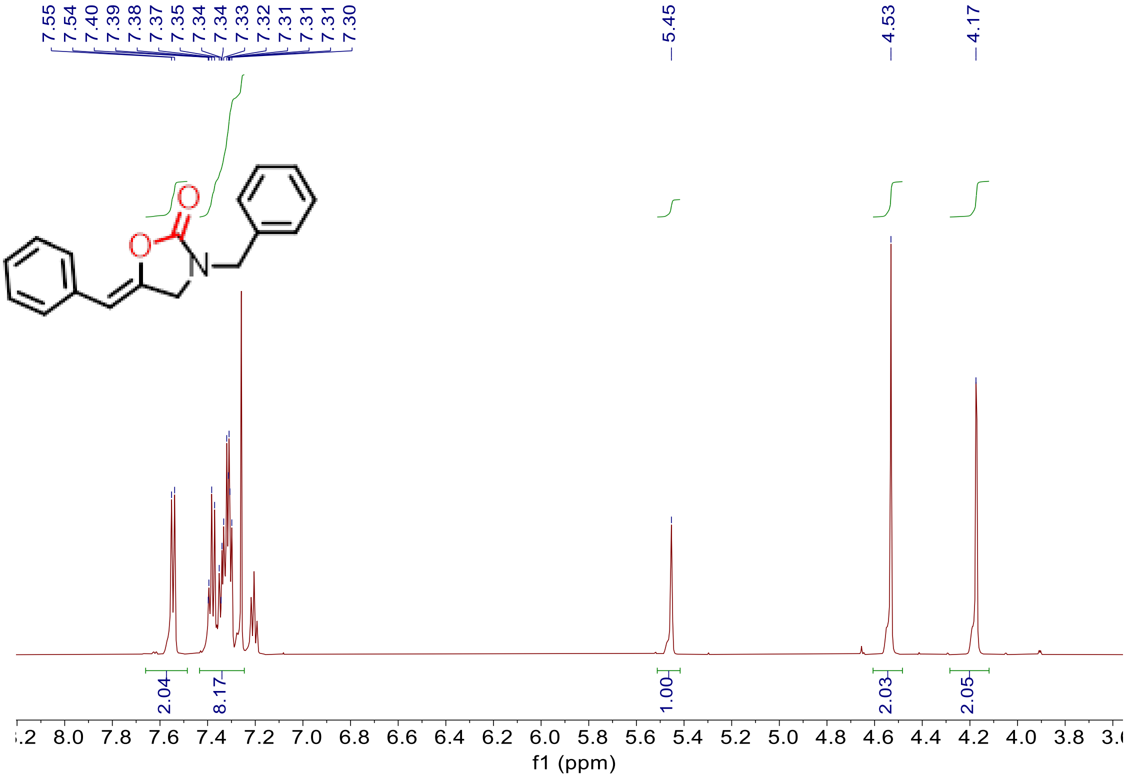
7.55 7.54 7.40 7.39 7.38 7.37 7.35 7.34 7.34 7.33 7.32 7.31 7.31 7.31 7.30
5.45
4.53
4.17
2.04
8.17
1.00
2.03
2.05
f1 (ppm)

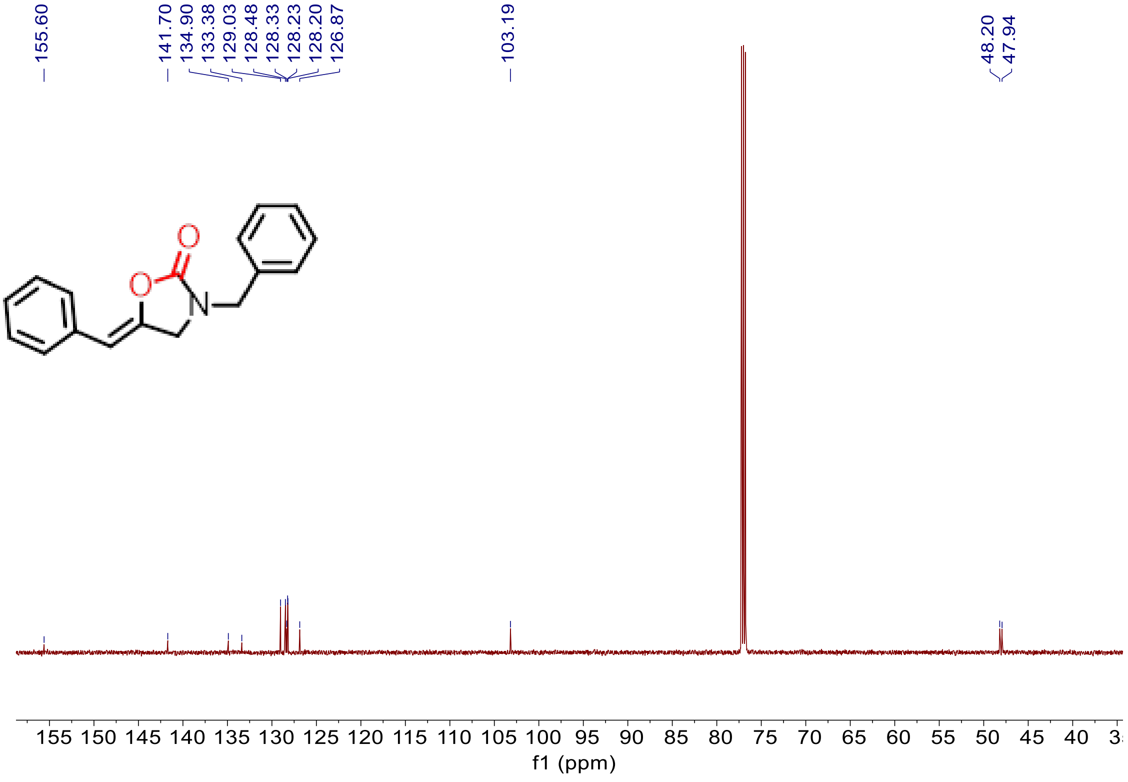
155.60
141.70 134.90 133.38 129.03 128.48 128.33 128.23 128.20 126.87
103.19
48.20 47.94
f1 (ppm)

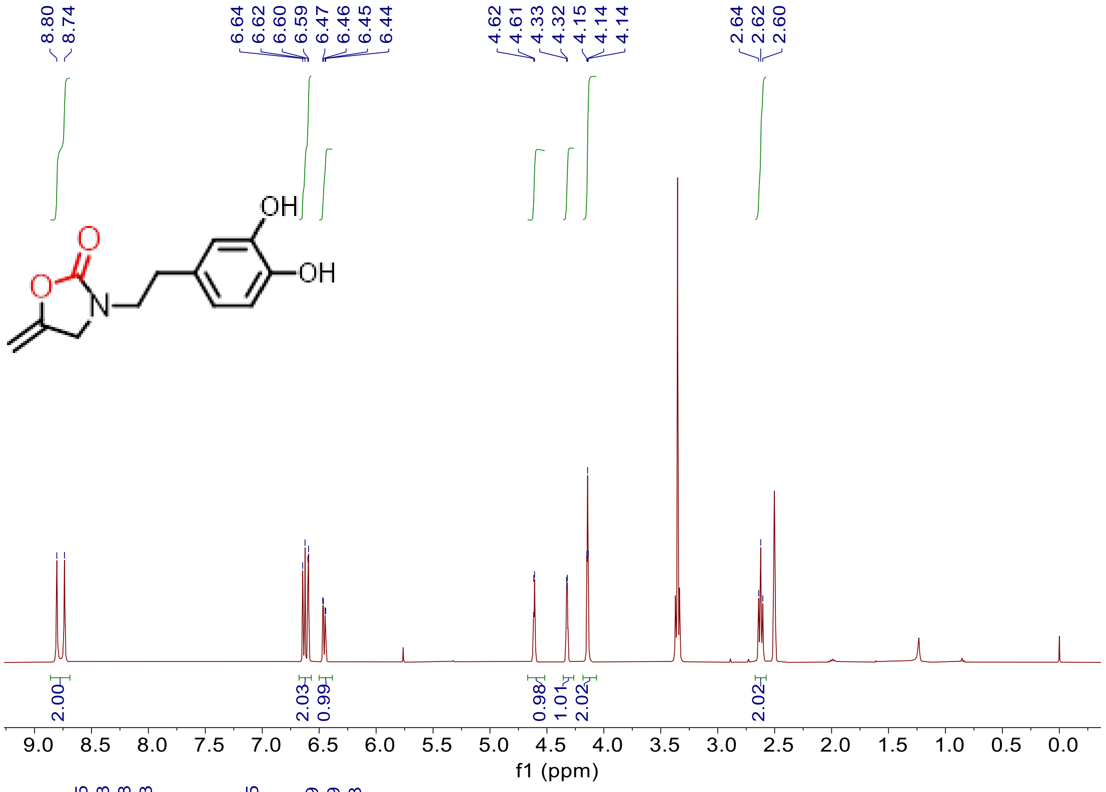
8.80
8.74
6.64
6.62
6.60
6.59
6.47
6.46
6.45
6.44
4.62
4.61
4.33
4.32
4.15
4.14
4.14
2.64
2.62
2.60
OH
OH
O
O
N
2.00
2.03
0.99
0.98
1.01
2.02
2.02
9.0 8.5 8.0 7.5 7.0 6.5 6.0 5.5 5.0 4.5 4.0 3.5 3.0 2.5 2.0 1.5 1.0 0.5 0.0
f1 (ppm)

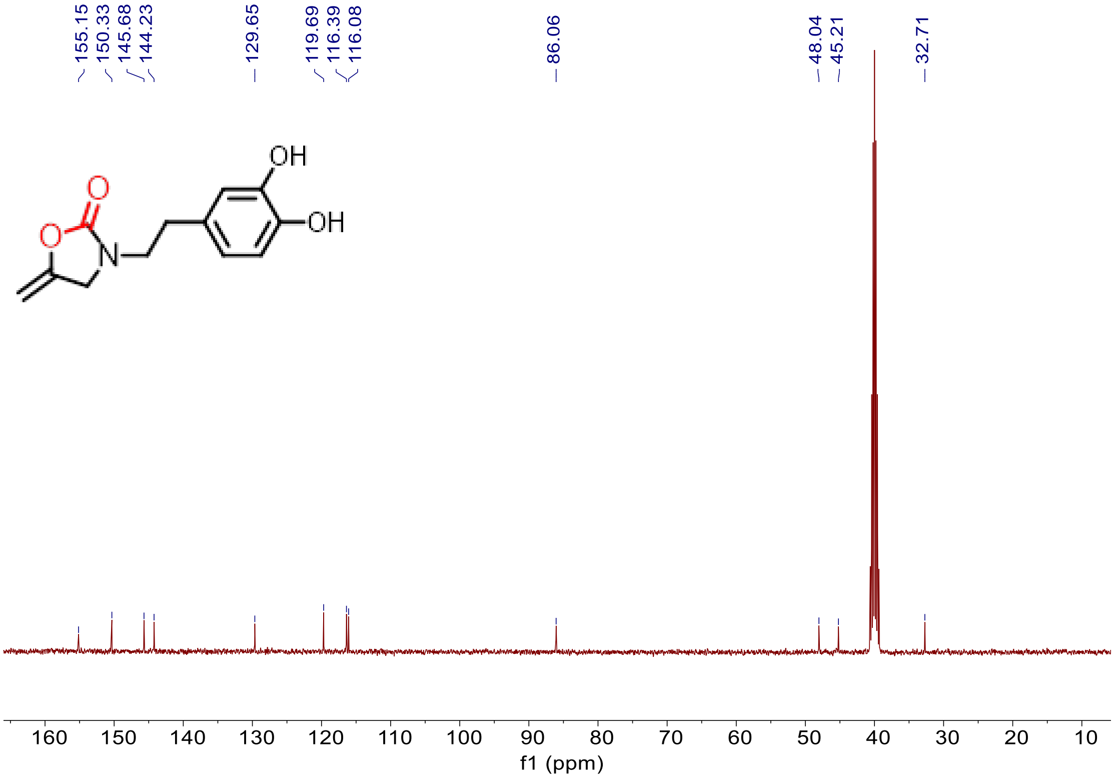
155.15
150.33
145.68
144.23
129.65
119.69
116.39
116.08
86.06
48.04
45.21
32.71
OH
OH
O
O
N
160 150 140 130 120 110 100 90 80 70 60 50 40 30 20 10
f1 (ppm)

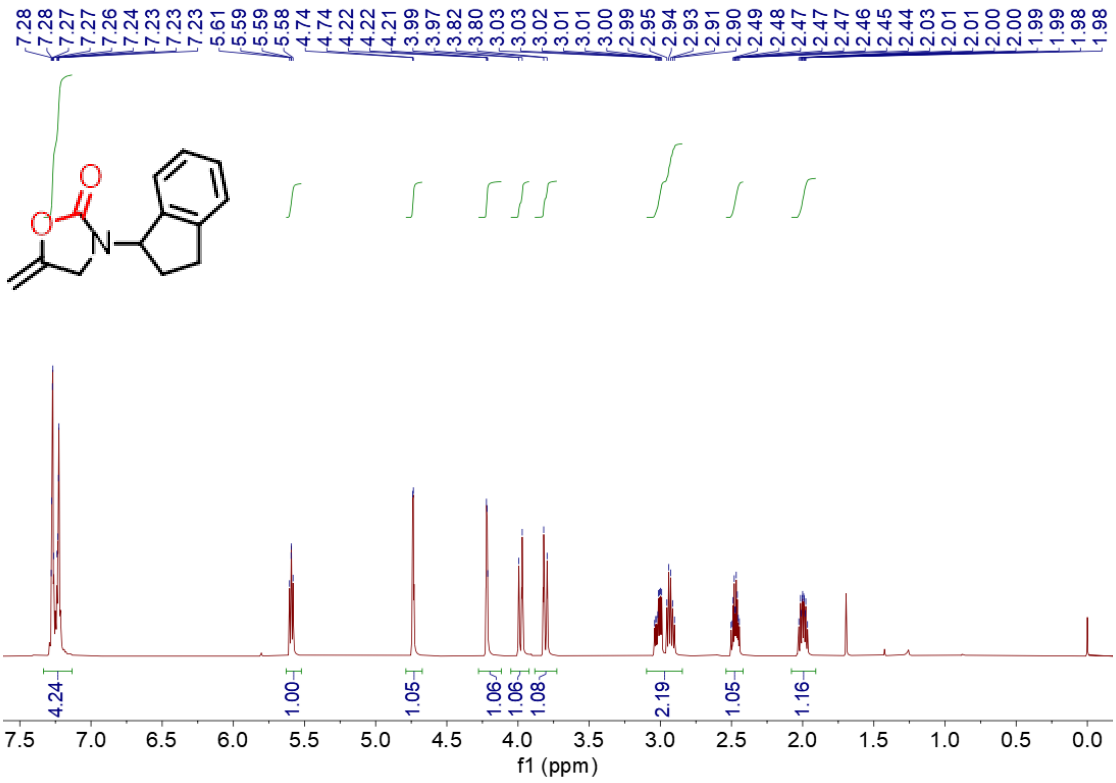
7.28 7.28 7.27 7.27 7.26 7.24 7.23 7.23 7.23 5.61 5.59 5.59 5.58 4.74 4.74 4.22 4.22 4.21 3.99 3.97 3.82 3.80 3.03 3.03 3.02 3.01 3.01 3.00 2.99 2.95 2.94 2.93 2.91 2.90 2.49 2.48 2.47 2.47 2.47 2.46 2.45 2.44 2.03 2.01 2.01 2.00 1.99 1.99 1.98 1.98
O
O
N
4.24 1.00 1.05 1.06 1.06 1.08 2.19 1.05 1.16
7.5 7.0 6.5 6.0 5.5 5.0 4.5 4.0 3.5 3.0 2.5 2.0 1.5 1.0 0.5 0.0
f1 (ppm)

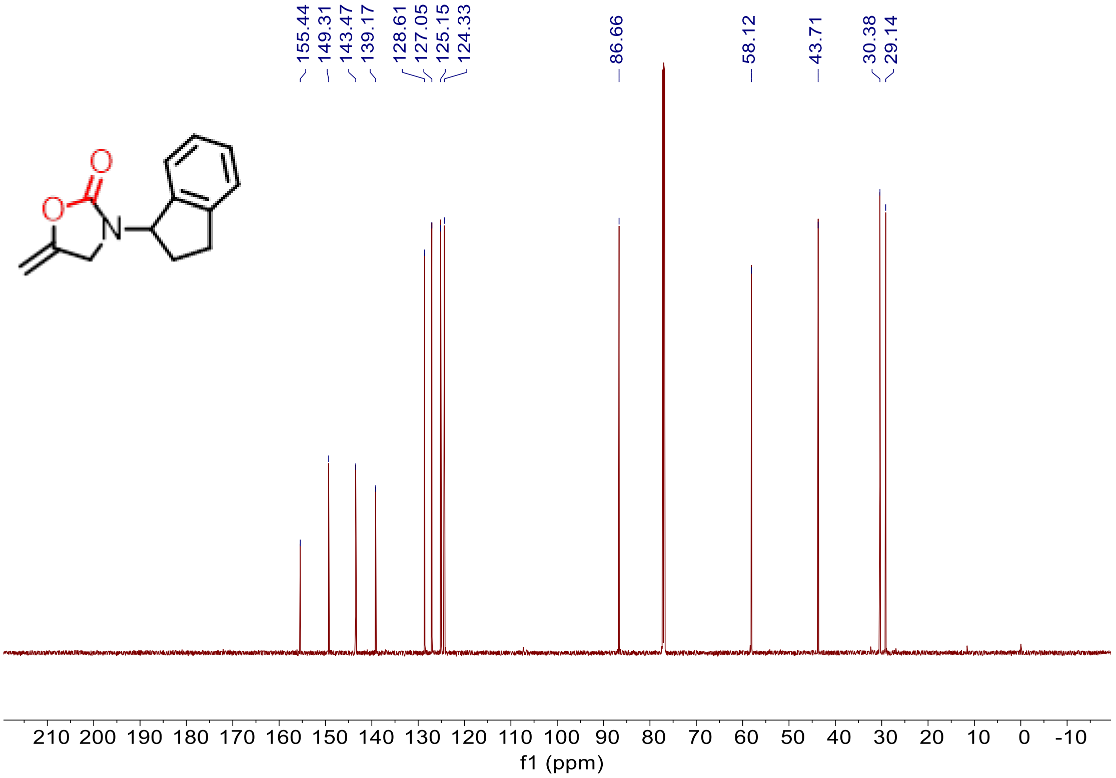
155.44 149.31 143.47 139.17 128.61 127.05 125.15 124.33
86.66
58.12
43.71
30.38 29.14
O
O
N
210 200 190 180 170 160 150 140 130 120 110 100 90 80 70 60 50 40 30 20 10 0 -10
f1 (ppm)

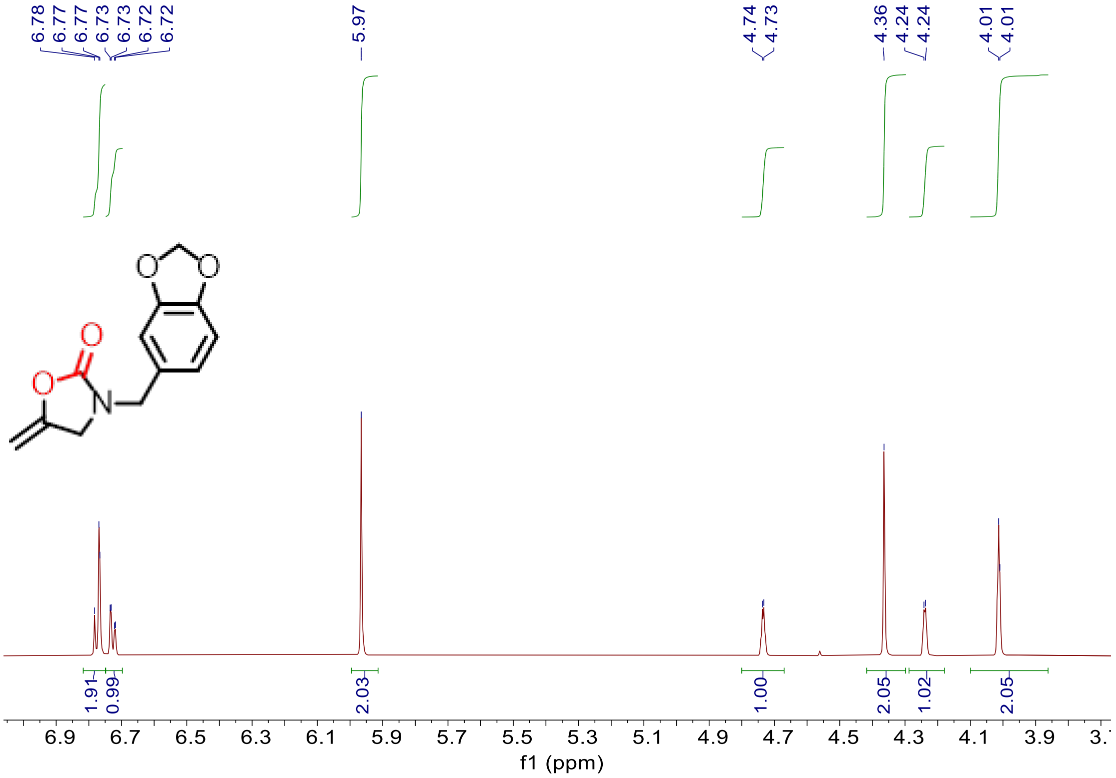
6.78 6.77 6.77 6.73 6.73 6.72 6.72
5.97
4.74 4.73
4.36 4.24 4.24
4.01 4.01
1.91 0.99 2.03 1.00 2.05 1.02 2.05
6.9 6.7 6.5 6.3 6.1 5.9 5.7 5.5 5.3 5.1 4.9 4.7 4.5 4.3 4.1 3.9
f1 (ppm)

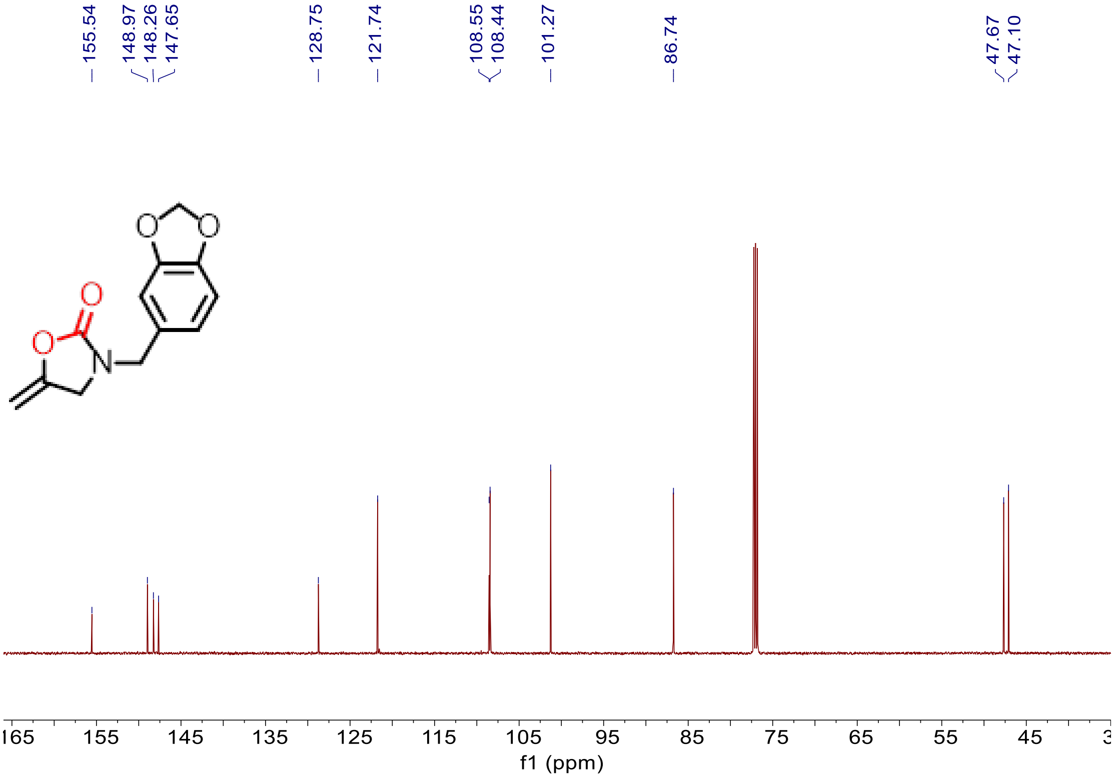
155.54
148.97 148.26 147.65
128.75
121.74
108.55 108.44
101.27
86.74
47.67 47.10
155 145 135 125 115 105 95 85 75 65 55 45
f1 (ppm)

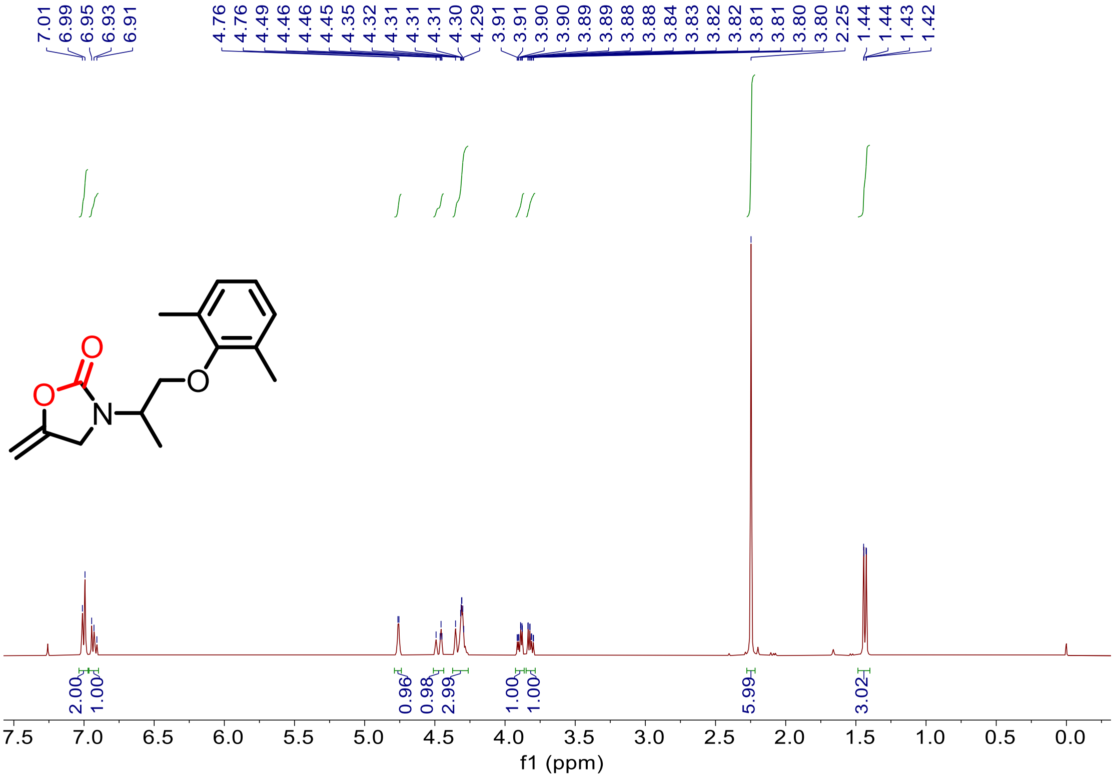
7.01 6.99 6.95 6.93 6.91
4.76 4.76 4.49 4.46 4.45 4.35 4.32 4.31 4.31 4.31 4.30 4.29 3.91 3.91 3.90 3.90 3.89 3.89 3.88 3.88 3.84 3.83 3.82 3.82 3.81 3.81 3.80 3.80 2.25 1.44 1.44 1.43 1.42
O
O
N
O
2.00 1.00 0.96 0.98 2.99 1.00 1.00 5.99 3.02
7.5 7.0 6.5 6.0 5.5 5.0 4.5 4.0 3.5 3.0 2.5 2.0 1.5 1.0 0.5 0.0
f1 (ppm)

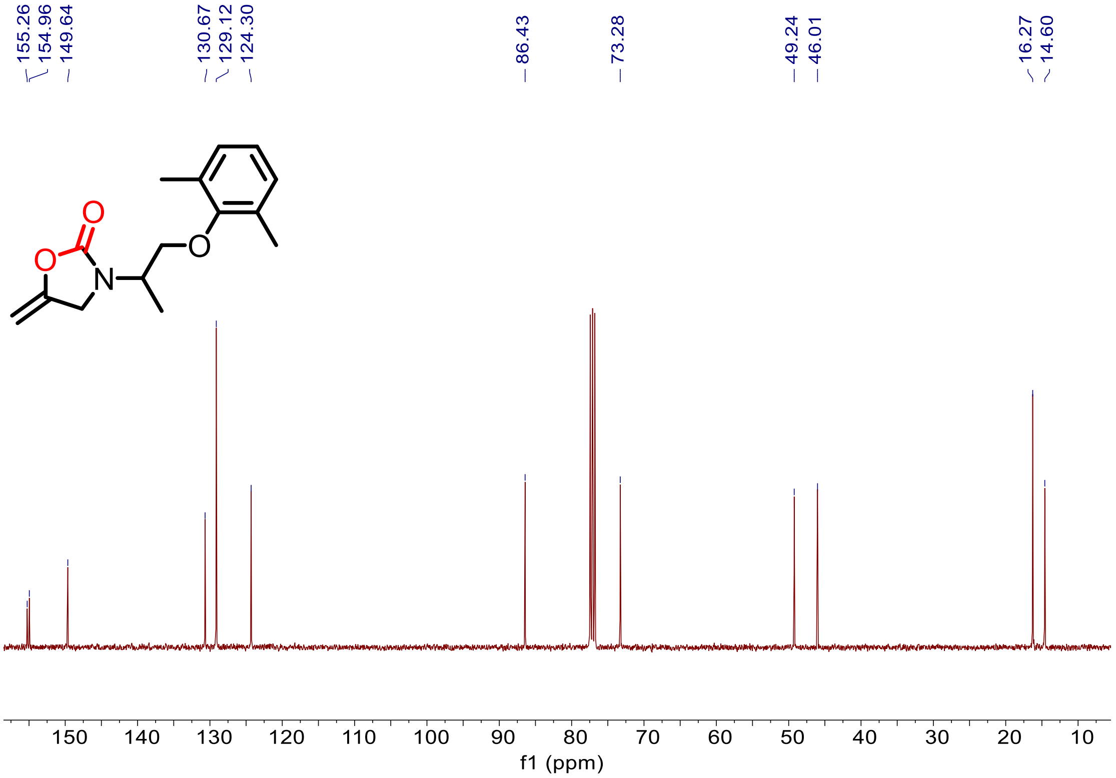
155.26 154.96 149.64
130.67 129.12 124.30
86.43
73.28
49.24 46.01
16.27 14.60
O
O
N
O
150 140 130 120 110 100 90 80 70 60 50 40 30 20 10
f1 (ppm)

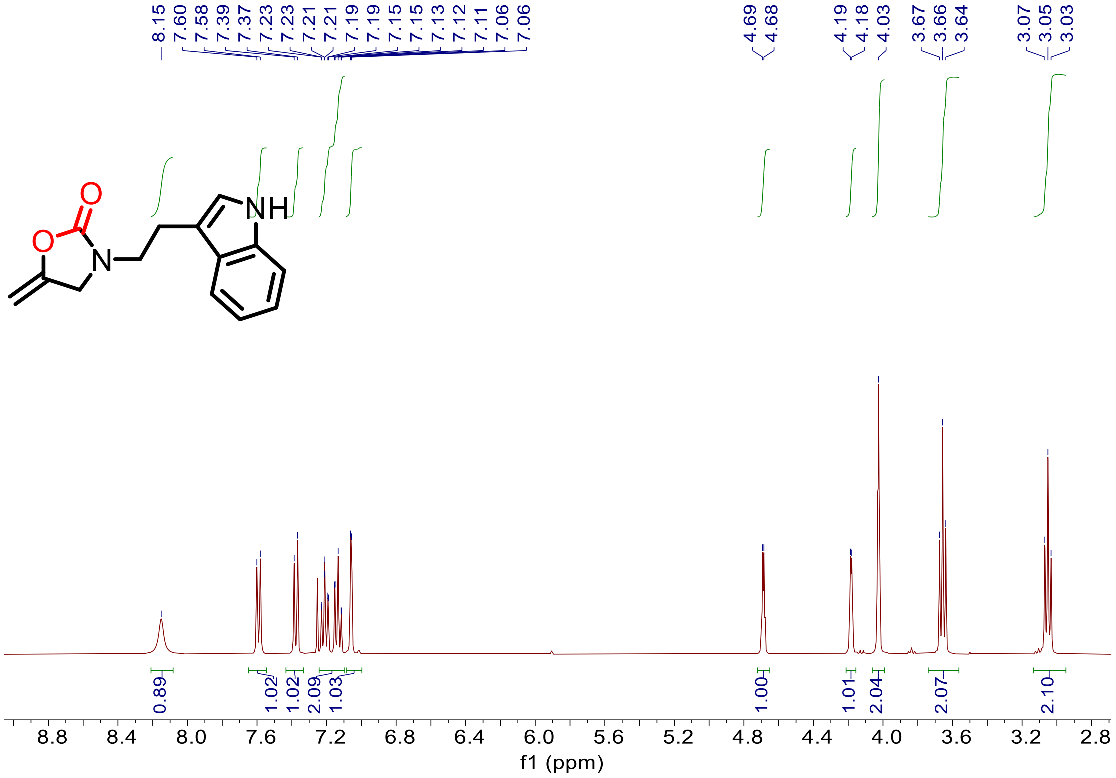
8.15
7.60 7.58 7.39 7.37 7.23 7.23 7.21 7.21 7.19 7.19 7.15 7.15 7.13 7.12 7.11 7.06 7.06
4.69 4.68
4.19 4.18 4.03
3.67 3.66 3.64
3.07 3.05 3.03
O
O
N
NH
0.89
1.02
1.02
2.09
1.03
1.00
1.01
2.04
2.07
2.10
8.8 8.4 8.0 7.6 7.2 6.8 6.4 6.0 5.6 5.2 4.8 4.4 4.0 3.6 3.2 2.8
f1 (ppm)

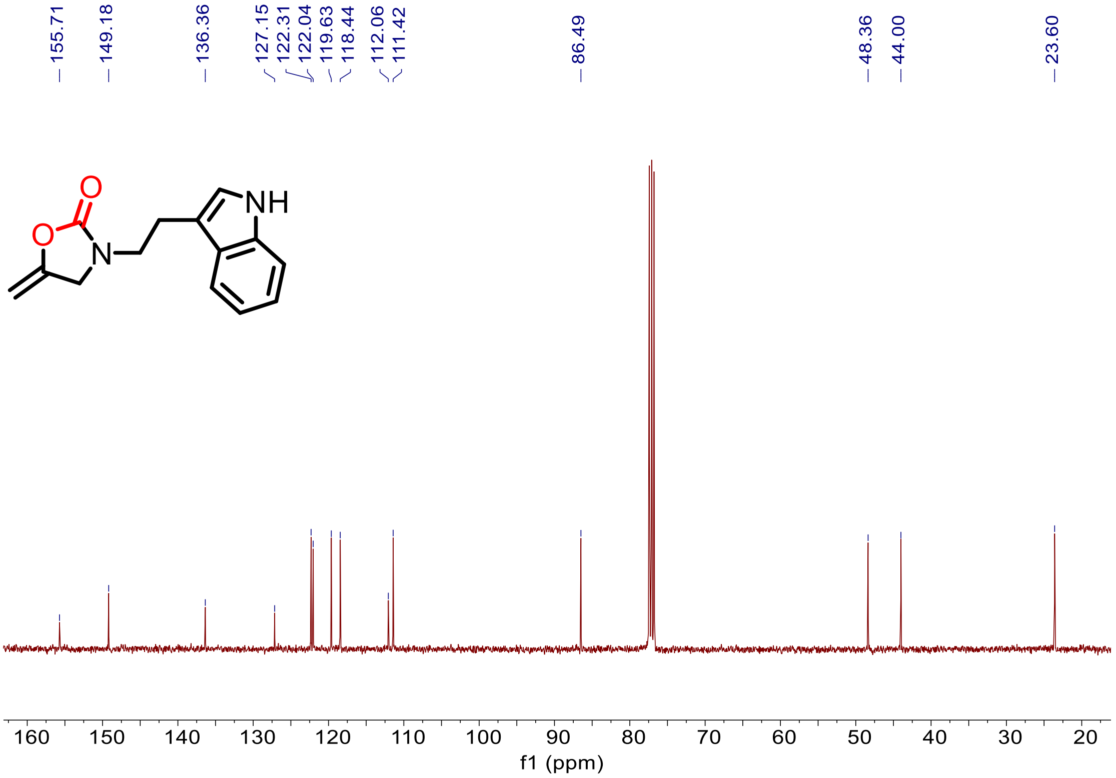
155.71
149.18
136.36
127.15 122.31 122.04 119.63 118.44
112.06 111.42
86.49
48.36
44.00
23.60
O
O
N
NH
160 150 140 130 120 110 100 90 80 70 60 50 40 30 20
f1 (ppm)

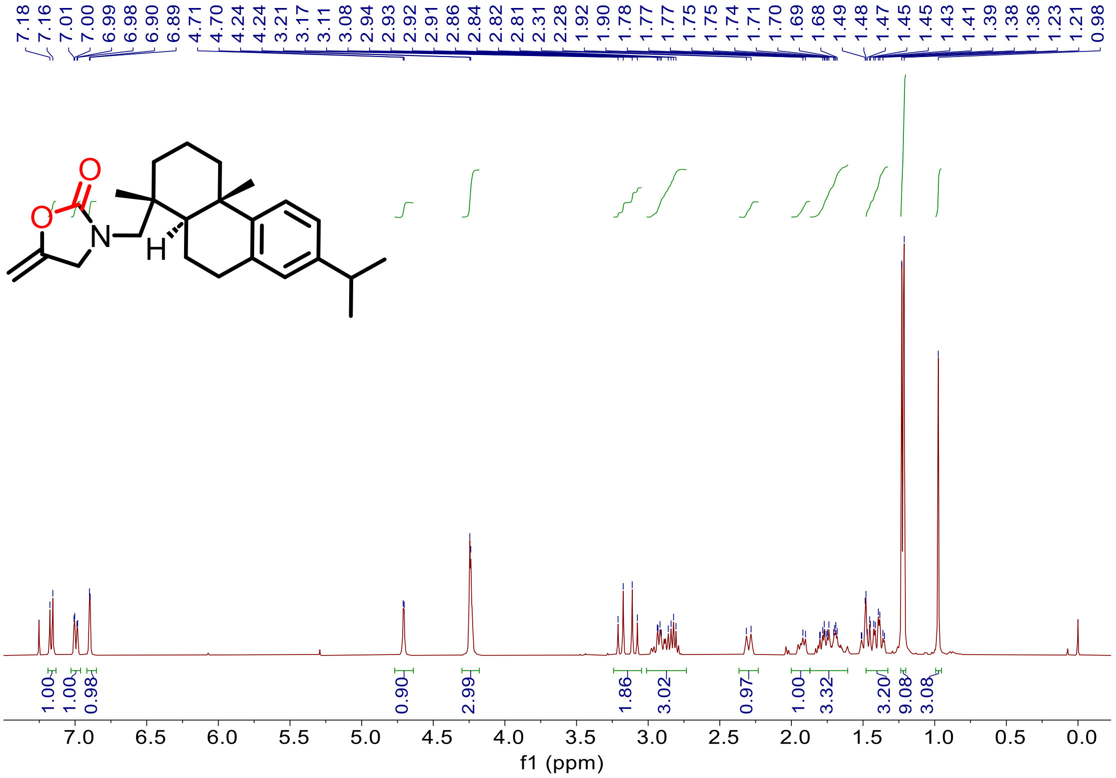
7.18 7.16 7.01 7.00 6.99 6.98 6.90 6.89 4.71 4.70 4.24 4.24 3.21 3.17 3.11 3.08 2.94 2.93 2.92 2.91 2.86 2.84 2.82 2.81 2.31 2.28 1.92 1.90 1.78 1.77 1.77 1.75 1.75 1.74 1.71 1.70 1.69 1.68 1.49 1.48 1.47 1.45 1.45 1.43 1.41 1.39 1.38 1.36 1.23 1.21 0.98
O
O
N
H
1.00 1.00 0.98 0.90 2.99 1.86 3.02 0.97 1.00 3.32 3.20 9.08 3.08
7.0 6.5 6.0 5.5 5.0 4.5 4.0 3.5 3.0 2.5 2.0 1.5 1.0 0.5 0.0
f1 (ppm)

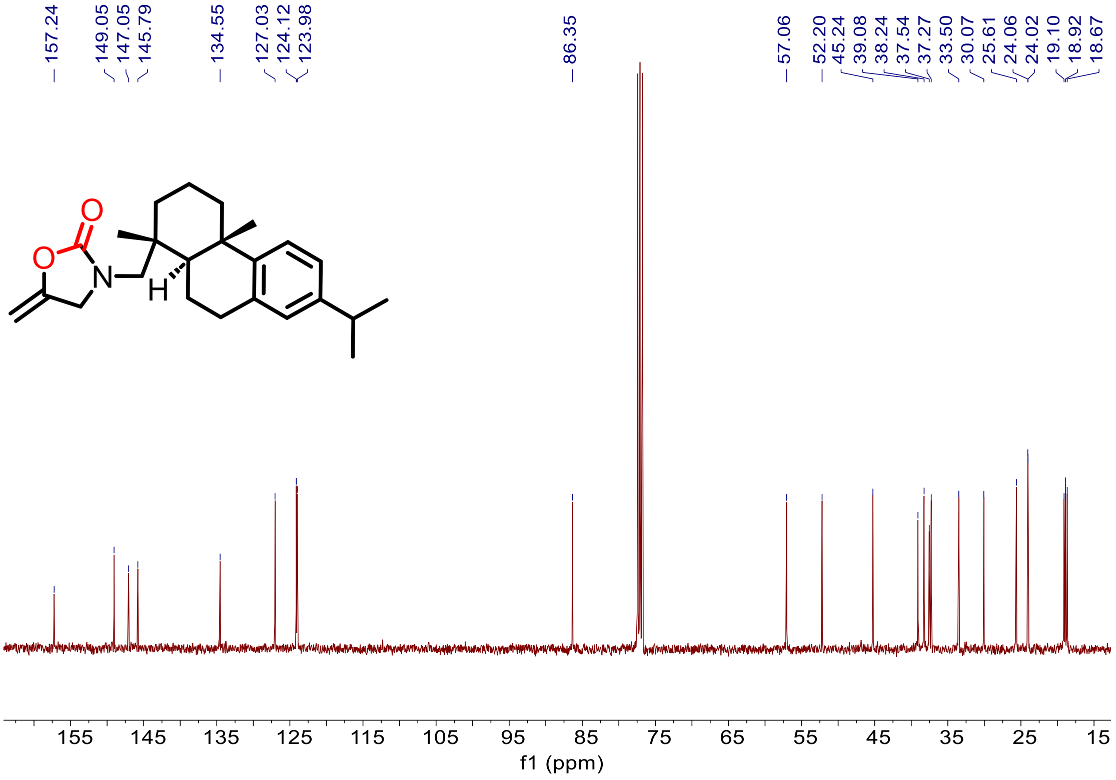
157.24 149.05 147.05 145.79 134.55 127.03 124.12 123.98 86.35 57.06 52.20 45.24 39.08 38.24 37.54 37.27 33.50 30.07 25.61 24.06 24.02 19.10 18.92 18.67
O
O
N
H
155 145 135 125 115 105 95 85 75 65 55 45 35 25 15
f1 (ppm)

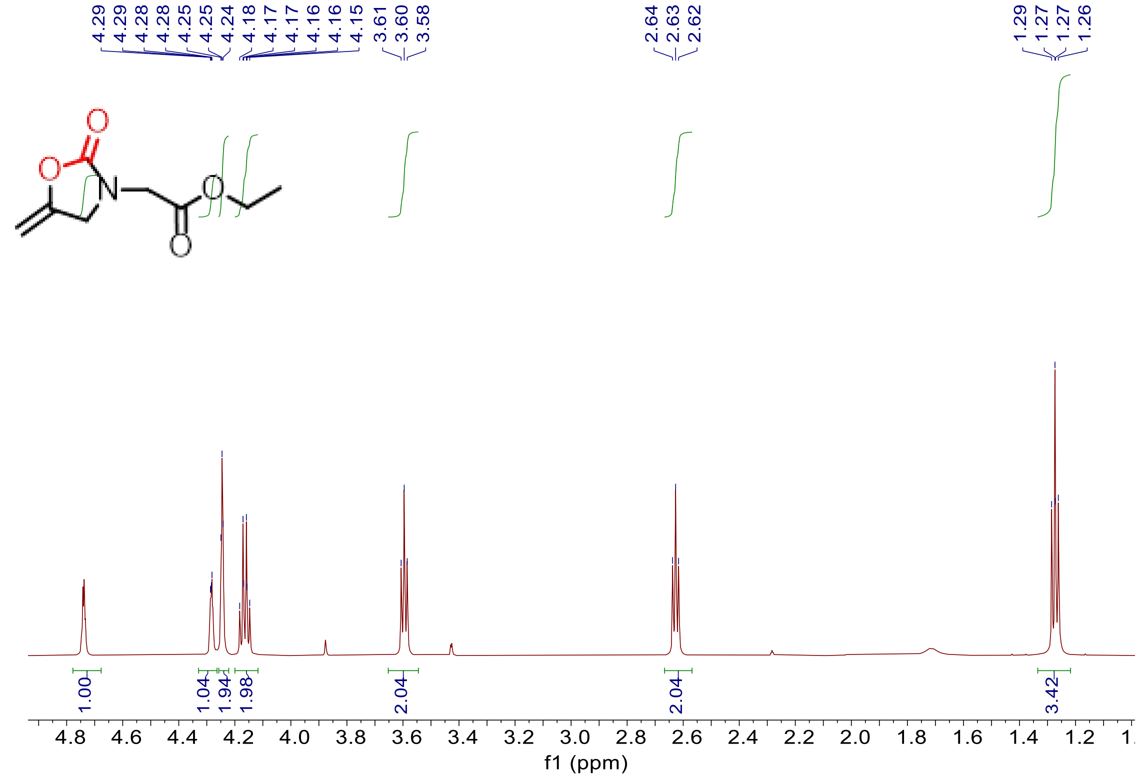
4.29
4.29
4.28
4.28
4.25
4.25
4.24
4.18
4.17
4.17
4.16
4.16
4.15
3.61
3.60
3.58
2.64
2.63
2.62
1.29
1.27
1.27
1.26
O
O
N
O
O
1.00
1.04
1.94
1.98
2.04
2.04
3.42
4.8 4.6 4.4 4.2 4.0 3.8 3.6 3.4 3.2 3.0 2.8 2.6 2.4 2.2 2.0 1.8 1.6 1.4 1.2
f1 (ppm)

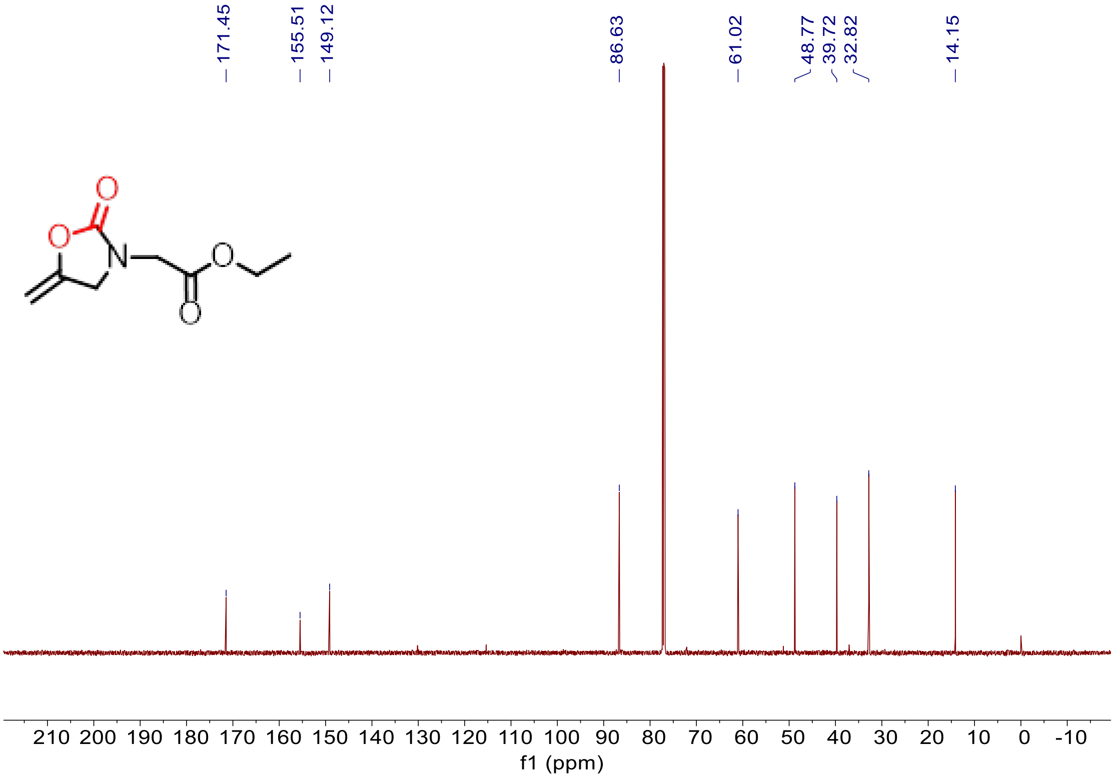
171.45
155.51
149.12
86.63
61.02
48.77
39.72
32.82
14.15
O
O
N
O
O
210 200 190 180 170 160 150 140 130 120 110 100 90 80 70 60 50 40 30 20 10 0 -10
f1 (ppm)

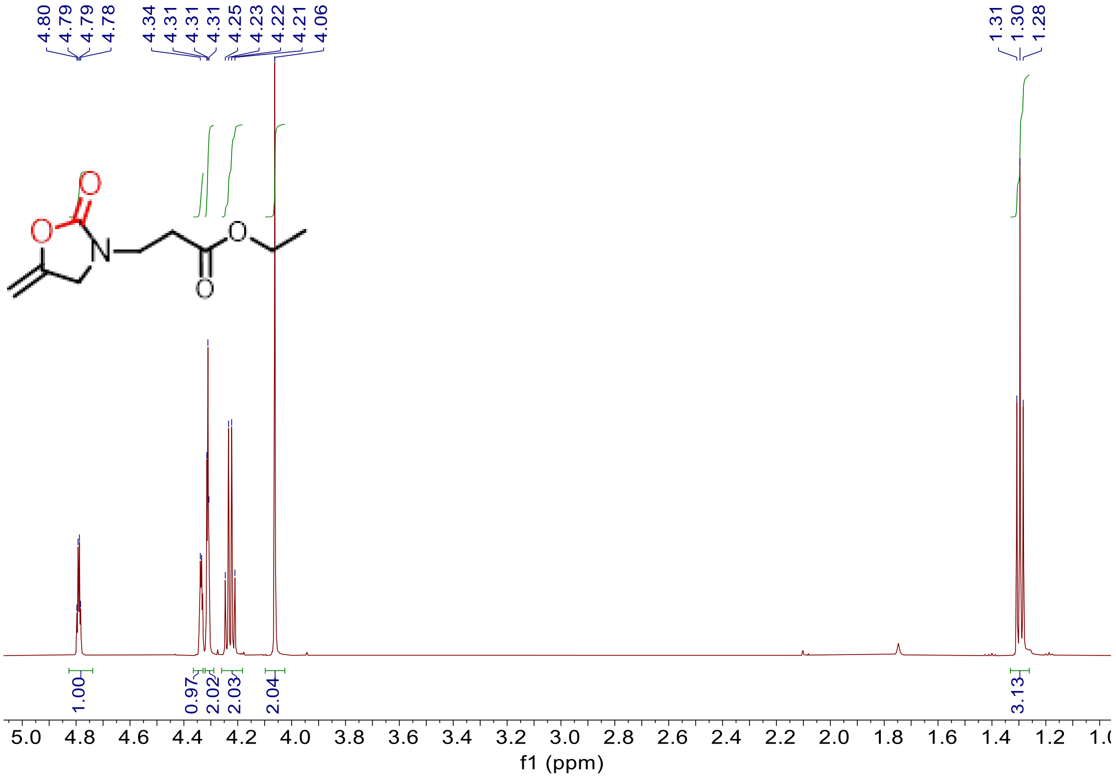
4.80
4.79
4.79
4.78
4.34
4.31
4.31
4.31
4.25
4.23
4.22
4.21
4.06
1.31
1.30
1.28
1.00
0.97
2.02
2.03
2.04
3.13
O
O
N
O
O
5.0 4.8 4.6 4.4 4.2 4.0 3.8 3.6 3.4 3.2 3.0 2.8 2.6 2.4 2.2 2.0 1.8 1.6 1.4 1.2
f1 (ppm)

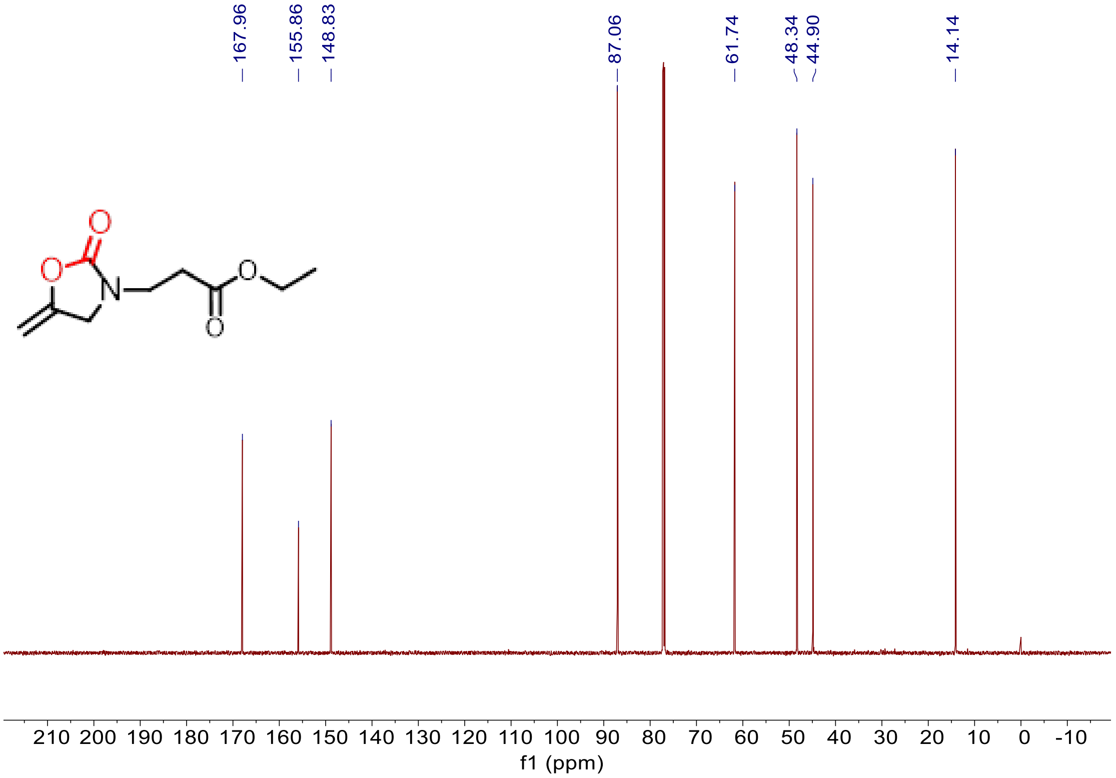
167.96
155.86
148.83
87.06
61.74
48.34
44.90
14.14
O
O
N
O
O
210 200 190 180 170 160 150 140 130 120 110 100 90 80 70 60 50 40 30 20 10 0 -10
f1 (ppm)

Section 5. Supporting References

[1] Almansaf Z, Hu J, Zanca F, Shahsavari HR *et al.* Pt(II)-Decorated Covalent Organic Framework for Photocatalytic Difluoroalkylation and Oxidative Cyclization Reactions. *ACS Appl Mater Interfaces* 2021; **13**: 6349-6358.

[2] Kremennaya MA, Soldatov MA, Budnyk AP *et al.* Theoretical and experimental study of mononuclear Cu(II) acetate-bipyridine complex. *J Struct Chem* 2016; **57**: 1348-1354.

[3] Cao CS, Xia SM, Song ZJ *et al.* Highly Efficient Conversion of Propargylic Amines and CO_2 Catalyzed by Noble-Metal-Free [Zn_{116}] Nanocages. *Angew Chem Int Ed* 2020; **59**: 8586-8593.

[4] Nuriya M, Ashikari Y, Iino T *et al.* Alkyne-Tagged Dopamines as Versatile Analogue Probes for Dopaminergic System Analysis, *Anal Chem* 2021; **93**: 9345-9355.

[5] Rao ZX, Chen PB, Xu J *et al.* Direct Conversion of CO_2 in Lime Kiln Waste Gas Catalyzed by a Copper-Based N-heterocyclic Carbene Porous Polymer, *ChemSusChem* 2023; **16**: e202300170.

[6] Li Y, Zhang D, Ye J *et al.* A Modular Tubular Flow System with Replaceable Photocatalyst Membranes for Scalable Coupling and Hydrogenation. *Angew Chem Int Ed* 2023; **62**: e202302979.

[7] Frisch MJ, Trucks GW, Schlegel HB *et al.* S77 Gaussian 09, Revision D.02, Gaussian, Inc., Wallingford CT 2009.

[8] Miertuš S, Tomasi J. Approximate evaluations of the electrostatic free energy and internal energy changes in solution processes. *Chem Phys* 1982; **65**: 239-245.

[9] Miertuš S. Electrostatic interaction of a solute with a continuum. A direct utilizaion of AB initio molecular potentials for the prevision of solvent effects. *Chem Phys* 1981; **55**: 117-129.

[10] Zhao Y, Truhlar DG. The M06 suite of density functionals for main group thermochemistry, thermochemical kinetics, noncovalent interactions, excited states, and transition elements: two new functionals and systematic testing of four M06-class functionals and 12 other functionals. *Theor Chem Acc* 2008; **120**: 215-241.

[11] Binning Jr. RC, Curtiss LA. Compact contracted basis sets for third-row atoms: Ga–Kr. *J. Comp Chem* 1990; **11**:1206-1216.
